# The m6A methylation landscape stratifies hepatocellular carcinoma into 3 subtypes with distinct metabolic characteristics

**DOI:** 10.20892/j.issn.2095-3941.2020.0402

**Published:** 2020-12-15

**Authors:** Xiaotian Shen, Beiyuan Hu, Jing Xu, Wei Qin, Yan Fu, Shun Wang, Qiongzhu Dong, Lunxiu Qin

**Affiliations:** 1 Department of General Surgery, Huashan Hospital & Cancer Metastasis Institute & Institutes of Biomedical Sciences, Fudan University, Shanghai 250040, China; 2 Institutes of Biomedical Sciences, Fudan University, Shanghai 200032, China; 3 Shanghai Medical College, Fudan University, Shanghai 200040, China

**Keywords:** Hepatocellular carcinoma, RNA N6-methyladenosine, metabolism, bioinformatics, prognosis

## Abstract

**Objective:**

Epigenetic aberration plays an important role in the development and progression of hepatocellular carcinoma (HCC). However, the alteration of RNA N6-methyladenosine (m6A) modifications and its role in HCC progression remain unclear. We therefore aimed to provide evidence using bioinformatics analysis.

**Methods:**

We comprehensively analyzed the m6A regulator modification patterns of 605 HCC samples and correlated them with metabolic alteration characteristics. We elucidated 390 gene-based m6A-related signatures and defined an m6Ascore to quantify m6A modifications. We then assessed their values for predicting prognoses and therapeutic responses in HCC patients.

**Results:**

We identified 3 distinct m6A modification patterns in HCC, and each pattern had distinct metabolic characteristics. The evaluation of m6A modification patterns using m6Ascores could predict the prognoses, tumor stages, and responses to sorafenib treatments of HCC patients. A nomogram based on m6Ascores showed high accuracy in predicting the overall survival of patients. The area under the receiver operating characteristic curve of predictions of 1, 3, and 5-year overall survivals were 0.71, 0.69, and 0.70 in the training cohort, and in the test cohort it was 0.74, 0.75, and 0.71, respectively. M6Acluster C1, which corresponded to hypoactive mRNA methylation, lower expression of m6A regulators, and a lower m6Ascore, was characterized by metabolic hyperactivity, lower tumor stage, better prognosis, and lower response to sorafenib treatment. In contrast, m6Acluster C3 was distinct in its hyperactive mRNA methylations, higher expression of m6A regulators, and higher m6Ascores, and was characterized by hypoactive metabolism, advanced tumor stage, poorer prognosis, and a better response to sorafenib. The m6Acluster, C2, was intermediate between C1 and C3.

**Conclusions:**

HCCs harbored distinct m6A regulator modification patterns that contributed to the metabolic heterogeneity and diversity of HCC. Development of m6A gene signatures and the m6Ascore provides a more comprehensive understanding of m6A modifications in HCC, and helps predict the prognosis and treatment response.

## Introduction

Hepatocellular carcinoma (HCC) is highly malignant and is characterized by a high recurrence rate, drug resistance, and poor prognosis. Many biological alterations, known as cancer hallmarks, occur during cancer development and progression, including but not limited to metabolic, immunogenic, and proliferative changes. It is therefore essential to understand what drives these dysregulations and how to deal with them^1–5^.

How RNA modification promotes cancer development has recently drawn attention^6^. N6-methyladenosine (m6A) is the most abundant RNA modification in eukaryotic cells^7,8^. Three types of proteins are responsible for m6A modification, i.e., writers, erasers, and readers, by adding, removing, or recognizing m6A-modified sites and altering important biological processes, respectively^9,10^. Many m6A regulators have been identified, including METTLs, WTAP, KIAA1429, and PC1F1 as writers; FTO, ALKBH5, and ALKBH3 as erasers; and YTHDFs, YTHDCs HNRNP, and IGF2BPs^11,12^ as readers^13–21^. Abnormal modification patterns of m6A regulation have been linked to cancer hallmarks such as the epithelial-mesenchymal transition (EMT), invasion, metastasis, and drug resistance of various cancers^22–28^. Notably, a recent study revealed that m6A modification can regulate the tumor microenvironment (TME) and can be decisive in determining the response to PD-1 antibody^22,29,30^. Altered m6A regulation patterns have been reported in HCC. Dysregulations of m6A regulators, including YTHDF2, WATP, KIAA1429, and YTHDF1, have been shown to facilitate HCC growth and progression^31–35^. However, an overall description of the m6A modification patterns in HCC is lacking, and how these patterns influence biological behaviors, especially whether there is a preferred biological process targeted by m6A regulation in HCC, remains elusive.

Metabolism alterations consist of important hallmarks and contribute to the heterogeneity of cancer. Such alterations are driven by a combination of genetic lesions and nongenetic factors. For example, pathway activation, TME, and even deoxidation and an acidic environment will alter cancer cell metabolism and facilitate its development and progression^36,37^. However, there has been no specific study focused on how m6A regulates cancer metabolism. A few studies of cancer cells or non-cancer cells have revealed that m6A is able to modulate metabolism. In colorectal carcinogenesis, methyltransferase METTL3 is able to stabilize GLUT1, and further enhance glycolysis^38^. In the liver, m6A RNA methylation is important for circadian regulation of downstream genes and lipid metabolism, and METTL3 knockdown is able to increase lipid metabolism through upregulating PPARA^39^. Moreover, in skeletal muscle cells, an inverse correlation between m6A methylation level and cellular lipid droplets was found^40^. Collectively, these results indicate a novel mechanism for driving metabolic alterations in cancer.

In this study, we identified 3 distinct m6A modification patterns in HCC, with distinct expression levels of m6A regulators. We elucidated m6A gene signatures and defined an m6Ascore to quantify the m6A modification pattern and established its value in predicting prognosis and therapeutic responses of HCC patients.

## Material and methods

### HCC data source and preprocessing

Public gene expression data, copy number variation data, DNA methylation data, and clinical annotations were collected from The Cancer Genome Atlas (TCGA) database, the Gene-Expression Ominibus (GEO), and the International Cancer Genome Consortium (ICGC). Patients without survival information were excluded. In total, 4 cohorts were enrolled in this study: TCGA-LIHI (369 samples), ICGC-LIRI-JP (225 samples), GSE14520 (221 samples), and GSE109221 (67 samples). For RNA-seq data (TCGA and ICGC cohorts), fragments per kb of transcript per million mapped reads [FPKM-normalized, log_2_-transformed data were downloaded from respective websites (*https://icgc.org/, https://portal.gdc.cancer.gov/*)] and then merged into one meta-cohort after removing the batch effect *via* the “sva” R package. Somatic mutation data were acquired from TCGA database. GEO microarray data were downloaded from the GEO website (*https://www.ncbi.nlm.nih.gov/geo/*) with the R package “geoquery.” The raw data in .CEL files were read and processed *via* the “limma” R package, and the gene expression levels were quantile-normalized (flowchart).

### Unsupervised clustering for m6A regulators

Twenty-three m6A regulators were identified *via* word-mining from PubMed, including 8 writers (METTL3, METTL14, RBM15, RBM15B, WTAP, KIAA1429, CBLL1, and ZC3H13), 2 erasers (ALKBH5 and FTO), and 13 readers (YTHDC1, YTHDC2, YTHDF1, YTHDF2, YTHDF3, IGF2BP1, IGF2BP2, IGF2BP3, HNRNPA2B1, HNRNPC, FMR1, LRPPRC, and ELAVL1), which were included in this study. We chose the m6A regulators based on word-mining from published reports, mostly from PubMed, and identified m6A regulators in HCC, including WTAP, YTHDF1, YTHDF2, YTHDF3, FTO, KIAA1429, YTHDF1, METTL3, METTL14, ALKBH5, YTHDC1, YTHDC2, and HNRNPA2B1^12,31–35,41,42^, containing 13 of the 23 m6A regulators that were included in our study. These regulators were comprised of writers, erasers, and readers and were sufficient for the following analyses^43,44^. For a complete overall description of m6A regulators dysregulated in HCC, we included other m6A regulators that had not yet been linked to HCC, but had been linked to other cancers, e.g., breast cancer or gastric cancer.

Clustering was performed in the meta-cohort merged from TCGA-LIHC cohort and the ICGC-LIRI-JP cohort to avoid different data distribution between RNAseq and microarray derived data. R package “Nbcluster” was used to determine the optimum number of clusters, with the following: min.nc = 2, max.nc = 15, and method = “kmeans.” R package “Kmeans” was used to perform K-means clustering and decided the cluster with: centers = 3, nstart = 25.

### Unsupervised clustering for metabolic subgroups in HCC

Gene set variation analysis (GSVA) enrichment scores for 50 metabolism-associated pathways were used for clustering. The metabolism-associated pathways (**Supplementary Table S3**) were selected from gene sets (e.g., GOBP, KEGG, and Biocarta) downloaded from the molecular signature database (MsigDB) (*https://www.gsea-msigdb.org/gsea/msigdb/index.jsp*). The same procedures used to derive the m6Aclusters were used to perform the k-means clustering for the metabolic subgroups in HCC.

### GSVA and functional annotation

To investigate the differences in biological process between m6Aclusters with m6A modification patterns, we performed GSVA enrichment analysis using the “GSVA” R package^45^. GSVA, which is based on a nonparametric and unsupervised method, is commonly used to estimate variations in the activities of pathways and biological processes in samples from an expression dataset. The KEGG, GOBP, and HALLMARK gene sets were downloaded from the MSigDB database to run the GSVA analysis. Adjusted values of *P* < 0.05 were considered statistically significant. The “clusterProfiler” R package was used to perform functional annotation for m6A gene signatures or other genes with a cutoff value of FDR < 0.05.

### Identification of differentially expressed genes (DEGs) between m6A distinct phenotypes

To identify m6A-related genes, we classified the patients into 3 distinct m6A modification patterns based on the expression levels of 21 m6A regulators. The empirical Bayesian approach in the “limma” R package was used to determine DEGs between different modification patterns. The significance criteria for determining DEGs was set at an adjusted value of *P* < 0.05, log_2_ fold change > 1, or < −1.

### Definition of m6A gene signatures and m6Ascores

We defined an m6Ascore to quantify the m6A modification pattern in HCC as follows. Univariate COX regression analysis was performed on the DEGs between 3 m6Aclusters with different m6A regulator modification patterns. Genes with significant prognoses were extracted for further analysis and were named m6A gene signatures. We then divided the genes into 2 groups according to the hazard ratio (HR) with a cut-off of HR = 1. Next, all genes were scaled between -1 to 1 to decrease the effect of gene expression values on the m6Ascore. We then defined the m6Ascore as the difference between the sum of the group with HR > 1 and the sum of group with HR < 1:

$$m\text{6}Ascore=\sum_{i}Xi-\sum_{j}Yj\;X\in Ci\;Y\in Cj$$

where Ci is the collection of scaled expression levels of m6A gene signatures with HR > 1, and Cj is the collection of scaled expression levels of m6A gene signatures with HR < 1.

### Connection between m6A regulator modification pattern clusters and other molecular stratifications in HCC

Alluvial diagrams were used to show the connections between m6A modification patterns and other molecular stratifications in HCC, including metabolic subgroups, tumor-node-metastasis (TNM) stage, iCluster, and iHCC. TCGA reported 3 integrated HCC clusters (i.e., iCluster1 to iCluster3). These clusters included a poor prognosis subtype (iCluster1), which had a gene expression profile closely resembling that of the progenitor cell subclass tumors, and a lower grade subtype (iCluster2), which included CTNNB1 mutations and less frequent microvascular invasions. The third TCGA cluster, iCluster3, generated a TP53 signature associated with chromosomal instability and poor prognosis^46^. Bidkhori et al.^47^ reported metabolic network-based stratification of HCC, with levels named iHCC1, iHCC2, and iHCC3. The iHCC1 and iHCC2 displayed upregulated metabolic biology, including glutamine metabolism, lipid metabolism and transport, and oxidative demethylation. The iHCC3 was not metabolically hyperactive, but it displayed upregulation of multiple processes associated with cell proliferation, cell cycle progression and mitosis, development, chromosome segregation, cytoskeleton organization, immune responses, DNA replication, and recombination.

### Statistical analysis

Correlation coefficients were computed as Pearson’s correlations with R; survival analysis and Kaplan-Meier curves were generated with the “survival” R package, and log-rank tests were performed to examine significance. We adopted a univariate COX regression model to compute the HR of genes and a multivariate COX regression model to compute the HR of prediction factors, including m6Ascore and other clinical traits with the “survival” R package. Receiver operating characteristic (ROC) curves and AUC values (area under the ROC curve) for the regression model were quantified using the “pROC” R package. All statistical *P* values were two-tailed, with *P* < 0.05 set as statistically significant. All data processing was conducted using R 3.6.3 software (The R Project for Statistical Computing, Vienna, Austria).

## Results

### Landscape of the genetic variations in m6A regulators in HCC

**Figure 1:**
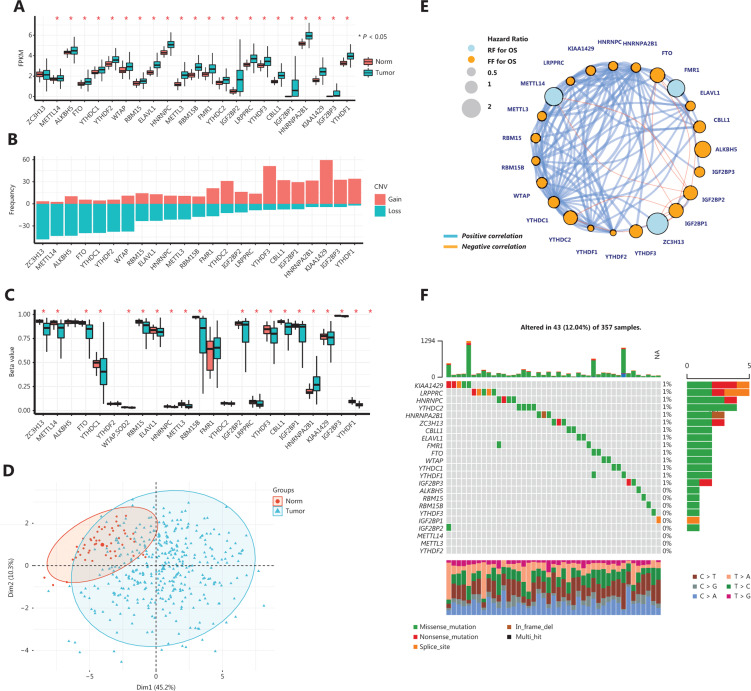
Expression and genetic landscapes of m6A regulators in hepatocellular carcinoma (HCC). A. Expression levels of m6A regulators in tumors and para-tumor normal tissues in HCC. All values are log_2_-transformed, fragments per kilobase of transcript per million mapped reads-normalized counts. B. Copy number variation frequency of 23 m6A regulators in The Cancer Genome Atlas (TCGA) cohort. C. Beta value of DNA methylation of 23 m6A regulators in TCGA cohort. D. Principle component analysis plot of the m6A regulator expression levels in tumors and para-tumor normal tissues. E. Cyclized plot showing the interaction between m6A regulators in HCC. Arcs represent Pearson’s correlations with *R* > 0.5 or *R* < -0.3, and the boldness of the arcs represents the Pearson’s correlation R value; the vertex color represents the hazard ratio of each m6A regulator on overall survival, and the circle size is relative to the hazard ratio. F. Somatic mutations in m6A regulator genes in TCGA cohort. Each column represents a patient with an m6A regulator mutation, and the upper panel shows the tumor mutation burden.

A total of 23 m6A regulators, including 8 writers, 2 erasers, and 13 readers, were examined in TCGA-LIHC cohort. We summarized the differences in the expression levels of the m6A regulators between tumor and non-tumor liver tissues. There was a remarkable upregulation of m6A regulators in tumor tissues compared with that in non-tumor liver tissues; furthermore, principle component analysis (PCA) confirmed a clear heterogeneity in the m6A regulator expression patterns between tumor and non-tumor liver tissues (**Figure 1A, 1D**). An investigation of copy number variation (CNV) showed a prevalent alteration of CNV in m6A regulators (**Figure 1B**). Regulators such as ZC3H13, METTL14, ALKNH5, FTO, YTHDC1, YTHDF2, and WTAP showed a majority of loss in alterations of CNV, while YTHDF3, CBLL1, IGF2BP1/3, HNRNPA2B1, KIAA1429, and YTHDF1 were mainly characterized by gain alterations. However, these results did not fully explain the upregulation of m6A regulators (**Figure 1A**), because gain/loss changes in m6A regulator genes were evenly distributed. Notably, analyses of 450K DNA methylation data of HCCs revealed a hypomethylation state of most m6A regulators, including those with a loss alteration of CNV, such as METTL14, ALKBH5, and FTO, which might have been the cause of hyperexpression of m6A regulators (**Figure 1C**). Based on the expression of m6A regulators, we could easily distinguish tumor tissues from normal tissues (**Figure 1D**). The expression patterns of m6A regulators were highly correlated with each other, as visualized in the cyclized plot showing correlations > 0.5 or < -0.3 (**Figure 1E**). Univariate COX regression model analyses showed that most m6A regulators were risk factors for a poorer prognosis, except for METTL14, ZC3H13, YTHDC2, and FMR1, which were protective factors for HCC (with HR < 1). At the genome stability level, only a few somatic mutations were detected in the m6A regulator-encoding genes in HCC, because only 41 out of 357 TCGA-LIHC cohort samples carried mutations. The most mutated m6A regulator gene was KIAA1429, with a percentage of 1% (**Figure 1F**). These results emphasized the highly diverse nature of the m6A regulator expression landscape in HCC, and showed that this variability had important effects on HCC prognosis and progression.

### The m6A methylation modification patterns in HCC

**Figure 2:**
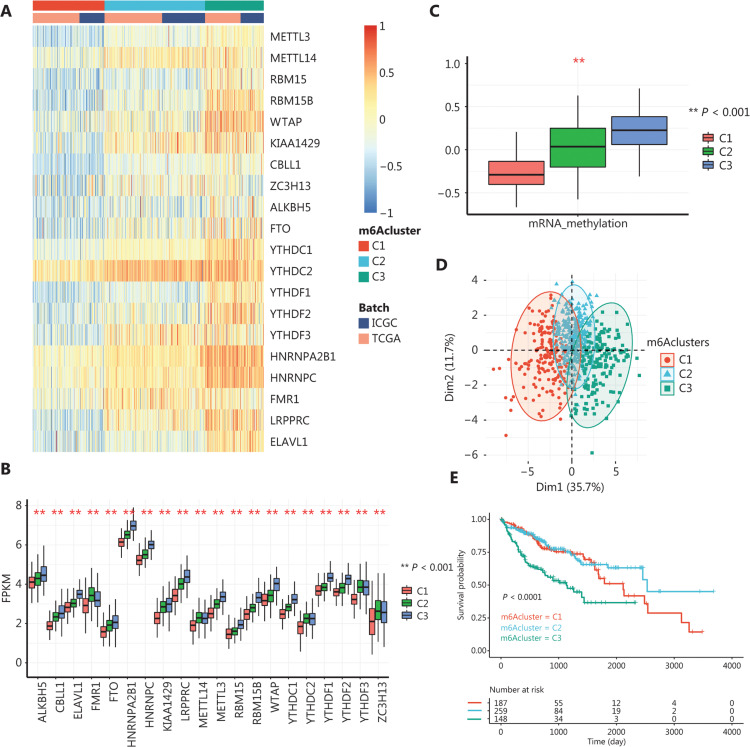
The m6A regulator modification patterns in hepatocellular carcinoma (HCC). A. The k-means clustering of m6A regulator expression in HCC revealed 3 clusters with different modification patterns. B. Gene set variation analysis enrichment scores of mRNA methylation among the 3 m6Aclusters. (**Student’s *t*-test; *P* < 0.05). C. Principle component analysis plot of m6A regulator expressions among the 3 m6Aclusters. D. Expressions of m6A regulators among the 3 m6Aclusters. The expression values are log-transformed, fragments per kilobase of transcript per million mapped reads-normalized counts. (**Student’s *t*-test; *P* < 0.05) E. Kaplan-Meier plot of patient overall survival among the 3 m6Aclusters. Significance was tested using the log-rank test.

A meta-HCC cohort consisted of 605 samples from TCGA-LIHC cohort and the ICGC-LIRI-JP cohort, and was used to make full use of the available data. Notably, only 20 of 23 m6A regulators were included in the clustering process, where IGF2BP families were removed as a result of data restriction. We next characterized the m6A regulator modification pattern in HCC. After k-means clustering, we identified 3 clusters with distinct m6A expression patterns, i.e., m6Aclusters C1−C3 (**Figure 2A**). The expression levels of the m6A regulators varied among the clusters. In general, clusters C1 and C3 had the lowest and highest m6A regulator expression levels, respectively. Clusters C1 and C2 mostly differed in their levels of m6A readers, such as YTHDCs and YTHDFs, while clusters C1 and C3 mostly differed in their levels of writers, such as WTAP and METTL13 (**Figure 2B**). We calculated the GSVA enrichment scores of the 3 clusters based on the mRNA methylation gene signatures in GOBP, to evaluate how m6A regulator expression patterns affected mRNA methylation. Cluster C1 and C3 had the lowest and highest mRNA methylation enrichment scores, respectively (**Figure 2C**), confirming that the differences in m6A regulator expression levels in the m6Aclusters affected the mRNA methylation levels. PCA analysis confirmed the robustness of the existence of these 3 patterns by showing that the 3 clusters could be categorized into 3 distinct groups (**Figure 2A, 2D**). Next, we determined how the m6A expression patterns affected the prognosis. We found that patients in the 3 clusters differed in overall survival (OS). Patients in clusters C1 and C2 had relatively better prognoses compared with those in cluster C3 (**Figure 2E**). These results indicated that there were different modification patterns of m6A RNA methylation in HCC, and that these patterns had prognostic value.

### Metabolic characteristics are distinct between m6A modification patterns

**Figure 3:**
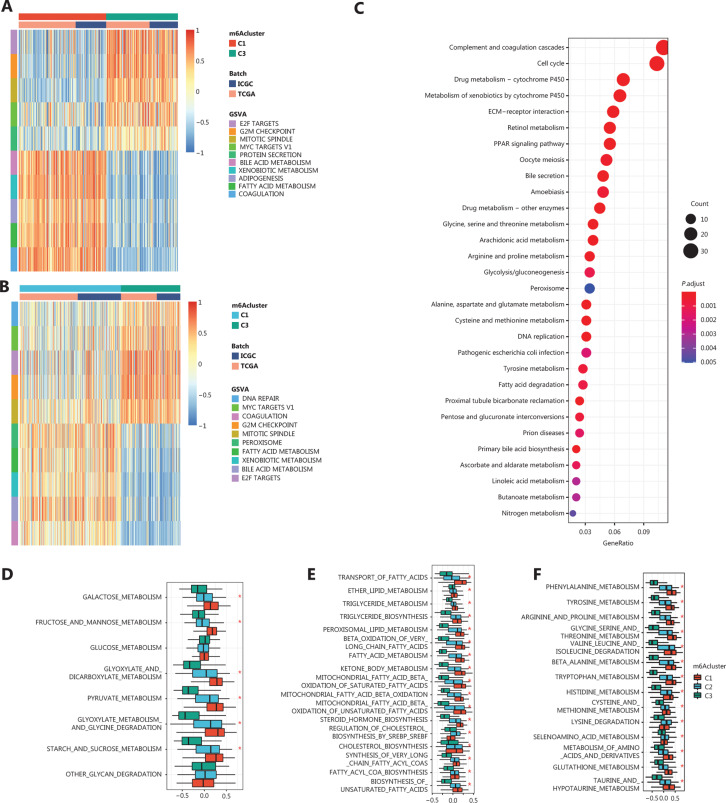
Biological characteristics of each m6A regulator modification pattern. A and B. Gene set variation analysis (GSVA) enrichment scores showing the activation state of cancer-associated pathways in m6A regulator modification patterns. The pathways are from the HALLMARK database. C. Functional annotation of differentially expressed genes using the Kyoto Encyclopedia of Genes and Genomes database. D−F. Comparison of GSVA enrichment scores of metabolism-associated gene sets among the 3 m6Aclusters. D. Carbohydrate metabolism. E. Opioid metabolism. F. Amino acid metabolism. (*Kruskal test; *P* < 0.05).

It is widely accepted that m6A regulators play roles in a variety of biological functions, including dysregulated cell death and proliferation, tumor malignancy, and immune modifications. To compare the biological functions affected by m6A regulator modification patterns among these clusters, we performed GSVA enrichment analysis with MsigDB gene sets. As shown in **Figures 3 and Supplementary Figure S1**, compared with the C3 cluster, clusters C1 and C2 were active in metabolic processes such as adipogenesis, fatty acid metabolism, and other carbohydrate or amino acid metabolic processes. In contrast, cluster C3 was enriched in methylation processes, regulation of the cell cycle, and proliferation processes (**Figure 3A and 3B**). We also computed the differentially-expressed genes (DEGs) among clusters and functionally annotated them. Consistently, KEGG annotation for the DEGs between clusters C1, C2, and C3 revealed similar outcomes to previous findings (**Figure 3C**). In the top 30 terms, we found an accumulation of terms related to metabolic pathways and cell cycles. To further compare metabolic biology processes, we compared the GSVA enrichment scores of metabolism-associated gene sets between the m6Aclusters, and found that cluster C1 was hyperactive in carbohydrate, lipid, and amino acid metabolism, while cluster C3 was hypoactive (**Figure 3D, 3E, and 3F**). These observations indicated that m6A regulators negatively regulated the metabolic state in HCC. These results also revealed a distinct metabolic pattern in HCC, which was related to m6A modification patterns. Compared with clusters C1 and C2, cluster C3, which showed hypoactive metabolism, was more closely associated with hyperactivity in the cell cycle and proliferation functions.

Metabolic reprogramming is a well-established hallmark of cancer. In HCC, metabolism, especially lipid metabolism is associated with OS^48^. To characterize the association between m6A modification patterns and metabolic alterations in HCC, we first word-mined PubMed for the target genes of m6A regulators in HCC or HCC-derived cell lines, which yielded 17 direct targets (**Supplementary Table S1**). A full annotation is shown in **Supplementary Table S2**. Gene annotation revealed an enrichment of terms related to metabolic homeostasis and cell division. The metabolism-related terms, among which carbohydrate metabolism and cholesterol metabolic processes ranked at the top, are shown as **Supplementary Figure S2**. These results indicated that m6A regulators had a role in altering metabolic processes in HCC.

**Figure 4:**
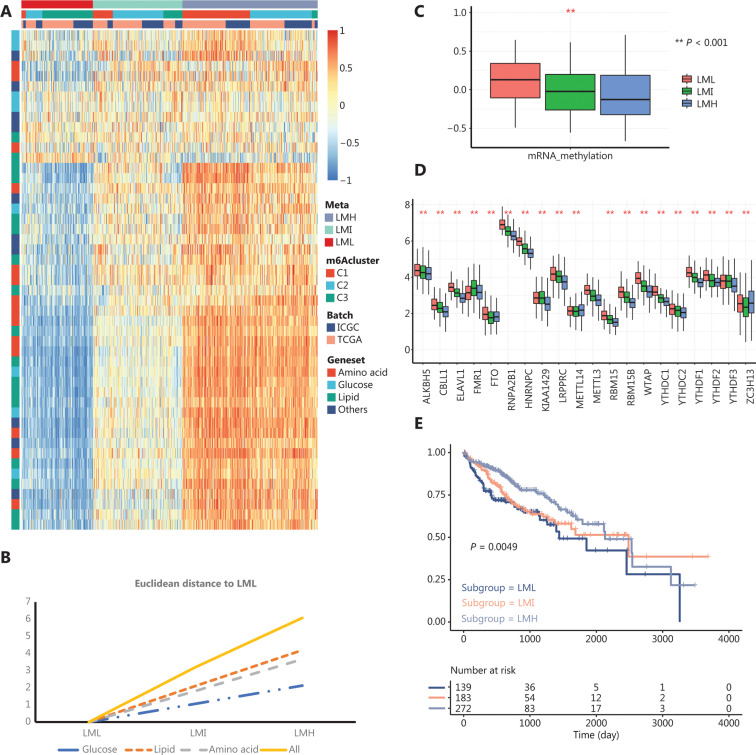
The m6A regulator modification patterns were associated with metabolic subgroups in hepatocellular carcinoma (HCC). A. Heat map showing that HCC could be stratified based on the gene set variation analysis (GSVA) enrichment scores of 4 major metabolic pathways, i.e., carbohydrate metabolism (glucose), lipid metabolism, amino acid metabolism, and other metabolism. The annotation panel shows the assignment of each sample to m6Aclusters or metabolic subgroups. B. The Euclidean distance of each major metabolic pathway between different metabolic subgroups was calculated to identify the most altered metabolic pathways. Among the 3 major metabolic pathways (other metabolism excluded), carbohydrate (glucose) metabolism showed the lowest variation among the metabolic subgroups while lipid metabolism showed the highest variation. C. GSVA enrichment scores of mRNA methylations among the 3 metabolic subgroups. (**Student’s *t*-test; *P* < 0.05) D. Expressions of m6A regulators among the 3 metabolic subgroups. The expression values are log_2_-transformed, fragments per kilobase of transcript per million mapped reads-normalized counts (**Student’s *t*-test; *P* < 0.05). E. Kaplan-Meier curves of patient overall survial in each metabolic subgroup. Significance was tested using the log-rank test. The metabolic HCC subgroups showed distinct m6A regulator modification patterns.

Next, we stratified the HCC samples based on the GSVA enrichment scores of 50 metabolism-associated biological processes mined from GO, KEGG, and BIOCARTA gene signatures centered on 4 major metabolic processes, i.e., carbohydrate (glucose) metabolism, lipid metabolism, amino acid metabolism, and other metabolism (**Supplementary Table S3**). Using the same k-means clustering method, we identified 3 clusters with different metabolic patterns in HCC (**Figure 4A**). By comparing the Euclidean distances of each major metabolic process in the 3 clusters, we found that lipid metabolism was the most highly altered metabolic process (**Figure 4B**). Moreover, clusters 1 and 3 had the lowest and highest enrichment scores for lipid metabolism, respectively. Accordingly, we termed clusters 1, 2, and 3 as the lipid metabolism low group (LML), the lipid metabolism intermediate group (LMI), and the lipid metabolism high group (LMH), respectively, (**Figure 4A**). Comparison of the GSVA enrichment scores of different biological processes among these 3 metabolic clusters showed similar differentially enriched biological processes to those observed in the m6Aclusters. More specifically, in the hypermetabolic LMH and LMI clusters, the differentially enriched biological processes were mostly related to metabolism, while for the hypometabolic LML cluster, cell cycle-related biological processes were enriched (**Supplementary Figure S3**). Furthermore, to validate the differences in the m6A regulator modification patterns among the metabolic subgroups, we compared the m6A regulator expression levels and enrichment scores of mRNA methylation between the metabolic subgroups. This comparison revealed that the LML cluster had the highest expression level of m6A regulators and the highest mRNA methylation enrichment score, while the LMH cluster had the lowest m6A regulator expression level and the lowest methylation enrichment score (**Figure 4C, 4D**). These observations indicated a negative correlation between m6A methylation and metabolic activity. Prognosis analyses showed a poorer OS in LML patients (**Figure 4E**). Collectively, these results showed that there was a close relationship between the m6A regulator modification pattern and metabolic processes in HCC.

### Generation and functional annotation of m6A gene signatures and definition of the m6Ascore

**Figure 5:**
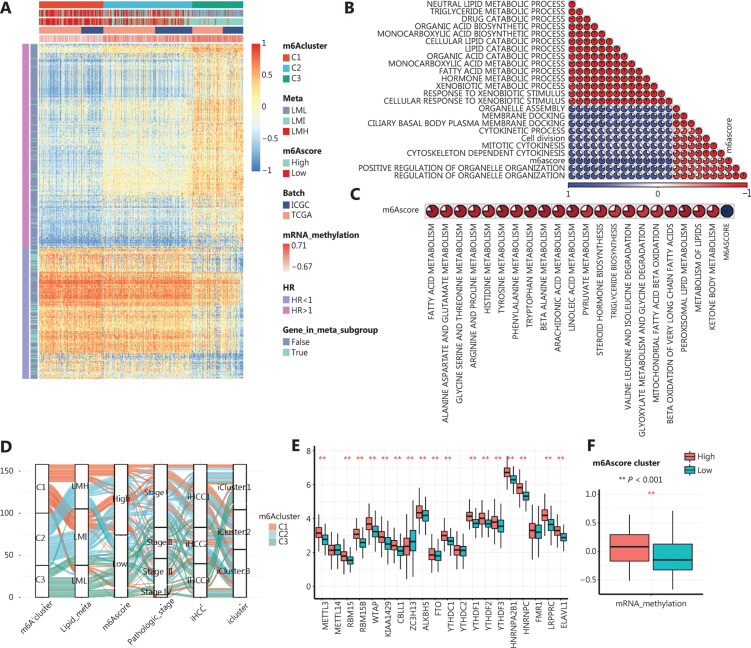
Generation of m6A signatures and m6Ascores A. A heat map showing the expression patterns of the m6A signatures. Column labels are m6Acluster, metabolic subgroup (meta cluster), m6Ascore batch, and gene set variation analysis (GSVA) enrichment scores of mRNA methylation. Row labels are the hazard ratios and whether a gene was included in the metabolic pathways in **Figure 4A**. B, C. Unsupervised clustering plot of Pearson’s correlation R values between the GSVA enrichment score and m6Ascore. Blue indicates an R value > 0, red indicates an R value < 0. Correlation between m6Ascore and gene set variation analysis enrichment score of lipid metabolism is shown in C. D. Alluvial diagram showing the connection between m6Acluster, metabolic subgroups (Lipid_meta), m6Ascore, pathological stage, iHCC cluster, and icluster cluster. E. Expressions of m6A regulators among two m6Ascore clusters. The expression values are log_2_-transformed, FPKM-normalized counts (**Student’s *t-*test; *P* < 0.05) F. GSVA enrichment scores of mRNA methylation among two m6Ascore clusters (**Student’s *t*-test; *P* < 0.05).

To further investigate the potential biological behaviors related to m6A regulator modification patterns, we generated m6A gene signatures and defined the m6Ascore using the following procedure. First, we identified the DEGs among the 3 m6Aclusters, then we discarded genes with univariate COX regression analysis *P* values > 0.05, leaving 390 genes as m6A signature genes. Univariate COX analysis of the DEGs is shown in **Supplementary Table S4**. Next, all m6A signature genes were normalized between -1 to 1, and the difference between the sum of the m6A signature genes with HR > 1 and the sum of the m6A signature genes with HR < 1 was calculated as the m6Ascore. The m6A gene signature reflected the intergroup differences among the m6Aclusters, because expression of the m6A gene signatures perfectly delineated 3 m6Aclusters within HCC; furthermore, the m6Ascore was highly correlated to the mRNA methylation enrichment score (**Figure 5A**, annotation panel).

Functional annotation of the m6A gene signatures showed remarkable enrichments in biological processes related to methylation and metabolism (**Supplementary Figure S4B, S4C**). To further investigate how the m6A gene signatures altered biological functions, we calculated Pearson’s correlations between the m6Ascore and the GSVA enrichment scores of 7,980 biological processes in the GOBP database. The excluded mRNA-related biological processes with the highest correlations with m6Ascore are shown in **Figure 5B**. These results showed that metabolic processes were strongly and negatively correlated with m6Ascore (**Figure 5B, 5C**), while cell cycle-related processes were positively correlated (**Figure 5B**). Overall, these results suggested significant roles for m6A regulators in metabolic regulation.

### Characteristics of clinical and therapeutic traits in m6Ascore clusters

We divided the HCC samples into an m6Ascore high cluster and an m6Ascore low cluster using the median m6Ascore as the cutoff. We then constructed an alluvial diagram to better visualize the patient attributes in existing HCC stratification methods (**Figure 5D**), which included a metabolic gene network-stratified method (iHCC) and a multi-platform integrative molecular subtype (iCluster)^46^. This analysis showed that m6Acluster C3 robustly corresponded with the LML cluster, higher m6Ascores, stage iii/iv, iHCC3, and iCluster 1, as generated by other stratification methods. We compared the m6A regulator expression levels between the m6Ascore low and m6Ascore high groups to ensure that the scoring system reflected the m6A regulator modification pattern in HCC (**Figure 5E, 5F**). This analysis revealed clear hyperexpression of m6A regulators and elevated mRNA methylation in the m6Ascore high group compared with these values in the m6Ascore low group.

**Figure 6:**
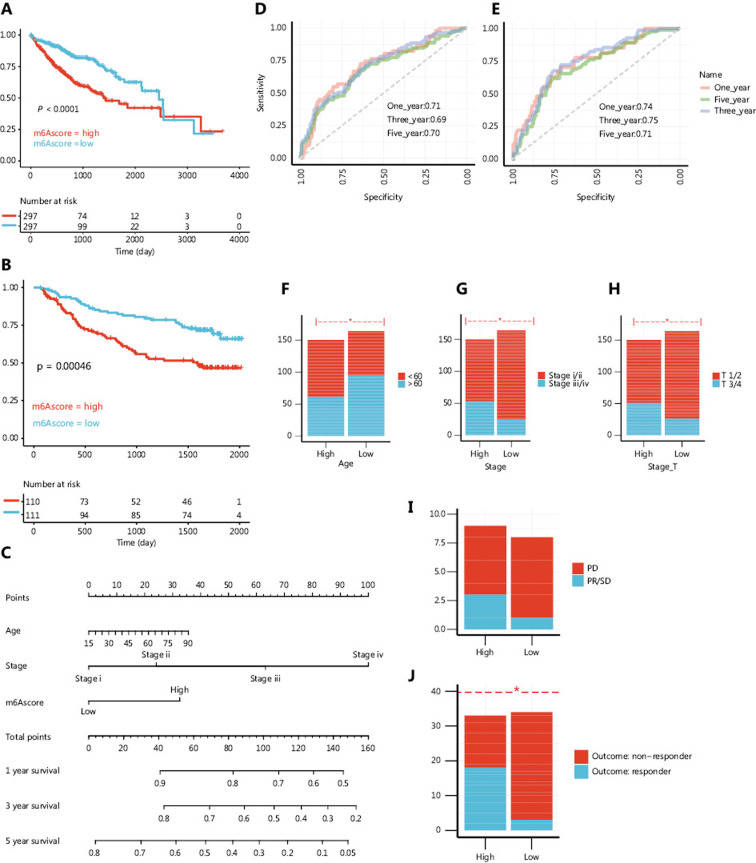
The m6Ascore is associated with clinical, prognostic, and therapeutic traits A,B. Kaplan-Meier curve showing the overall survival (OS) of m6Ascore high and m6Ascore low patients in the meta cohort (A) or the GSE14520 validation cohort (B). C. Nomogram for predicting of 1, 3, and 5-year OS of patients in The Cancer Genome Atlas (TCGA) cohort. D, E. Receiver operating characteristic curve for predicting the accuracy of the nomogram in TCGA cohort (D) and the GSE14520 cohort (E). F-H. Clinical demographics between the m6Ascore high and low patients. Age, stage, and, T stage were compared (*for the *χ*^2^ test; *P* < 0.05). I, J. Response to sorafenib treatment in the m6Ascore high and low patients in the STORM cohort (I) and TCGA cohort (J) (*for the *χ*^2^ test; *P* < 0.05).

Next, we further established the value of the m6Ascore in predicting patient outcomes, to investigate the connection between m6Ascores and clinical demographics. To test the m6Ascore in an alternative approach, we applied survival analysis in the meta cohort and validated it in a microarray-derived cohort (GSE14520). By using the median m6Ascore of each cohort as cutoffs, we divided the patients into an m6Ascore low group and an m6Ascore high group. The m6Ascore low patients had better OS compared with m6Ascore high patients in both the training cohort and in an independent validation cohort (GSE14520) (**Figure 6A, 6B**). We also tested whether m6Ascores could be used as an independent prognostic biomarker for HCC. A multivariate COX regression model including age, m6Ascore, and stage confirmed m6Ascore as a robust and independent prognostic marker for evaluating patient outcomes (HR = 1.33˜2.55, **Supplementary Figure S5A**). Moreover, a nomogram integrating the m6Ascore (high *vs*. low), stage, and age was constructed (**Figure 6C**). The point totals were calculated by adding up all points assigned to each factor. This nomogram was accurate in predicting OS. The AUC values for predicting 1, 3, and 5-year OS in the meta cohort were 0.71, 0.69, and 0.70, respectively, and in GSE14520 they were 0.74, 0.75, and 0.71, respectively (**Figure 6D, 6E**). In addition to survival, comparisons of other clinical traits showed that patients in the m6Ascore high group were younger and had higher stages (both stage T and TNM stage) (**Figure 6F, 6G and 6H**). These results confirmed that m6Ascore reflected both clinical and prognostic traits.

Next, we specifically examined the ability of the m6Ascore to predict the efficacy of sorafenib treatment. A total of 67 patients from the STORM cohort and 17 patients from TCGA cohort under sorafenib treatment were included in this analysis. In TCGA cohort, no significant differences were observed between the progressive disease (PD) and partial recession/stable disease (PR/SD) groups or between the m6Ascore high and m6Ascore low groups, probably due to the limited sample size; however, a weak tendency for PD patients and m6Ascore high patients to have a poorer OS was observed (**Supplementary Figure S5B, S5C**). Surprisingly, we found that m6Ascore low patients showed a higher response rate to sorafenib compared with m6Ascore high patients (**Figure 6I, 6J**). These results indicated that although m6Ascore high patients may had a higher response rate to sorafenib, this difference might not have affected the OS.

## Discussion

Increasing evidence supports a close link between m6A regulators and metabolic processes, including lipid^39,49–51^ and glucose metabolism^38,52–54^ in cancer and non-cancerous diseases. However, most of these studies focused on a single regulator, and the overall influence of m6A regulators on metabolism has not been fully established. Identification of the roles of m6A modification patterns in metabolic alterations in HCC will improve our understanding of the roles of m6A regulators and the metabolic heterogeneity of HCC, providing novel opportunities for developing effective therapies.

Overall, a significantly different expression pattern between tumor and para-tumor normal tissues was identified. Tumor tissues had a much higher expression of m6A methyltransferases than normal tissues, which is highly consistent with gastric cancer, breast cancer, glioma, colon cancer, and pancreatic cancer^55^. The expression difference was not caused by somatic mutations or copy number variations; unlike leukemia, clear cell renal cell carcinoma, and colorectal cancer, whose dysregulations of m6A methyltransferases may be caused by somatic mutations^56–58^. In HCC, these genes are rarely mutated, indicating that the expression alteration was not caused by somatic mutation. Regarding CNV, gain or loss alterations are prevalent among m6A regulators, which indicated that CNV might affect the expression in some ways. However, gain/loss was distributed equally among regulators, and the data were not convincing enough to conclude that CNV caused the upregulation of m6A regulators in HCC. Notably, we found a demethylation state of most m6A regulators, which may partially explain the expression difference between tumor and normal tissues.

In the present study, by focusing on 20 m6A regulators, we defined 3 distinct m6A modification patterns in HCC that correlated with different metabolic states. Cluster C1 comprised the lowest expressed m6A regulators and lowest mRNA methylation level, and correlated with the highest metabolic activity. Cluster C2 was associated with intermediately expressed m6A regulators and intermediate mRNA methylation, and correlated with moderate metabolic activity. Cluster C3 was associated with the highest expression levels of m6A regulators and highest mRNA methylation, and correlated with the lowest metabolic activity. Among all major metabolic categories, lipid metabolism was the most heterogeneous in HCC and had the strongest correlation with m6Ascore, suggesting that lipid metabolism was the most likely target of m6A regulators in HCC. Prognosis analyses show that C1 patients have the longest OS, while C3 patients have the shortest, which is consistent with a previous finding that lipid-metabolic active HCC patients have better prognoses and lower tumor grades^46,47^.

While analyzing the data, we observed an interesting phenomenon in a variety of solid tumors, e.g., breast cancer, lung cancer, and ovarian cancer, where lipid metabolism was hyperactive, while in the liver cancer it was relatively hypoactive^26,27,59–62^. Moreover, hypoactive lipid metabolism was associated with a worse prognosis and advanced tumor stage, indicating that lipid metabolism played an anti-cancer role. This conclusion is confusing, because it is a prevalent opinion that lipid metabolism is pro-cancerous and is able to improve cancer progression. However, our finding was confirmed by other studies^47,48^. It is widely accepted that liver cells are the most hypermetabolic somatic cells. The metabolic level of the liver consists of two parts. Hepatocytes not only participate in their own cellular metabolism, but they also process foreign nutrients. We speculate that carcinogenesis and progression of HCC cells are accompanied by a gradient loss of metabolic abilities and a gain of other biological abilities such as cell division and DNA replication. This conclusion was consistent with our findings that LML cluster HCC was dominant in biological processes such as cell cycle or cell division, while LMH and LMI cluster HCC was dominant with metabolic process featured by liver cells (i.e., low-density lipoprotein remodeling, and intestinal cholesterol absorption). This is an interesting and novel theory, which requires confirmation.

By elucidating m6A gene signatures and defining the m6Ascore, we could more precisely correlate the mRNA transcriptomics differences to modification patterns of 3 m6A regulators. Functional annotation of these signatures showed enrichment in metabolism (especially lipid metabolism), which further established that m6A modification patterns shaped the metabolic state of HCC. For quantitation, we defined a scoring system, i.e., the m6Ascore, to evaluate the influence of m6A modification patterns on HCC. The output of this scoring system correlated with mRNA methylation levels, m6A regulator expression levels, and metabolic states, supported by the observation that the C1 cluster had the lowest score and the C3 cluster had the highest score, as well as by the differences in the expression levels of m6A regulators between m6Ascores of low and high HCC. Integrated analyses also showed that the m6Ascore was an independent prognostic biomarker in HCC, and clinical traits (e.g., higher TNM stage and younger age) were significantly correlated with higher m6Ascores. Collectively, these results showed that the m6Ascore was a reliable and robust tool for evaluating m6A modification patterns in HCC.

We determined the value of the m6Ascore in predicting patient responses to sorafenib therapy. In the STORM cohort, patients with high m6Ascores had a stronger response to sorafenib treatment, when compared with those of patients with low m6Ascores. However, no significant difference was observed in sorafenib-treated patients in TCGA cohort, which might be due to limited data. We also observed that HCC with high m6Ascores also had a high PR/SD percentage, when compared with that of HCC with low m6Ascores. This result indicated that the m6A modification pattern might have affected drug resistance in HCC. Further research on these aspects, especially on the mechanism linking the m6A modification pattern and sorafenib response, is urgently needed.

This study had some limitations. First, although many studies have revealed a positive relationship between m6A methyltransferases and mRNA m6A methylation^63–67^, the data still require validation by experiments such as m6Aseq, LC-MS, or MeRIP seq. However, due to data restriction, such validation was not possible in this study. Second, data using the GSVA enrichment score of metabolic associated pathways to evaluate the metabolic state were suggestive, but not definitive. Some metabolic pathways, such as fatty acid synthesis and fatty acid oxidation, might have inverse effects on oncogenesis. Due to their high overlapping genes, their GSVA enrichment scores may be similar. Hence, studies evaluating the effects of m6A regulators on key metabolic enzymes are urgently needed. Finally, due to the limitation of treatment response data, the response rate to sorafenib treatment was not convincing enough, and required further validation.

## Conclusions

Our findings provided new insight into the roles of m6A regulators in regulating biological processes and influencing the metabolic heterogeneity of HCC. Moreover, our results also led to novel concepts for improving patient responses to sorafenib treatment and for increasing treatment precision.

## References

[1] Jasirwan COM, Fahira A, Siregar L, Loho I (2020). The alpha-fetoprotein serum is still reliable as a biomarker for the surveillance of hepatocellular carcinoma in indonesia. BMC Gastroenterol.

[2] Roderburg C, Ozdirik B, Wree A, Demir M, Tacke F (2020). Systemic treatment of hepatocellular carcinoma: from sorafenib to combination therapies. Hepat Oncol.

[3] Yan J, Zhou B, Li H, Guo L, Ye Q (2020). Recent advances of GOLM1 in hepatocellular carcinoma. Hepat Oncol.

[4] Yoon JS, Lee DH, Cho EJ, Song MK, Choi YH, Kim GB (2020). Risk of liver cirrhosis and hepatocellular carcinoma after fontan operation: a need for surveillance. Cancers (Basel).

[5] Zhang T, Zhang L, Xu Y, Lu X, Zhao H, Yang H (2020). Neoadjuvant therapy and immunotherapy strategies for hepatocellular carcinoma. Am J Cancer Res.

[6] Sun T, Wu R, Ming L (2019). The role of m6A RNA methylation in cancer. Biomed Pharmacother.

[7] Brocard M, Ruggieri A, Locker N (2017). m6A RNA methylation, a new hallmark in virus-host interactions. J Gen Virol.

[8] Cao G, Li HB, Yin Z, Flavell RA (2016). Recent advances in dynamic m6A RNA modification. Open Biol.

[9] Coker H, Wei G, Brockdorff N (2019). m6A modification of non-coding RNA and the control of mammalian gene expression. Biochim Biophys Acta Gene Regul Mech.

[10] Dominissini D, Moshitch-Moshkovitz S, Schwartz S, Salmon-Divon M, Ungar L, Osenberg S (2012). Topology of the human and mouse m6A RNA methylomes revealed by m6A-seq. Nature.

[11] Huang H, Weng H, Sun W, Qin X, Shi H, Wu H (2018). Recognition of RNA n(6)-methyladenosine by IGF2BP proteins enhances mRNA stability and translation. Nat Cell Biol.

[12] Huang H, Weng H, Chen J (2020). M(6)A modification in coding and non-coding RNAS: roles and therapeutic implications in cancer. Cancer Cell.

[13] Liu L, Lei X, Meng J, Wei Z (2020). WITMSG: large-scale prediction of human intronic m(6)A RNA methylation sites from sequence and genomic features. Curr Genomics.

[14] Liu L, Wu Y, Li Q, Liang J, He Q, Zhao L (2020). METTL3 promotes tumorigenesis and metastasis through BMI1 m(6)A methylation in oral squamous cell carcinoma. Mol Ther.

[15] Shan K, Zhou RM, Xiang J, Sun YN, Liu C, Lv MW (2020). FTO regulates ocular angiogenesis via m(6)A-YTHDF2-dependent mechanism. Exp Eye Res.

[16] Wang M, Yang Y, Yang J, Yang J, Han S (2020). Circ_KIAA1429 accelerates hepatocellular carcinoma advancement through the mechanism of m(6)A-YTHDF3-Zeb1. Life Sci.

[17] Yu X, Zhao H, Cao Z (2020). The m6A methyltransferase METTL3 aggravates the progression of nasopharyngeal carcinoma through inducing EMT by m6A-modified snail mRNA. Minerva Med.

[18] Zaccara S, Jaffrey SR (2020). A unified model for the function of YTHDF proteins in regulating m(6)A-modified mRNA. Cell.

[19] Zeng M, Dai X, Liang Z, Sun R, Huang S, Luo L (2020). Critical roles of mRNA m(6)A modification and YTHDC2 expression for meiotic initiation and progression in female germ cells. Gene.

[20] Zhang BY, Han L, Tang YF, Zhang GX, Fan XL, Zhang JJ (2020). METTL14 regulates m6A methylation-modified primary miR-19a to promote cardiovascular endothelial cell proliferation and invasion. Eur Rev Med Pharmacol Sci.

[21] Zhang X, Wang F, Wang Z, Yang X, Yu H, Si S (2020). ALKBH5 promotes the proliferation of renal cell carcinoma by regulating AURKB expression in an m(6)A-dependent manner. Ann Transl Med.

[22] Liu ZX, Li LM, Sun HL, Liu SM (2018). Link between m6A modification and cancers. Front Bioeng Biotechnol.

[23] Campos-Contreras ADR, Diaz-Munoz M, Vazquez-Cuevas FG (2020). Purinergic signaling in the hallmarks of cancer. Cells.

[24] Dai X, Yu L, Zhao X, Ostrikov KK (2020). Nanomaterials for oncotherapies targeting the hallmarks of cancer. Nanotechnology.

[25] Muriithi W, Macharia LW, Heming CP, Echevarria JL, Nyachieo A, Filho PN (2020). ABC transporters and the hallmarks of cancer: roles in cancer aggressiveness beyond multidrug resistance. Cancer Biol Med.

[26] Wagner N, Wagner KD (2020). PPAR beta/delta and the hallmarks of cancer. Cells.

[27] Xavier CPR, Caires HR, Barbosa MAG, Bergantim R, Guimaraes JE, Vasconcelos MH (2020). The role of extracellular vesicles in the hallmarks of cancer and drug resistance. Cells.

[28] Zhong Z, Yu J, Virshup DM, Madan B (2020). Wnts and the hallmarks of cancer. Cancer Metastasis Rev.

[29] Chen B, Li Y, Song R, Xue C, Xu F (2019). Functions of RNA N6-methyladenosine modification in cancer progression. Mol Biol Rep.

[30] Yang S, Wei J, Cui YH, Park G, Shah P, Deng Y (2019). M(6)A mRNA demethylase FTO regulates melanoma tumorigenicity and response to anti-PD-1 blockade. Nat Commun.

[31] Chen M, Wei L, Law CT, Tsang FH, Shen J, Cheng CL (2018). RNA N6-methyladenosine methyltransferase-like 3 promotes liver cancer progression through YTHDF2-dependent posttranscriptional silencing of SOCS2. Hepatology.

[32] Chen Y, Peng C, Chen J, Chen D, Yang B, He B (2019). WTAP facilitates progression of hepatocellular carcinoma via m6A-Hur-dependent epigenetic silencing of ETS1. Mol Cancer.

[33] Lan T, Li H, Zhang D, Xu L, Liu H, Hao X (2019). KIAA1429 contributes to liver cancer progression through N6-methyladenosine-dependent post-transcriptional modification of GATA3. Mol Cancer.

[34] Zhao X, Chen Y, Mao Q, Jiang X, Jiang W, Chen J (2018). Overexpression of YTHDF1 is associated with poor prognosis in patients with hepatocellular carcinoma. Cancer Biomark.

[35] Zhou Y, Yin Z, Hou B, Yu M, Chen R, Jin H (2019). Expression profiles and prognostic significance of RNA N6-methyladenosine-related genes in patients with hepatocellular carcinoma: evidence from independent datasets. Cancer Manag Res.

[36] Cantor JR, Sabatini DM (2012). Cancer cell metabolism: one hallmark, many faces. Cancer Discov.

[37] De Matteis S, Ragusa A, Marisi G, De Domenico S, Casadei Gardini A, Bonafe M (2018). Aberrant metabolism in hepatocellular carcinoma provides diagnostic and therapeutic opportunities. Oxid Med Cell Longev.

[38] Shen C, Xuan B, Yan T, Ma Y, Xu P, Tian X (2020). M(6)A-dependent glycolysis enhances colorectal cancer progression. Mol Cancer.

[39] Zhong X, Yu J, Frazier K, Weng X, Li Y, Cham CM (2018). Circadian clock regulation of hepatic lipid metabolism by modulation of m(6)A mRNA methylation. Cell Rep.

[40] Wu W, Feng J, Jiang D, Zhou X, Jiang Q, Cai M (2017). AMPK regulates lipid accumulation in skeletal muscle cells through FTO-dependent demethylation of N(6)-methyladenosine. Sci Rep.

[41] Cheng X, Li M, Rao X, Zhang W, Li X, Wang L (2019). KIAA1429 regulates the migration and invasion of hepatocellular carcinoma by altering m6A modification of ID2 mRNA. Onco Targets Ther.

[42] Li J, Zhu L, Shi Y, Liu J, Lin L, Chen X (2019). m6A demethylase FTO promotes hepatocellular carcinoma tumorigenesis via mediating PKM2 demethylation. Am J Transl Res.

[43] Chai RC, Wu F, Wang QX, Zhang S, Zhang KN, Liu YQ (2019). M(6)A RNA methylation regulators contribute to malignant progression and have clinical prognostic impact in gliomas. Aging (Albany NY).

[44] Wu L, Wu D, Ning J, Liu W, Zhang D (2019). Changes of N6-methyladenosine modulators promote breast cancer progression. BMC Cancer.

[45] Hanzelmann S, Castelo R, Guinney J (2013). GSVA: gene set variation analysis for microarray and RNA-seq data. BMC Bioinformatics.

[46] Cancer Genome Atlas Research Network (2017). Electronic address wbe, Cancer Genome Atlas Research N. Comprehensive and integrative genomic characterization of hepatocellular carcinoma. Cell.

[47] Bidkhori G, Benfeitas R, Klevstig M, Zhang C, Nielsen J, Uhlen M (2018). Metabolic network-based stratification of hepatocellular carcinoma reveals three distinct tumor subtypes. Proc Natl Acad Sci U S A.

[48] Peng X, Chen Z, Farshidfar F, Xu X, Lorenzi PL, Wang Y (2018). Molecular characterization and clinical relevance of metabolic expression subtypes in human cancers. Cell Rep.

[49] Bi Z, Liu Y, Zhao Y, Yao Y, Wu R, Liu Q (2019). A dynamic reversible RNA N(6) -methyladenosine modification: current status and perspectives. J Cell Physiol.

[50] Xie W, Ma LL, Xu YQ, Wang BH, Li SM (2019). METTL3 inhibits hepatic insulin sensitivity via N6-methyladenosine modification of Fasn mRNA and promoting fatty acid metabolism. Biochem Biophys Res Commun.

[51] Yadav PK, Rajvanshi PK, Rajasekharan R (2018). The role of yeast m(6)A methyltransferase in peroxisomal fatty acid oxidation. Curr Genet.

[52] Wang Y, Lu JH, Wu QN, Jin Y, Wang DS, Chen YX (2019). LncRNA linris stabilizes IGF2BP2 and promotes the aerobic glycolysis in colorectal cancer. Mol Cancer.

[53] Yang Y, Shen F, Huang W, Qin S, Huang JT, Sergi C (2019). Glucose is involved in the dynamic regulation of m6A in patients with type 2 diabetes. J Clin Endocrinol Metab.

[54] Zhong H, Tang HF, Kai Y (2020). N6-methyladenine RNA modification (m6A): an emerging regulator of metabolic diseases. Curr Drug Targets.

[55] Chen XY, Zhang J, Zhu JS (2019). The role of m(6)A RNA methylation in human cancer. Mol Cancer.

[56] Kwok CT, Marshall AD, Rasko JE, Wong JJ (2017). Genetic alterations of m(6)A regulators predict poorer survival in acute myeloid leukemia. J Hematol Oncol.

[57] Zhou J, Wang J, Hong B, Ma K, Xie H, Li L (2019). Gene signatures and prognostic values of m6A regulators in clear cell renal cell carcinoma – a retrospective study using TCGA database. Aging (Albany NY).

[58] Meng Y, Li S, Gu D, Xu K, Du M, Zhu L (2020). Genetic variants in m6A modification genes are associated with colorectal cancer risk. Carcinogenesis.

[59] Kawamura E, Habu D, Ohfuji S, Fukushima W, Enomoto M, Torii K (2008). Clinical role of FDG-PET for HCC: relationship of glucose metabolic indicator to Japan Integrated Staging (JIS) score. Hepatogastroenterology.

[60] Montella M, Crispo A, Giudice A (2011). HCC, diet and metabolic factors: diet and HCC. Hepat Mon.

[61] Vigano L, Conci S, Cescon M, Fava C, Capelli P, D’Errico A (2015). Liver resection for hepatocellular carcinoma in patients with metabolic syndrome: a multicenter matched analysis with HCV-related HCC. J Hepatol.

[62] Zhang X (2018). Nafld related-HCC: the relationship with metabolic disorders. Adv Exp Med Biol.

[63] Zhang C, Zhang M, Ge S, Huang W, Lin X, Gao J (2019). Reduced m6A modification predicts malignant phenotypes and augmented Wnt/PI3K-Akt signaling in gastric cancer. Cancer Med.

[64] Muller S, Glass M, Singh AK, Haase J, Bley N, Fuchs T (2019). IGF2BP1 promotes srf-dependent transcription in cancer in a m6A- and miRNA-dependent manner. Nucleic Acids Res.

[65] Taketo K, Konno M, Asai A, Koseki J, Toratani M, Satoh T (2018). The epitranscriptome m6A writer mettl3 promotes chemo- and radioresistance in pancreatic cancer cells. Int J Oncol.

[66] Xia T, Wu X, Cao M, Zhang P, Shi G, Zhang J (2019). The RNA m6A methyltransferase METTL3 promotes pancreatic cancer cell proliferation and invasion. Pathol Res Pract.

[67] He L, Li H, Wu A, Peng Y, Shu G, Yin G (2019). Functions of N6- methyladenosine and its role in cancer. Mol Cancer.

