# Supplementary material for: The m6A methylation landscape stratifies hepatocellular carcinoma into 3 subtypes with distinct metabolic characteristics
Source: Cancer Biol Med. 2020 Dec 15;17(4):937–52. doi: 10.20892/j.issn.2095-3941.2020.0402 (PMC7721089; doi:10.20892/j.issn.2095-3941.2020.0402)
Supplement: Supplementary file 1 [file cbm-17-937-s001.pdf]

Supplementary materials

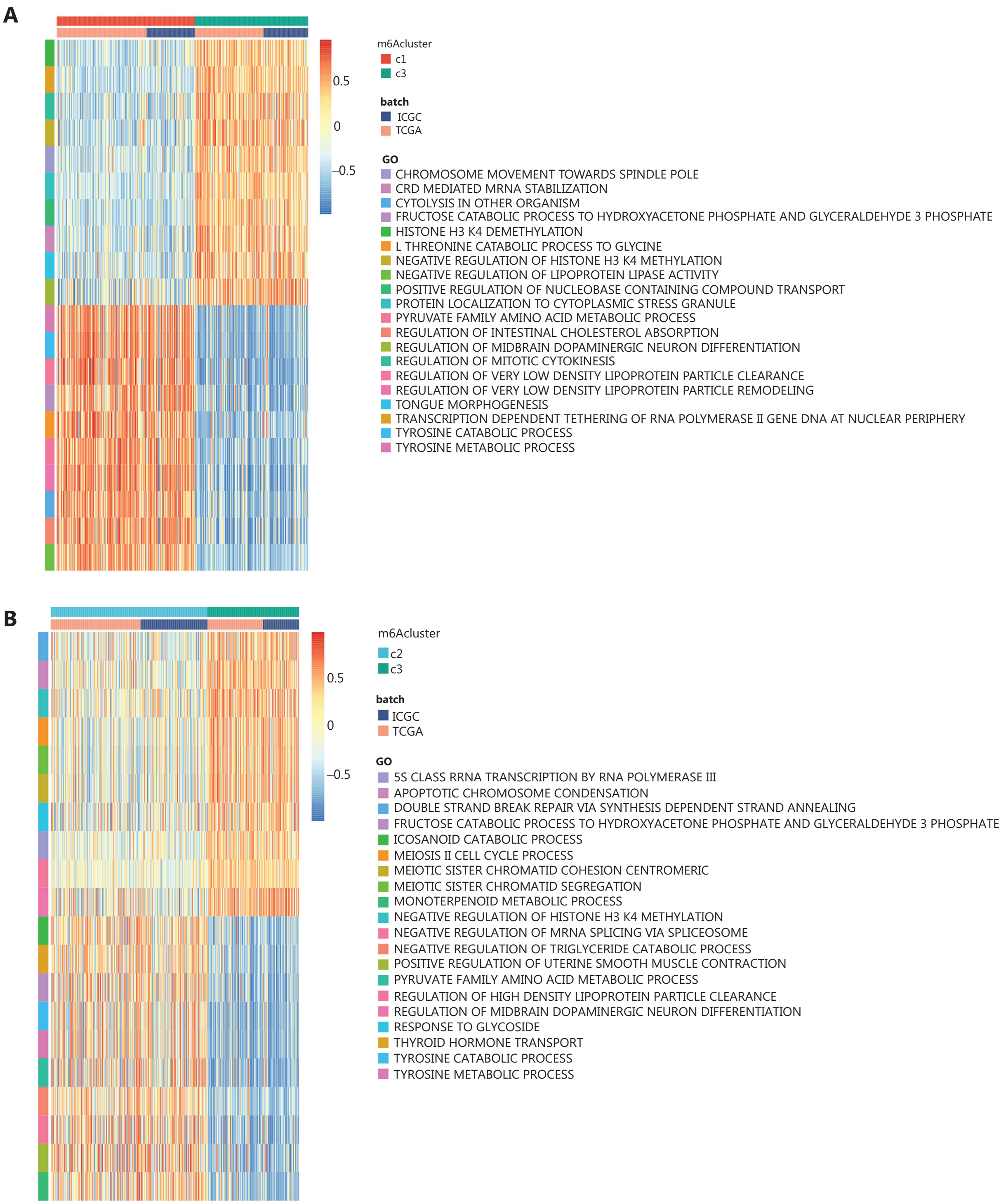

Figure S1 Biological characteristics of each m6A regulator modification pattern: Gene set variation analysis enrichment scores show the activation state of biological pathways between m6Acluster c1 and c3(A) or c2 and c3(B).

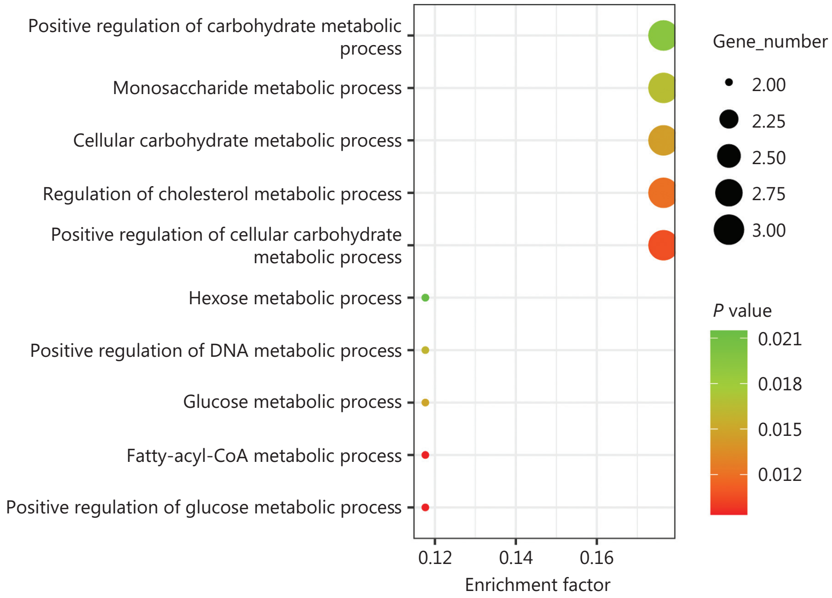

Figure S2 Metabolic associated terms of functional annotation to the 17 m6A target genes in hepatocellular carcinoma.

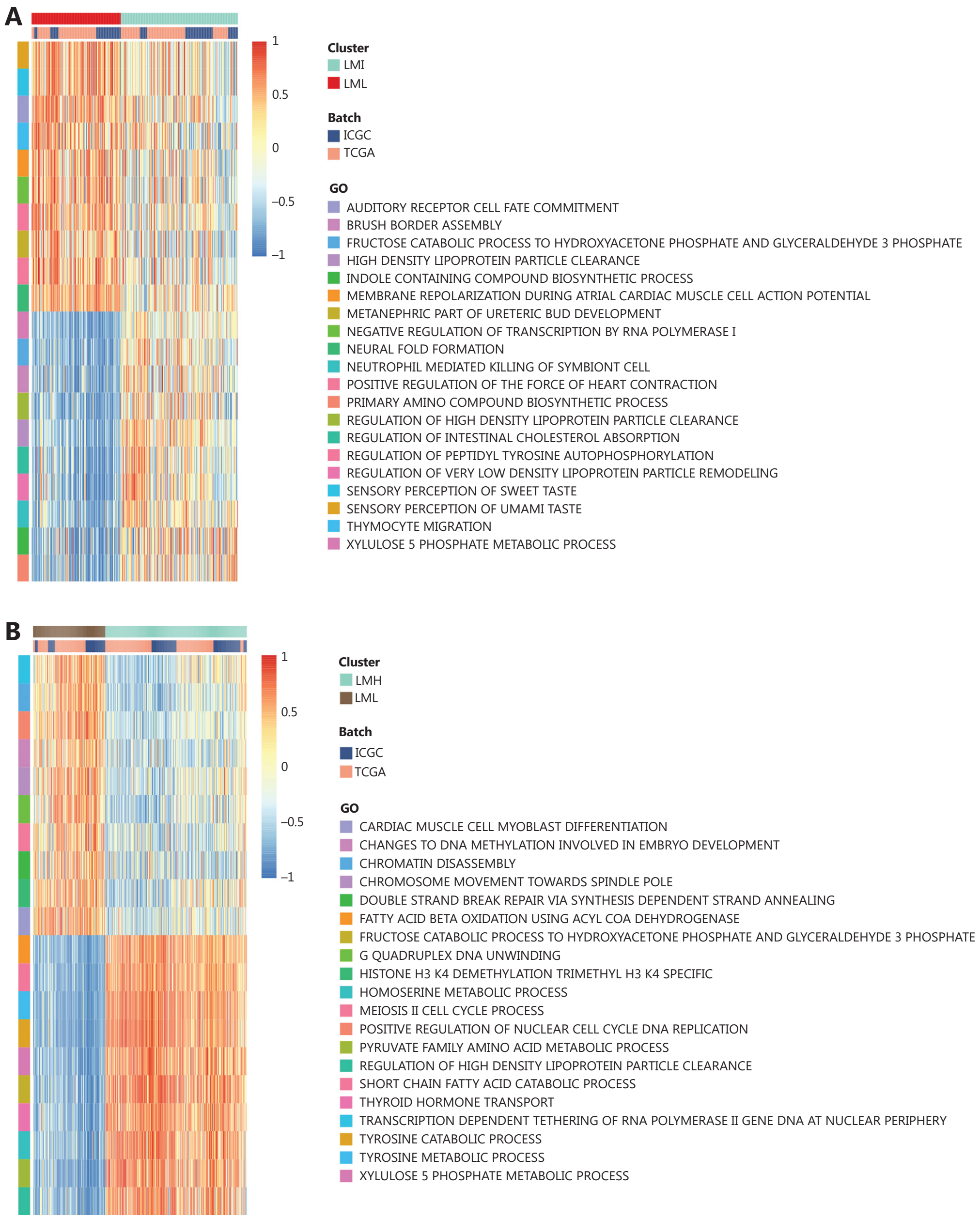

Figure S3 Biological characteristics of each metabolic subgroup; gene set variation analysis enrichment score shows the activation state of biological pathways between the metabolism low group (LML) and the metabolism medium group (A) or LML and the metabolism high group (B).

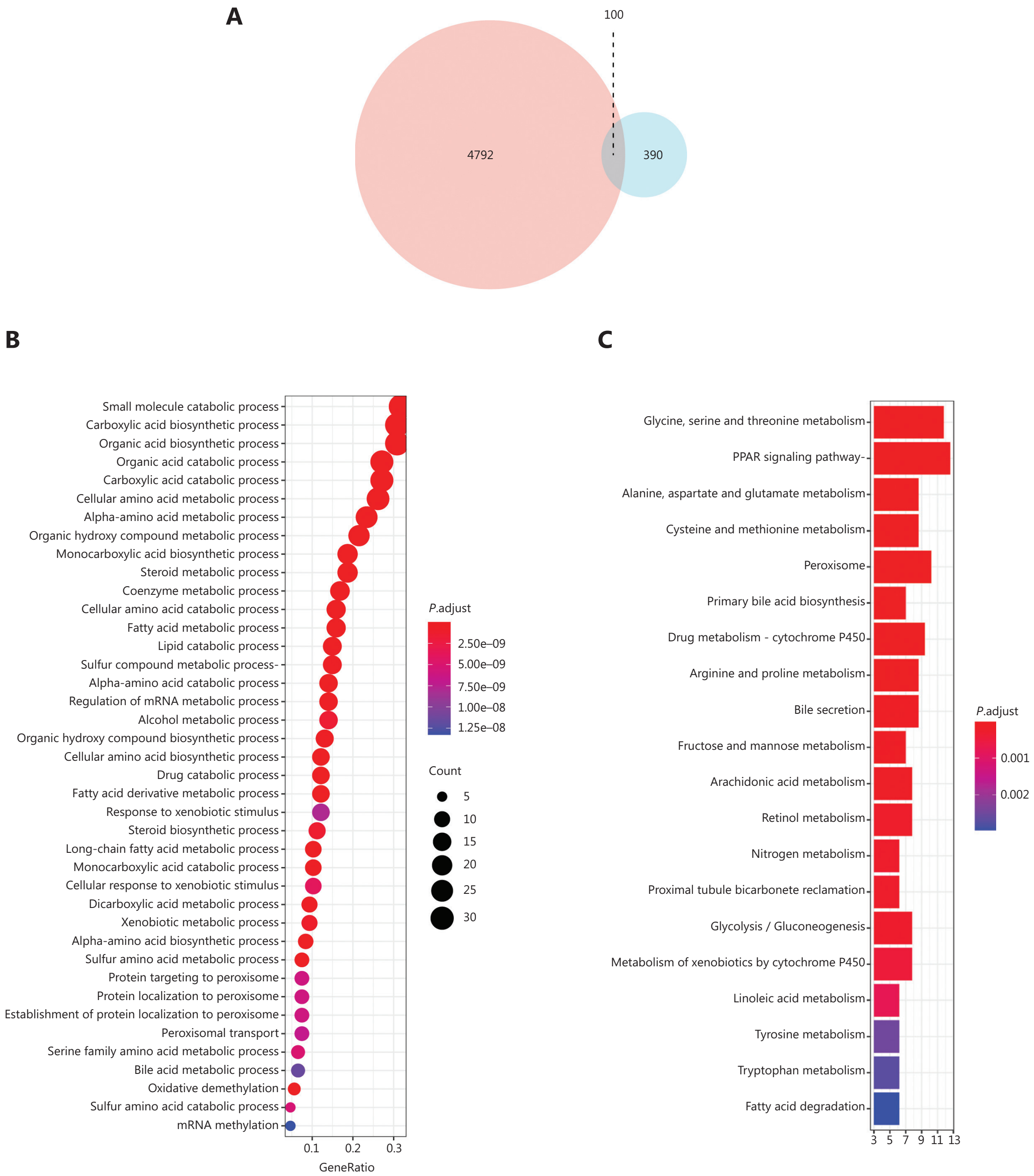

Figure S4 Functional annotation of the m6A gene signature (A) The Wenn plot shows the overlapped genes between genes in major metabolic pathways (pink) and m6A gene signatures (blue). (B, C): functional annotation for the m6A gene signature using the Gene Ontology (B) or Kyoto Encyclopedia of Genes and Genomes (C) databases.

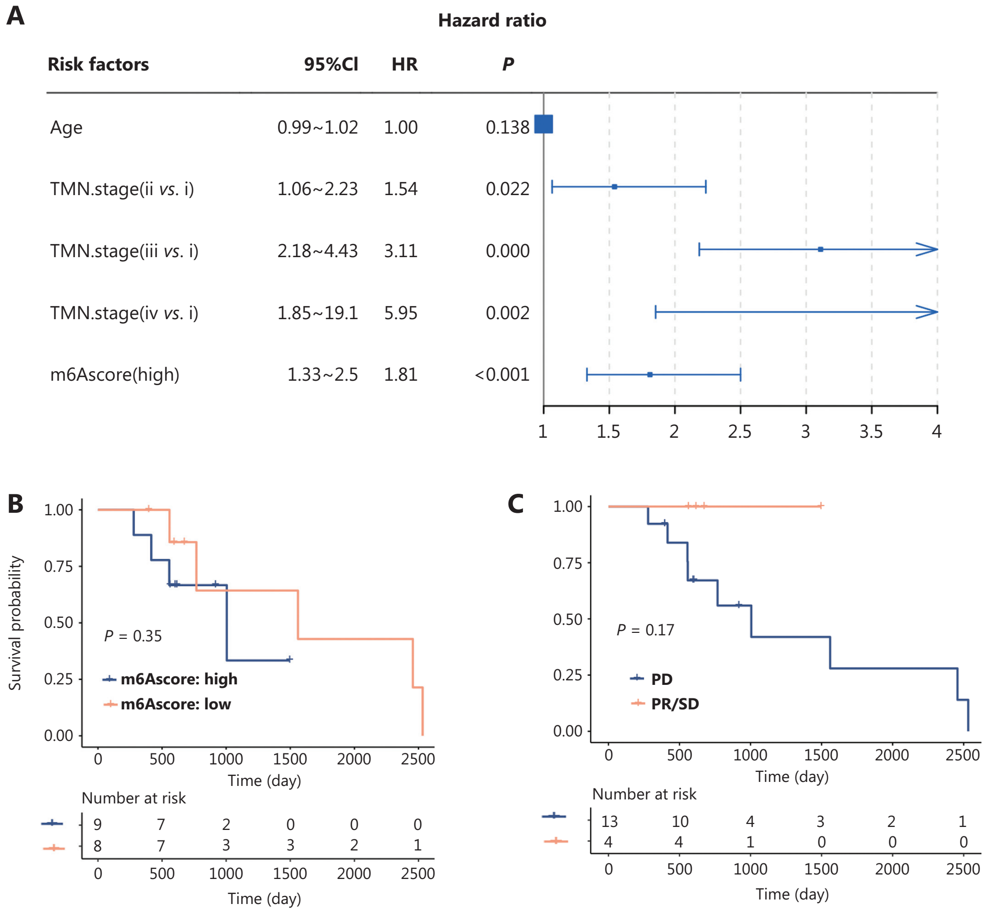

Figure S5 Multivariate COX regression analysis evaluated the prognosis value of the m6Ascore. A: multivariate COX regression analysis of the factors included in the nomogram. (B, C): Kaplan-Meier curve of the sorafenib-treated patients in The Cancer Genome Atlas cohort, stratified by response (B) or m6Ascore (C).

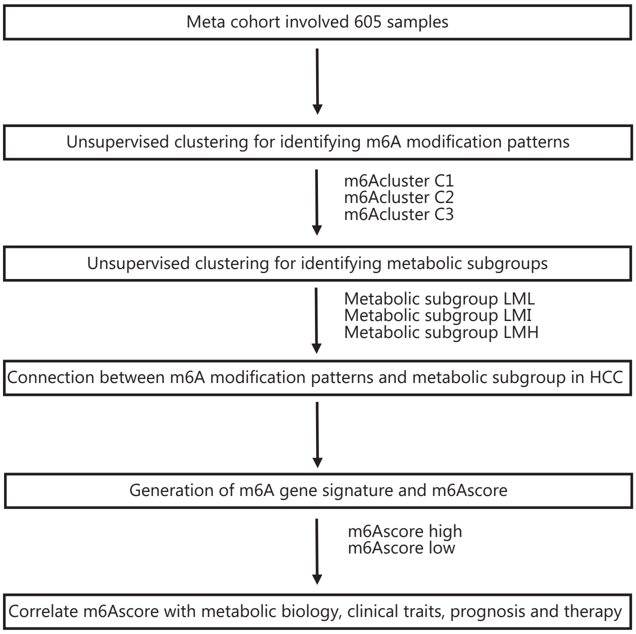

Flowchart A flowchart showing the design of this study.

Supplementary Table S1 PubMed-mined genes modulated directly by m6A regulators

m6A genes	cell_line	target	PMID
METTL3	Huh7	HCV RNA	27773535
METTL14	Huh7	HCV RNA	27773535
FTO	Huh7	HCV RNA	27773535
FTO	Huh7	IFITM1	27773535
YTHDF1	Huh7	HCV RNA	27773535
YTHDF2	Huh7	HCV RNA	27773535
YTHDF3	Huh7	HCV RNA	27773535
YTHDF2	Huh7	SOCS2	29171881
YTHDF2	HepG2	HSP90AA1	29902206
YTHDF2	HepG2	HSPD1	29902206
YTHDF2	HepG2	HSPB1	29902206
METTL3	HepG2	HSP70	29902206
METTL3	HepG2	HSP27	29902206
METTL3	HepG2	HSP60	29902206
IGF2BP1	HepG2	MYC	29476152
IGF2BP1	HepG2	FSCN1	29476152
IGF2BP1	HepG2	TK1	29476152
IGF2BP1	HepG2	MARCKSL1	29476152
METTL14	HepG2	MYC	29476152
METTL14	HepG2	FSCN1	29476152
METTL14	HepG2	TK1	29476152
METTL14	HepG2	MARCKSL1	29476152
IGF2BP2	HepG2	MYC	29476152
IGF2BP2	HepG2	FSCN1	29476152
IGF2BP2	HepG2	TK1	29476152
IGF2BP2	HepG2	MARCKSL1	29476152
IGF2BP3	HepG2	MYC	29476152
IGF2BP3	HepG2	FSCN1	29476152
IGF2BP3	HepG2	TK1	29476152
IGF2BP3	HepG2	MARCKSL1	29476152
FTO	HepG2	FOXO1	30137347
FTO	HepG2	FASN	30137347
FTO	HepG2	G6PC	30137347
FTO	HepG2	DGAT2	30137347
METTL3	HepG2	MDM4	22575960
METTL3	HepG2	MDM2	22575960
METTL3	HepG2	FAS	22575960
METTL3	HepG2	BAX	22575960
METTL3	HepG2	SOCS2	29171881
ZCCHC4	HepG2	28SrRNA	30531910

Supplementary Table S2 Functional annotation of PubMed-mined genes by the GO database

	ID	Description	Generation	Bg Ratio	*P* value	*P*. adjust	Q value	Gene ID	Count
GO:0043281	GO:0043281	regulation of cysteine-type endopeptidase activity involved in apoptotic process	5/17	196/17913	8.29E-07	0.000843	0.000489	3329/4609/4193/355/581	5
GO:2000116	GO:2000116	regulation of cysteine-type endopeptidase activity	5/17	222/17913	1.53E-06	0.000843	0.000489	3329/4609/4193/355/581	5
GO:0071453	GO:0071453	cellular response to oxygen levels	5/17	237/17913	2.11E-06	0.000843	0.000489	4609/2308/4194/4193/355	5
GO:0043280	GO:0043280	positive regulation of cysteine-type endopeptidase activity involved in apoptotic process	4/17	118/17913	3.99E-06	0.001193	0.000692	3329/4609/355/581	4
GO:2001056	GO:2001056	positive regulation of cysteine-type endopeptidase activity	4/17	135/17913	6.80E-06	0.001337	0.000776	3329/4609/355/581	4
GO:0009409	GO:0009409	response to cold	3/17	43/17913	8.56E-06	0.001337	0.000776	3320/3329/2308	3
GO:0045862	GO:0045862	positive regulation of proteolysis	5/17	333/17913	1.11E-05	0.001337	0.000776	3329/4609/4193/355/581	5
GO:0006986	GO:0006986	response to unfolded protein	4/17	161/17913	1.37E-05	0.001337	0.000776	3320/3329/3315/581	4
GO:0010950	GO:0010950	positive regulation of endopeptidase activity	4/17	161/17913	1.37E-05	0.001337	0.000776	3329/4609/355/581	4
GO:0072132	GO:0072132	mesenchyme morphogenesis	3/17	51/17913	1.44E-05	0.001337	0.000776	4609/4194/4193	3
GO:0010332	GO:0010332	response to gamma radiation	3/17	55/17913	1.81E-05	0.001337	0.000776	4609/4193/581	3
GO:0052548	GO:0052548	regulation of endopeptidase activity	5/17	370/17913	1.85E-05	0.001337	0.000776	3329/4609/4193/355/581	5
GO:0035966	GO:0035966	response to topologically incorrect protein	4/17	175/17913	1.90E-05	0.001337	0.000776	3320/3329/3315/581	4
GO:0006977	GO:0006977	DNA damage response, signal transduction by p53 class mediator resulting in cell cycle arrest	3/17	56/17913	1.91E-05	0.001337	0.000776	4194/4193/581	3
GO:0072431	GO:0072431	signal transduction involved in mitotic G1 DNA damage checkpoint	3/17	57/17913	2.01E-05	0.001337	0.000776	4194/4193/581	3
GO:1902400	GO:1902400	intracellular signal transduction involved in G1 DNA damage checkpoint	3/17	57/17913	2.01E-05	0.001337	0.000776	4194/4193/581	3
GO:0010952	GO:0010952	positive regulation of peptidase activity	4/17	180/17913	2.12E-05	0.001337	0.000776	3329/4609/355/581	4
GO:0072413	GO:0072413	signal transduction involved in mitotic cell cycle checkpoint	3/17	59/17913	2.23E-05	0.001337	0.000776	4194/4193/581	3
GO:1902402	GO:1902402	signal transduction involved in mitotic DNA damage checkpoint	3/17	59/17913	2.23E-05	0.001337	0.000776	4194/4193/581	3
GO:1902403	GO:1902403	signal transduction involved in mitotic DNA integrity checkpoint	3/17	59/17913	2.23E-05	0.001337	0.000776	4194/4193/581	3
GO:0070482	GO:0070482	response to oxygen levels	5/17	389/17913	2.35E-05	0.001339	0.000777	4609/2308/4194/4193/355	5
GO:0052547	GO:0052547	regulation of peptidase activity	5/17	398/17913	2.62E-05	0.001357	0.000788	3329/4609/4193/355/581	5

Supplementary Table S2 Continued

	ID	Description	Generation	Bg Ratio	*P* value	*P*. adjust	Q value	Gene ID	Count
GO:0031571	GO:0031571	mitotic G1 DNA damage checkpoint	3/17	63/17913	2.72E-05	0.001357	0.000788	4194/4193/581	3
GO:0044819	GO:0044819	mitotic G1/S transition checkpoint	3/17	63/17913	2.72E-05	0.001357	0.000788	4194/4193/581	3
GO:0044783	GO:0044783	G1 DNA damage checkpoint	3/17	64/17913	2.85E-05	0.001366	0.000793	4194/4193/581	3
GO:0072401	GO:0072401	signal transduction involved in DNA integrity checkpoint	3/17	73/17913	4.24E-05	0.001851	0.001074	4194/4193/581	3
GO:0072422	GO:0072422	signal transduction involved in DNA damage checkpoint	3/17	73/17913	4.24E-05	0.001851	0.001074	4194/4193/581	3
GO:0072395	GO:0072395	signal transduction involved in cell cycle checkpoint	3/17	74/17913	4.41E-05	0.001851	0.001074	4194/4193/581	3
GO:0006919	GO:0006919	activation of cysteine-type endopeptidase activity involved in apoptotic process	3/17	75/17913	4.60E-05	0.001851	0.001074	3329/355/581	3
GO:2001269	GO:2001269	positive regulation of cysteine-type endopeptidase activity involved in apoptotic signaling pathway	2/17	11/17913	4.64E-05	0.001851	0.001074	355/581	2
GO:2001234	GO:2001234	negative regulation of apoptotic signaling pathway	4/17	223/17913	4.90E-05	0.00186	0.00108	3315/4193/355/581	4
GO:0034644	GO:0034644	cellular response to UV	3/17	77/17913	4.97E-05	0.00186	0.00108	4609/4193/581	3
GO:0007050	GO:0007050	cell cycle arrest	4/17	231/17913	5.62E-05	0.002039	0.001183	4609/4194/4193/581	4
GO:0071158	GO:0071158	positive regulation of cell cycle arrest	3/17	82/17913	6.00E-05	0.002113	0.001227	4194/4193/581	3
GO:0006094	GO:0006094	gluconeogenesis	3/17	86/17913	6.92E-05	0.002301	0.001336	2308/2538/84649	3
GO:0044773	GO:0044773	mitotic DNA damage checkpoint	3/17	86/17913	6.92E-05	0.002301	0.001336	4194/4193/581	3
GO:0036295	GO:0036295	cellular response to increased oxygen levels	2/17	14/17913	7.66E-05	0.002415	0.001402	2308/355	2
GO:0019319	GO:0019319	hexose biosynthetic process	3/17	89/17913	7.67E-05	0.002415	0.001402	2308/2538/84649	3
GO:0044774	GO:0044774	mitotic DNA integrity checkpoint	3/17	90/17913	7.93E-05	0.002433	0.001412	4194/4193/581	3
GO:0046364	GO:0046364	monosaccharide biosynthetic process	3/17	93/17913	8.74E-05	0.002615	0.001518	2308/2538/84649	3
GO:0045722	GO:0045722	positive regulation of gluconeogenesis	2/17	16/17913	0.000101	0.002824	0.001639	2308/84649	2
GO:0051131	GO:0051131	chaperone-mediated protein complex assembly	2/17	16/17913	0.000101	0.002824	0.001639	3320/3329	2
GO:0000082	GO:0000082	G1/S transition of mitotic cell cycle	4/17	269/17913	0.000101	0.002824	0.001639	4609/4194/4193/581	4
GO:0031647	GO:0031647	regulation of protein stability	4/17	272/17913	0.000106	0.002881	0.001672	3320/3329/4194/4193	4
GO:2001267	GO:2001267	regulation of cysteine-type endopeptidase activity involved in apoptotic signaling pathway	2/17	17/17913	0.000114	0.003041	0.001765	355/581	2
GO:0042026	GO:0042026	protein refolding	2/17	18/17913	0.000129	0.003284	0.001906	3320/3329	2

	ID	Description	Generation	Bg Ratio	*P* value	*P*. adjust	Q value	Gene ID	Count
GO:0030330	GO:0030330	DNA damage response, signal transduction by p53 class mediator	3/17	106/17913	0.000129	0.003284	0.001906	4194/4193/581	3
GO:0044843	GO:0044843	cell cycle G1/S phase transition	4/17	288/17913	0.000132	0.003292	0.001911	4609/4194/4193/581	4
GO:0071156	GO:0071156	regulation of cell cycle arrest	3/17	108/17913	0.000136	0.003329	0.001932	4194/4193/581	3
GO:0071482	GO:0071482	cellular response to light stimulus	3/17	113/17913	0.000156	0.003731	0.002165	4609/4193/581	3
GO:0071214	GO:0071214	cellular response to abiotic stimulus	4/17	310/17913	0.000175	0.003905	0.002266	4609/4193/355/581	4
GO:0104004	GO:0104004	cellular response to environmental stimulus	4/17	310/17913	0.000175	0.003905	0.002266	4609/4193/355/581	4
GO:0003283	GO:0003283	atrial septum development	2/17	21/17913	0.000176	0.003905	0.002266	4194/4193	2
GO:0055093	GO:0055093	response to hyperoxia	2/17	21/17913	0.000176	0.003905	0.002266	2308/355	2
GO:2000134	GO:2000134	negative regulation of G1/S transition of mitotic cell cycle	3/17	124/17913	0.000205	0.004463	0.002591	4194/4193/581	3
GO:0071157	GO:0071157	negative regulation of cell cycle arrest	2/17	23/17913	0.000212	0.004531	0.00263	4194/4193	2
GO:0003181	GO:0003181	atrioventricular valve morphogenesis	2/17	24/17913	0.000231	0.004853	0.002817	4194/4193	2
GO:1902807	GO:1902807	negative regulation of cell cycle G1/S phase transition	3/17	130/17913	0.000236	0.004866	0.002824	4194/4193/581	3
GO:0042770	GO:0042770	signal transduction in response to DNA damage	3/17	131/17913	0.000241	0.004892	0.002839	4194/4193/581	3
GO:0000077	GO:0000077	DNA damage checkpoint	3/17	133/17913	0.000252	0.004953	0.002874	4194/4193/581	3
GO:0031331	GO:0031331	positive regulation of cellular catabolic process	4/17	341/17913	0.000252	0.004953	0.002874	3320/2308/4193/581	4
GO:0003171	GO:0003171	atrioventricular valve development	2/17	26/17913	0.000272	0.005227	0.003034	4194/4193	2
GO:0009411	GO:0009411	response to UV	3/17	137/17913	0.000275	0.005227	0.003034	4609/4193/581	3
GO:0030879	GO:0030879	mammary gland development	3/17	138/17913	0.000281	0.005257	0.003051	8835/2194/581	3
GO:0042176	GO:0042176	regulation of protein catabolic process	4/17	354/17913	0.000291	0.005361	0.003111	3320/2308/4194/4193	4
GO:0031570	GO:0031570	DNA integrity checkpoint	3/17	141/17913	0.000299	0.00543	0.003152	4194/4193/581	3
GO:0036296	GO:0036296	response to increased oxygen levels	2/17	28/17913	0.000316	0.005607	0.003254	2308/355	2
GO:0010212	GO:0010212	response to ionizing radiation	3/17	144/17913	0.000319	0.005607	0.003254	4609/4193/581	3
GO:0007093	GO:0007093	mitotic cell cycle checkpoint	3/17	147/17913	0.000338	0.00587	0.003407	4194/4193/581	3
GO:0009896	GO:0009896	positive regulation of catabolic process	4/17	392/17913	0.000429	0.007332	0.004256	3320/2308/4193/581	4
GO:0048147	GO:0048147	negative regulation of fibroblast proliferation	2/17	33/17913	0.00044	0.007417	0.004305	4609/581	2
GO:2001233	GO:2001233	regulation of apoptotic signaling pathway	4/17	397/17913	0.00045	0.007479	0.004341	3315/4193/355/581	4
GO:2001242	GO:2001242	regulation of intrinsic apoptotic signaling pathway	3/17	163/17913	0.000458	0.007509	0.004358	3315/4193/581	3
GO:0003203	GO:0003203	endocardial cushion morphogenesis	2/17	35/17913	0.000495	0.007698	0.004468	4194/4193	2
GO:0003230	GO:0003230	cardiac atrium development	2/17	35/17913	0.000495	0.007698	0.004468	4194/4193	2

Supplementary Table S2 Continued

	ID	Description	Generation	Bg Ratio	*P* value	*P*. adjust	Q value	Gene ID	Count
GO:0030224	GO:0030224	monocyte differentiation	2/17	35/17913	0.000495	0.007698	0.004468	4609/2194	2
GO:1903131	GO:1903131	mononuclear cell differentiation	2/17	35/17913	0.000495	0.007698	0.004468	4609/2194	2
GO:0060249	GO:0060249	anatomical structure homeostasis	4/17	413/17913	0.000522	0.007881	0.004574	3320/3315/4609/581	4
GO:0051973	GO:0051973	positive regulation of telomerase activity	2/17	36/17913	0.000524	0.007881	0.004574	3320/4609	2
GO:0050821	GO:0050821	protein stabilization	3/17	171/17913	0.000527	0.007881	0.004574	3320/3329/4194	3
GO:0001933	GO:0001933	negative regulation of protein phosphorylation	4/17	416/17913	0.000537	0.007931	0.004603	3315/4609/2308/581	4
GO:0071478	GO:0071478	cellular response to radiation	3/17	173/17913	0.000545	0.007954	0.004617	4609/4193/581	3
GO:0006457	GO:0006457	protein folding	3/17	176/17913	0.000573	0.008263	0.004796	3320/3329/3315	3
GO:2000045	GO:2000045	regulation of G1/S transition of mitotic cell cycle	3/17	177/17913	0.000582	0.0083	0.004817	4194/4193/581	3
GO:1901990	GO:1901990	regulation of mitotic cell cycle phase transition	4/17	428/17913	0.000597	0.008413	0.004883	3320/4194/4193/581	4
GO:0010907	GO:0010907	positive regulation of glucose metabolic process	2/17	41/17913	0.00068	0.009358	0.005431	2308/84649	2
GO:0035337	GO:0035337	fatty-acyl-CoA metabolic process	2/17	41/17913	0.00068	0.009358	0.005431	2194/84649	2
GO:0045732	GO:0045732	positive regulation of protein catabolic process	3/17	192/17913	0.000738	0.009925	0.00576	3320/2308/4193	3
GO:0071897	GO:0071897	DNA biosynthetic process	3/17	192/17913	0.000738	0.009925	0.00576	3320/4609/7083	3
GO:0042326	GO:0042326	negative regulation of phosphorylation	4/17	455/17913	0.000752	0.009997	0.005802	3315/4609/2308/581	4
GO:0016051	GO:0016051	carbohydrate biosynthetic process	3/17	194/17913	0.00076	0.010003	0.005806	2308/2538/84649	3
GO:1902806	GO:1902806	regulation of cell cycle G1/S phase transition	3/17	195/17913	0.000772	0.010043	0.005829	4194/4193/581	3
GO:0000075	GO:0000075	cell cycle checkpoint	3/17	197/17913	0.000795	0.010143	0.005887	4194/4193/581	3
GO:1901987	GO:1901987	regulation of cell cycle phase transition	4/17	464/17913	0.000809	0.010143	0.005887	3320/4194/4193/581	4
GO:0001974	GO:0001974	blood vessel remodeling	2/17	45/17913	0.000819	0.010143	0.005887	4193/581	2
GO:0003197	GO:0003197	endocardial cushion development	2/17	45/17913	0.000819	0.010143	0.005887	4194/4193	2
GO:0031667	GO:0031667	response to nutrient levels	4/17	466/17913	0.000822	0.010143	0.005887	2308/2538/4193/355	4
GO:0006111	GO:0006111	regulation of gluconeogenesis	2/17	46/17913	0.000856	0.010457	0.006069	2308/84649	2
GO:0006006	GO:0006006	glucose metabolic process	3/17	204/17913	0.00088	0.010636	0.006173	2308/2538/84649	3
GO:0051972	GO:0051972	regulation of telomerase activity	2/17	48/17913	0.000932	0.011156	0.006475	3320/4609	2
GO:0071456	GO:0071456	cellular response to hypoxia	3/17	210/17913	0.000957	0.011338	0.00658	4609/4194/4193	3
GO:0097300	GO:0097300	programmed necrotic cell death	2/17	49/17913	0.000971	0.011396	0.006614	355/581	2
GO:0032873	GO:0032873	negative regulation of stress-activated MAPK cascade	2/17	50/17913	0.001011	0.011636	0.006754	4609/2308	2

	ID	Description	Generation	Bg Ratio	*P* value	*P*. adjust	Q value	Gene ID	Count
GO:0070303	GO:0070303	negative regulation of stress-activated protein kinase signaling cascade	2/17	50/17913	0.001011	0.011636	0.006754	4609/2308	2
GO:0009991	GO:0009991	response to extracellular stimulus	4/17	498/17913	0.001053	0.011907	0.006911	2308/2538/4193/355	4
GO:0009266	GO:0009266	response to temperature stimulus	3/17	219/17913	0.00108	0.011907	0.006911	3320/3329/2308	3
GO:0003179	GO:0003179	heart valve morphogenesis	2/17	52/17913	0.001093	0.011907	0.006911	4194/4193	2
GO:0031669	GO:0031669	cellular response to nutrient levels	3/17	220/17913	0.001094	0.011907	0.006911	2308/4193/355	3
GO:0032872	GO:0032872	regulation of stress-activated MAPK cascade	3/17	220/17913	0.001094	0.011907	0.006911	4609/2308/355	3
GO:0036294	GO:0036294	cellular response to decreased oxygen levels	3/17	220/17913	0.001094	0.011907	0.006911	4609/4194/4193	3
GO:0051054	GO:0051054	positive regulation of DNA metabolic process	3/17	222/17913	0.001123	0.012004	0.006967	3320/4609/581	3
GO:0070302	GO:0070302	regulation of stress-activated protein kinase signaling cascade	3/17	222/17913	0.001123	0.012004	0.006967	4609/2308/355	3
GO:1901991	GO:1901991	negative regulation of mitotic cell cycle phase transition	3/17	235/17913	0.001323	0.014016	0.008135	4194/4193/581	3
GO:0019318	GO:0019318	hexose metabolic process	3/17	239/17913	0.001389	0.014456	0.00839	2308/2538/84649	3
GO:0031334	GO:0031334	positive regulation of protein complex assembly	3/17	239/17913	0.001389	0.014456	0.00839	3320/6624/581	3
GO:0010676	GO:0010676	positive regulation of cellular carbohydrate metabolic process	2/17	60/17913	0.001453	0.01499	0.0087	2308/84649	2
GO:0003170	GO:0003170	heart valve development	2/17	61/17913	0.001501	0.015357	0.008913	4194/4193	2
GO:0070265	GO:0070265	necrotic cell death	2/17	62/17913	0.00155	0.015726	0.009127	355/581	2
GO:0031668	GO:0031668	cellular response to extracellular stimulus	3/17	251/17913	0.001598	0.015962	0.009264	2308/4193/355	3
GO:0090181	GO:0090181	regulation of cholesterol metabolic process	2/17	63/17913	0.0016	0.015962	0.009264	2194/84649	2
GO:1901988	GO:1901988	negative regulation of cell cycle phase transition	3/17	254/17913	0.001654	0.01636	0.009495	4194/4193/581	3
GO:0044262	GO:0044262	cellular carbohydrate metabolic process	3/17	256/17913	0.001691	0.016594	0.009631	2308/2538/84649	3
GO:0060485	GO:0060485	mesenchyme development	3/17	258/17913	0.001729	0.01683	0.009768	4609/4194/4193	3
GO:2000573	GO:2000573	positive regulation of DNA biosynthetic process	2/17	66/17913	0.001755	0.01693	0.009826	3320/4609	2
GO:0016579	GO:0016579	protein deubiquitination	3/17	260/17913	0.001768	0.01693	0.009826	4609/4194/4193	3
GO:0072331	GO:0072331	signal transduction by p53 class mediator	3/17	264/17913	0.001847	0.017545	0.010183	4194/4193/581	3
GO:0051403	GO:0051403	stress-activated MAPK cascade	3/17	271/17913	0.00199	0.018757	0.010886	4609/2308/355	3
GO:0042100	GO:0042100	B cell proliferation	2/17	71/17913	0.002027	0.018857	0.010945	3329/581	2
GO:0070646	GO:0070646	protein modification by small protein removal	3/17	273/17913	0.002032	0.018857	0.010945	4609/4194/4193	3
GO:0005996	GO:0005996	monosaccharide metabolic process	3/17	277/17913	0.002118	0.019355	0.011234	2308/2538/84649	3

Supplementary Table S2 Continued

	ID	Description	Generation	Bg Ratio	*P* value	*P*. adjust	Q value	Gene ID	Count
GO:0097193	GO:0097193	intrinsic apoptotic signaling pathway	3/17	277/17913	0.002118	0.019355	0.011234	3315/4193/581	3
GO:0031098	GO:0031098	stress-activated protein kinase signaling cascade	3/17	278/17913	0.00214	0.019406	0.011264	4609/2308/355	3
GO:0090068	GO:0090068	positive regulation of cell cycle process	3/17	283/17913	0.002251	0.020175	0.01171	4194/4193/581	3
GO:0072332	GO:0072332	intrinsic apoptotic signaling pathway by p53 class mediator	2/17	75/17913	0.002259	0.020175	0.01171	4193/581	2
GO:0003281	GO:0003281	ventricular septum development	2/17	76/17913	0.002318	0.020556	0.01193	4194/4193	2
GO:0045913	GO:0045913	positive regulation of carbohydrate metabolic process	2/17	78/17913	0.00244	0.021476	0.012465	2308/84649	2
GO:0034599	GO:0034599	cellular response to oxidative stress	3/17	296/17913	0.002558	0.022238	0.012907	3315/2308/4193	3
GO:0110110	GO:0110110	positive regulation of animal organ morphogenesis	2/17	80/17913	0.002565	0.022238	0.012907	4609/581	2
GO:0071375	GO:0071375	cellular response to peptide hormone stimulus	3/17	297/17913	0.002582	0.022238	0.012907	8835/2308/4193	3
GO:0006112	GO:0006112	energy reserve metabolic process	2/17	83/17913	0.002757	0.023408	0.013586	4609/2538	2
GO:0008625	GO:0008625	extrinsic apoptotic signaling pathway *via* death domain receptors	2/17	83/17913	0.002757	0.023408	0.013586	355/581	2
GO:0009416	GO:0009416	response to light stimulus	3/17	307/17913	0.002836	0.023909	0.013877	4609/4193/581	3
GO:0045930	GO:0045930	negative regulation of mitotic cell cycle	3/17	308/17913	0.002863	0.023961	0.013907	4194/4193/581	3
GO:0042475	GO:0042475	odontogenesis of dentin-containing tooth	2/17	85/17913	0.002889	0.024019	0.01394	2308/581	2
GO:0048145	GO:0048145	regulation of fibroblast proliferation	2/17	86/17913	0.002957	0.024407	0.014166	4609/581	2
GO:0048144	GO:0048144	fibroblast proliferation	2/17	87/17913	0.003024	0.024797	0.014392	4609/581	2
GO:0071496	GO:0071496	cellular response to external stimulus	3/17	320/17913	0.003189	0.025967	0.015071	2308/4193/355	3
GO:0008202	GO:0008202	steroid metabolic process	3/17	321/17913	0.003217	0.02602	0.015102	2194/2538/84649	3
GO:0042632	GO:0042632	cholesterol homeostasis	2/17	91/17913	0.003303	0.026537	0.015402	2538/84649	2
GO:0055092	GO:0055092	sterol homeostasis	2/17	92/17913	0.003375	0.026793	0.015551	2538/84649	2
GO:0046677	GO:0046677	response to antibiotic	3/17	328/17913	0.003419	0.026793	0.015551	3320/2308/4193	3
GO:0006637	GO:0006637	acyl-CoA metabolic process	2/17	93/17913	0.003447	0.026793	0.015551	2194/84649	2
GO:0035383	GO:0035383	thioester metabolic process	2/17	93/17913	0.003447	0.026793	0.015551	2194/84649	2
GO:0048010	GO:0048010	vascular endothelial growth factor receptor signaling pathway	2/17	93/17913	0.003447	0.026793	0.015551	3320/3315	2
GO:0043255	GO:0043255	regulation of carbohydrate biosynthetic process	2/17	94/17913	0.00352	0.02701	0.015676	2308/84649	2
GO:2001243	GO:2001243	negative regulation of intrinsic apoptotic signaling pathway	2/17	94/17913	0.00352	0.02701	0.015676	3315/4193	2

	ID	Description	Generation	Bg Ratio	*P* value	*P*. adjust	Q value	Gene ID	Count
GO:0006641	GO:0006641	triglyceride metabolic process	2/17	95/17913	0.003594	0.0274	0.015903	2538/84649	2
GO:0070301	GO:0070301	cellular response to hydrogen peroxide	2/17	99/17913	0.003896	0.029427	0.017079	2308/4193	2
GO:0010948	GO:0010948	negative regulation of cell cycle process	3/17	344/17913	0.003909	0.029427	0.017079	4194/4193/581	3
GO:0001666	GO:0001666	response to hypoxia	3/17	354/17913	0.004236	0.031689	0.018392	4609/4194/4193	3
GO:2000278	GO:2000278	regulation of DNA biosynthetic process	2/17	106/17913	0.004452	0.033098	0.01921	3320/4609	2
GO:0035690	GO:0035690	cellular response to drug	3/17	362/17913	0.004509	0.033317	0.019337	4609/2308/4193	3
GO:1901653	GO:1901653	cellular response to peptide	3/17	364/17913	0.004579	0.033627	0.019517	8835/2308/4193	3
GO:0036293	GO:0036293	response to decreased oxygen levels	3/17	365/17913	0.004614	0.033679	0.019547	4609/4194/4193	3
GO:0045787	GO:0045787	positive regulation of cell cycle	3/17	373/17913	0.004902	0.035563	0.020641	4194/4193/581	3
GO:0003279	GO:0003279	cardiac septum development	2/17	113/17913	0.005042	0.035928	0.020852	4194/4193	2
GO:0006639	GO:0006639	acylglycerol metabolic process	2/17	113/17913	0.005042	0.035928	0.020852	2538/84649	2
GO:0010906	GO:0010906	regulation of glucose metabolic process	2/17	113/17913	0.005042	0.035928	0.020852	2308/84649	2
GO:0006638	GO:0006638	neutral lipid metabolic process	2/17	114/17913	0.00513	0.036333	0.021087	2538/84649	2
GO:0033135	GO:0033135	regulation of peptidyl-serine phosphorylation	2/17	119/17913	0.005576	0.039262	0.022788	3320/581	2
GO:0072655	GO:0072655	establishment of protein localization to mitochondrion	2/17	121/17913	0.005759	0.040316	0.023399	3320/581	2
GO:0007006	GO:0007006	mitochondrial membrane organization	2/17	122/17913	0.005852	0.040727	0.023638	3320/581	2
GO:0019216	GO:0019216	regulation of lipid metabolic process	3/17	402/17913	0.006037	0.04142	0.02404	8835/2194/84649	3
GO:0019218	GO:0019218	regulation of steroid metabolic process	2/17	124/17913	0.00604	0.04142	0.02404	2194/84649	2
GO:0051052	GO:0051052	regulation of DNA metabolic process	3/17	403/17913	0.006078	0.04142	0.02404	3320/4609/581	3
GO:0043434	GO:0043434	response to peptide hormone	3/17	404/17913	0.00612	0.04142	0.02404	8835/2308/4193	3
GO:0070585	GO:0070585	protein localization to mitochondrion	2/17	125/17913	0.006134	0.04142	0.02404	3320/581	2
GO:0030010	GO:0030010	establishment of cell polarity	2/17	126/17913	0.00623	0.04142	0.02404	3320/6624	2
GO:0042476	GO:0042476	odontogenesis	2/17	126/17913	0.00623	0.04142	0.02404	2308/581	2
GO:0033865	GO:0033865	nucleoside bisphosphate metabolic process	2/17	127/17913	0.006326	0.04142	0.02404	2194/84649	2
GO:0033875	GO:0033875	ribonucleoside bisphosphate metabolic process	2/17	127/17913	0.006326	0.04142	0.02404	2194/84649	2
GO:0034032	GO:0034032	purine nucleoside bisphosphate metabolic process	2/17	127/17913	0.006326	0.04142	0.02404	2194/84649	2
GO:0043254	GO:0043254	regulation of protein complex assembly	3/17	409/17913	0.006332	0.04142	0.02404	3320/6624/581	3
GO:1903364	GO:1903364	positive regulation of cellular protein catabolic process	2/17	129/17913	0.00652	0.042419	0.02462	3320/4193	2
GO:0003231	GO:0003231	cardiac ventricle development	2/17	131/17913	0.006718	0.043464	0.025227	4194/4193	2
GO:0055088	GO:0055088	lipid homeostasis	2/17	132/17913	0.006817	0.043871	0.025463	2538/84649	2

Supplementary Table S2 Continued

	ID	Description	Generation	Bg Ratio	*P* value	*P*. adjust	Q value	Gene ID	Count
GO:0048732	GO:0048732	gland development	3/17	422/17913	0.006904	0.044194	0.02565	8835/2194/581	3
GO:0009267	GO:0009267	cellular response to starvation	2/17	136/17913	0.007222	0.045983	0.026688	2308/355	2
GO:0010675	GO:0010675	regulation of cellular carbohydrate metabolic process	2/17	138/17913	0.007429	0.047048	0.027306	2308/84649	2
GO:0009314	GO:0009314	response to radiation	3/17	435/17913	0.007506	0.047289	0.027447	4609/4193/581	3
GO:0062012	GO:0062012	regulation of small molecule metabolic process	3/17	439/17913	0.007698	0.048241	0.027999	2308/2194/84649	3
GO:0006979	GO:0006979	response to oxidative stress	3/17	442/17913	0.007843	0.048896	0.028379	3315/2308/4193	3
GO:0008203	GO:0008203	cholesterol metabolic process	2/17	143/17913	0.007956	0.049092	0.028493	2194/84649	2
GO:0062013	GO:0062013	positive regulation of small molecule metabolic process	2/17	143/17913	0.007956	0.049092	0.028493	2308/84649	2
GO:0042542	GO:0042542	response to hydrogen peroxide	2/17	144/17913	0.008064	0.049501	0.02873	2308/4193	2
GO:0071236	GO:0071236	cellular response to antibiotic	2/17	147/17913	0.008391	0.050983	0.029591	2308/4193	2
GO:1902652	GO:1902652	secondary alcohol metabolic process	2/17	147/17913	0.008391	0.050983	0.029591	2194/84649	2
GO:0035264	GO:0035264	multicellular organism growth	2/17	149/17913	0.008612	0.052062	0.030217	8835/2538	2
GO:0000723	GO:0000723	telomere maintenance	2/17	154/17913	0.009176	0.053878	0.031271	3320/4609	2
GO:1901568	GO:1901568	fatty acid derivative metabolic process	2/17	154/17913	0.009176	0.053878	0.031271	2194/84649	2
GO:0016125	GO:0016125	sterol metabolic process	2/17	155/17913	0.009291	0.053878	0.031271	2194/84649	2
GO:0006914	GO:0006914	autophagy	3/17	471/17913	0.009334	0.053878	0.031271	3320/3315/2308	3
GO:0061919	GO:0061919	process utilizing autophagic mechanism	3/17	471/17913	0.009334	0.053878	0.031271	3320/3315/2308	3
GO:0048871	GO:0048871	multicellular organismal homeostasis	3/17	472/17913	0.009388	0.053878	0.031271	3315/2308/581	3
GO:0009162	GO:0009162	deoxyribonucleoside monophosphate metabolic process	1/17	10/17913	0.009452	0.053878	0.031271	7083	1
GO:0046322	GO:0046322	negative regulation of fatty acid oxidation	1/17	10/17913	0.009452	0.053878	0.031271	84649	1
GO:0060068	GO:0060068	vagina development	1/17	10/17913	0.009452	0.053878	0.031271	581	1
GO:0071803	GO:0071803	positive regulation of podosome assembly	1/17	10/17913	0.009452	0.053878	0.031271	6624	1
GO:0072203	GO:0072203	cell proliferation involved in metanephros development	1/17	10/17913	0.009452	0.053878	0.031271	4609	1
GO:0090557	GO:0090557	establishment of endothelial intestinal barrier	1/17	10/17913	0.009452	0.053878	0.031271	2194	1
GO:0002521	GO:0002521	leukocyte differentiation	3/17	485/17913	0.01011	0.056933	0.033044	4609/2194/581	3
GO:0002836	GO:0002836	positive regulation of response to tumor cell	1/17	11/17913	0.010393	0.056933	0.033044	3329	1
GO:0002839	GO:0002839	positive regulation of immune response to tumor cell	1/17	11/17913	0.010393	0.056933	0.033044	3329	1
GO:0008655	GO:0008655	pyrimidine-containing compound salvage	1/17	11/17913	0.010393	0.056933	0.033044	7083	1

	ID	Description	Generation	Bg Ratio	*P* value	*P*. adjust	Q value	Gene ID	Count
GO:0010623	GO:0010623	programmed cell death involved in cell development	1/17	11/17913	0.010393	0.056933	0.033044	581	1
GO:0040015	GO:0040015	negative regulation of multicellular organism growth	1/17	11/17913	0.010393	0.056933	0.033044	8835	1
GO:0043097	GO:0043097	pyrimidine nucleoside salvage	1/17	11/17913	0.010393	0.056933	0.033044	7083	1
GO:1905383	GO:1905383	protein localization to presynapse	1/17	11/17913	0.010393	0.056933	0.033044	3315	1
GO:1901652	GO:1901652	response to peptide	3/17	491/17913	0.010453	0.056933	0.033044	8835/2308/4193	3
GO:0034614	GO:0034614	cellular response to reactive oxygen species	2/17	165/17913	0.010474	0.056933	0.033044	2308/4193	2
GO:0022411	GO:0022411	cellular component disassembly	3/17	492/17913	0.010511	0.056933	0.033044	4609/6624/581	3
GO:0032200	GO:0032200	telomere organization	2/17	167/17913	0.010718	0.057518	0.033383	3320/4609	2
GO:0009636	GO:0009636	response to toxic substance	3/17	499/17913	0.010922	0.057518	0.033383	2308/4193/581	3
GO:0003205	GO:0003205	cardiac chamber development	2/17	170/17913	0.01109	0.057518	0.033383	4194/4193	2
GO:0048771	GO:0048771	tissue remodeling	2/17	170/17913	0.01109	0.057518	0.033383	4193/581	2
GO:0006662	GO:0006662	glycerol ether metabolic process	1/17	12/17913	0.011333	0.057518	0.033383	2194	1
GO:0009886	GO:0009886	post-embryonic animal morphogenesis	1/17	12/17913	0.011333	0.057518	0.033383	581	1
GO:0045472	GO:0045472	response to ether	1/17	12/17913	0.011333	0.057518	0.033383	4193	1
GO:0090151	GO:0090151	establishment of protein localization to mitochondrial membrane	1/17	12/17913	0.011333	0.057518	0.033383	3320	1
GO:0099640	GO:0099640	axo-dendritic protein transport	1/17	12/17913	0.011333	0.057518	0.033383	3315	1
GO:1900402	GO:1900402	regulation of carbohydrate metabolic process by regulation of transcription from RNA polymerase II promoter	1/17	12/17913	0.011333	0.057518	0.033383	2308	1
GO:1902337	GO:1902337	regulation of apoptotic process involved in morphogenesis	1/17	12/17913	0.011333	0.057518	0.033383	581	1
GO:1902510	GO:1902510	regulation of apoptotic DNA fragmentation	1/17	12/17913	0.011333	0.057518	0.033383	581	1
GO:1902947	GO:1902947	regulation of tau-protein kinase activity	1/17	12/17913	0.011333	0.057518	0.033383	3320	1
GO:1903894	GO:1903894	regulation of IRE1-mediated unfolded protein response	1/17	12/17913	0.011333	0.057518	0.033383	581	1
GO:0007163	GO:0007163	establishment or maintenance of cell polarity	2/17	172/17913	0.01134	0.057518	0.033383	3320/6624	2
GO:0042594	GO:0042594	response to starvation	2/17	174/17913	0.011593	0.058554	0.033984	2308/355	2
GO:2001235	GO:2001235	positive regulation of apoptotic signaling pathway	2/17	178/17913	0.012107	0.059711	0.034656	355/581	2
GO:0043409	GO:0043409	negative regulation of MAPK cascade	2/17	179/17913	0.012237	0.059711	0.034656	4609/2308	2
GO:1901796	GO:1901796	regulation of signal transduction by p53 class mediator	2/17	179/17913	0.012237	0.059711	0.034656	4194/4193	2

Supplementary Table S2 Continued

	ID	Description	Generation	Bg Ratio	*P* value	*P*. adjust	Q value	Gene ID	Count
GO:0014745	GO:0014745	negative regulation of muscle adaptation	1/17	13/17913	0.012271	0.059711	0.034656	2308	1
GO:0032530	GO:0032530	regulation of microvillus organization	1/17	13/17913	0.012271	0.059711	0.034656	6624	1
GO:0046415	GO:0046415	urate metabolic process	1/17	13/17913	0.012271	0.059711	0.034656	2538	1
GO:0048291	GO:0048291	isotype switching to IgG isotypes	1/17	13/17913	0.012271	0.059711	0.034656	3329	1
GO:0050746	GO:0050746	regulation of lipoprotein metabolic process	1/17	13/17913	0.012271	0.059711	0.034656	84649	1
GO:1900103	GO:1900103	positive regulation of endoplasmic reticulum unfolded protein response	1/17	13/17913	0.012271	0.059711	0.034656	581	1
GO:0002834	GO:0002834	regulation of response to tumor cell	1/17	14/17913	0.01321	0.06105	0.035433	3329	1
GO:0002837	GO:0002837	regulation of immune response to tumor cell	1/17	14/17913	0.01321	0.06105	0.035433	3329	1
GO:0010248	GO:0010248	establishment or maintenance of transmembrane electrochemical gradient	1/17	14/17913	0.01321	0.06105	0.035433	581	1
GO:0010867	GO:0010867	positive regulation of triglyceride biosynthetic process	1/17	14/17913	0.01321	0.06105	0.035433	84649	1
GO:0010917	GO:0010917	negative regulation of mitochondrial membrane potential	1/17	14/17913	0.01321	0.06105	0.035433	581	1
GO:0048569	GO:0048569	post-embryonic animal organ development	1/17	14/17913	0.01321	0.06105	0.035433	581	1
GO:0070166	GO:0070166	enamel mineralization	1/17	14/17913	0.01321	0.06105	0.035433	2308	1
GO:0071391	GO:0071391	cellular response to estrogen stimulus	1/17	14/17913	0.01321	0.06105	0.035433	4193	1
GO:0071801	GO:0071801	regulation of podosome assembly	1/17	14/17913	0.01321	0.06105	0.035433	6624	1
GO:0072216	GO:0072216	positive regulation of metanephros development	1/17	14/17913	0.01321	0.06105	0.035433	4609	1
GO:1901722	GO:1901722	regulation of cell proliferation involved in kidney development	1/17	14/17913	0.01321	0.06105	0.035433	4609	1
GO:1903624	GO:1903624	regulation of DNA catabolic process	1/17	14/17913	0.01321	0.06105	0.035433	581	1
GO:1904748	GO:1904748	regulation of apoptotic process involved in development	1/17	14/17913	0.01321	0.06105	0.035433	581	1
GO:0015671	GO:0015671	oxygen transport	1/17	15/17913	0.014147	0.062486	0.036267	4609	1
GO:0033599	GO:0033599	regulation of mammary gland epithelial cell proliferation	1/17	15/17913	0.014147	0.062486	0.036267	581	1
GO:0042535	GO:0042535	positive regulation of tumor necrosis factor biosynthetic process	1/17	15/17913	0.014147	0.062486	0.036267	3315	1
GO:0043518	GO:0043518	negative regulation of DNA damage response, signal transduction by p53 class mediator	1/17	15/17913	0.014147	0.062486	0.036267	4193	1
GO:0045837	GO:0045837	negative regulation of membrane potential	1/17	15/17913	0.014147	0.062486	0.036267	581	1

	ID	Description	Generation	Bg Ratio	*P* value	*P*. adjust	Q value	Gene ID	Count
GO:0055089	GO:0055089	fatty acid homeostasis	1/17	15/17913	0.014147	0.062486	0.036267	84649	1
GO:0072075	GO:0072075	metanephric mesenchyme development	1/17	15/17913	0.014147	0.062486	0.036267	4609	1
GO:0098840	GO:0098840	protein transport along microtubule	1/17	15/17913	0.014147	0.062486	0.036267	3315	1
GO:0099118	GO:0099118	microtubule-based protein transport	1/17	15/17913	0.014147	0.062486	0.036267	3315	1
GO:1900119	GO:1900119	positive regulation of execution phase of apoptosis	1/17	15/17913	0.014147	0.062486	0.036267	581	1
GO:1990000	GO:1990000	amyloid fibril formation	1/17	15/17913	0.014147	0.062486	0.036267	4193	1
GO:2001028	GO:2001028	positive regulation of endothelial cell chemotaxis	1/17	15/17913	0.014147	0.062486	0.036267	3315	1
GO:2000377	GO:2000377	regulation of reactive oxygen species metabolic process	2/17	194/17913	0.014261	0.062758	0.036425	3320/2308	2
GO:0046890	GO:0046890	regulation of lipid biosynthetic process	2/17	195/17913	0.014401	0.063141	0.036647	2194/84649	2
GO:0006109	GO:0006109	regulation of carbohydrate metabolic process	2/17	198/17913	0.014824	0.064023	0.037159	2308/84649	2
GO:0010225	GO:0010225	response to UV-C	1/17	16/17913	0.015083	0.064023	0.037159	4193	1
GO:0030033	GO:0030033	microvillus assembly	1/17	16/17913	0.015083	0.064023	0.037159	6624	1
GO:0043174	GO:0043174	nucleoside salvage	1/17	16/17913	0.015083	0.064023	0.037159	7083	1
GO:0046339	GO:0046339	diacylglycerol metabolic process	1/17	16/17913	0.015083	0.064023	0.037159	84649	1
GO:0048070	GO:0048070	regulation of developmental pigmentation	1/17	16/17913	0.015083	0.064023	0.037159	581	1
GO:0061684	GO:0061684	chaperone-mediated autophagy	1/17	16/17913	0.015083	0.064023	0.037159	3320	1
GO:0070242	GO:0070242	thymocyte apoptotic process	1/17	16/17913	0.015083	0.064023	0.037159	581	1
GO:0071732	GO:0071732	cellular response to nitric oxide	1/17	16/17913	0.015083	0.064023	0.037159	2308	1
GO:0002573	GO:0002573	myeloid leukocyte differentiation	2/17	201/17913	0.015252	0.064284	0.03731	4609/2194	2
GO:2001020	GO:2001020	regulation of response to DNA damage stimulus	2/17	201/17913	0.015252	0.064284	0.03731	4609/4193	2
GO:0002418	GO:0002418	immune response to tumor cell	1/17	17/17913	0.016019	0.066119	0.038375	3329	1
GO:0002902	GO:0002902	regulation of B cell apoptotic process	1/17	17/17913	0.016019	0.066119	0.038375	581	1
GO:0006071	GO:0006071	glycerol metabolic process	1/17	17/17913	0.016019	0.066119	0.038375	84649	1
GO:0010715	GO:0010715	regulation of extracellular matrix disassembly	1/17	17/17913	0.016019	0.066119	0.038375	6624	1
GO:0018904	GO:0018904	ether metabolic process	1/17	17/17913	0.016019	0.066119	0.038375	2194	1
GO:0030540	GO:0030540	female genitalia development	1/17	17/17913	0.016019	0.066119	0.038375	581	1
GO:0035089	GO:0035089	establishment of apical/basal cell polarity	1/17	18/17913	0.016953	0.068328	0.039657	6624	1
GO:0051782	GO:0051782	negative regulation of cell division	1/17	18/17913	0.016953	0.068328	0.039657	4609	1
GO:0060749	GO:0060749	mammary gland alveolus development	1/17	18/17913	0.016953	0.068328	0.039657	8835	1
GO:0061377	GO:0061377	mammary gland lobule development	1/17	18/17913	0.016953	0.068328	0.039657	8835	1

Supplementary Table S2 Continued

	ID	Description	Generation	Bg Ratio	*P* value	*P*. adjust	Q value	Gene ID	Count
GO:0070230	GO:0070230	positive regulation of lymphocyte apoptotic process	1/17	18/17913	0.016953	0.068328	0.039657	581	1
GO:0071800	GO:0071800	podosome assembly	1/17	18/17913	0.016953	0.068328	0.039657	6624	1
GO:1902176	GO:1902176	negative regulation of oxidative stress-induced intrinsic apoptotic signaling pathway	1/17	18/17913	0.016953	0.068328	0.039657	3315	1
GO:0097191	GO:0097191	extrinsic apoptotic signaling pathway	2/17	213/17913	0.017019	0.06836	0.039676	355/581	2
GO:0001894	GO:0001894	tissue homeostasis	2/17	214/17913	0.01717	0.068736	0.039894	3315/581	2
GO:0042113	GO:0042113	B cell activation	2/17	215/17913	0.017321	0.069112	0.040112	3329/581	2
GO:0010612	GO:0010612	regulation of cardiac muscle adaptation	1/17	19/17913	0.017887	0.069517	0.040347	2308	1
GO:0015669	GO:0015669	gas transport	1/17	19/17913	0.017887	0.069517	0.040347	4609	1
GO:0032026	GO:0032026	response to magnesium ion	1/17	19/17913	0.017887	0.069517	0.040347	4193	1
GO:0046597	GO:0046597	negative regulation of viral entry into host cell	1/17	19/17913	0.017887	0.069517	0.040347	8519	1
GO:0071731	GO:0071731	response to nitric oxide	1/17	19/17913	0.017887	0.069517	0.040347	2308	1
GO:0072074	GO:0072074	kidney mesenchyme development	1/17	19/17913	0.017887	0.069517	0.040347	4609	1
GO:1902170	GO:1902170	cellular response to reactive nitrogen species	1/17	19/17913	0.017887	0.069517	0.040347	2308	1
GO:1903242	GO:1903242	regulation of cardiac muscle hypertrophy in response to stress	1/17	19/17913	0.017887	0.069517	0.040347	2308	1
GO:0001649	GO:0001649	osteoblast differentiation	2/17	222/17913	0.018399	0.07079	0.041086	8519/2194	2
GO:0006839	GO:0006839	mitochondrial transport	2/17	224/17913	0.018712	0.07079	0.041086	3320/581	2
GO:0010592	GO:0010592	positive regulation of lamellipodium assembly	1/17	20/17913	0.01882	0.07079	0.041086	6624	1
GO:0019400	GO:0019400	alditol metabolic process	1/17	20/17913	0.01882	0.07079	0.041086	84649	1
GO:0032727	GO:0032727	positive regulation of interferon-alpha production	1/17	20/17913	0.01882	0.07079	0.041086	3329	1
GO:0032986	GO:0032986	protein-DNA complex disassembly	1/17	20/17913	0.01882	0.07079	0.041086	4609	1
GO:0034505	GO:0034505	tooth mineralization	1/17	20/17913	0.01882	0.07079	0.041086	2308	1
GO:0045655	GO:0045655	regulation of monocyte differentiation	1/17	20/17913	0.01882	0.07079	0.041086	4609	1
GO:0061162	GO:0061162	establishment of monopolar cell polarity	1/17	20/17913	0.01882	0.07079	0.041086	6624	1
GO:0070584	GO:0070584	mitochondrion morphogenesis	1/17	20/17913	0.01882	0.07079	0.041086	581	1
GO:0042593	GO:0042593	glucose homeostasis	2/17	227/17913	0.019185	0.07079	0.041086	2308/2538	2
GO:0033500	GO:0033500	carbohydrate homeostasis	2/17	228/17913	0.019344	0.07079	0.041086	2308/2538	2
GO:0000302	GO:0000302	response to reactive oxygen species	2/17	230/17913	0.019664	0.07079	0.041086	2308/4193	2

	ID	Description	Generation	Bg Ratio	*P* value	*P*. adjust	Q value	Gene ID	Count
GO:1903362	GO:1903362	regulation of cellular protein catabolic process	2/17	230/17913	0.019664	0.07079	0.041086	3320/4193	2
GO:0005980	GO:0005980	glycogen catabolic process	1/17	21/17913	0.019753	0.07079	0.041086	2538	1
GO:0008053	GO:0008053	mitochondrial fusion	1/17	21/17913	0.019753	0.07079	0.041086	581	1
GO:0021854	GO:0021854	hypothalamus development	1/17	21/17913	0.019753	0.07079	0.041086	581	1
GO:0032469	GO:0032469	endoplasmic reticulum calcium ion homeostasis	1/17	21/17913	0.019753	0.07079	0.041086	581	1
GO:0035455	GO:0035455	response to interferon-alpha	1/17	21/17913	0.019753	0.07079	0.041086	8519	1
GO:0046827	GO:0046827	positive regulation of protein export from nucleus	1/17	21/17913	0.019753	0.07079	0.041086	4193	1
GO:0060396	GO:0060396	growth hormone receptor signaling pathway	1/17	21/17913	0.019753	0.07079	0.041086	8835	1
GO:0061339	GO:0061339	establishment or maintenance of monopolar cell polarity	1/17	21/17913	0.019753	0.07079	0.041086	6624	1
GO:0072111	GO:0072111	cell proliferation involved in kidney development	1/17	21/17913	0.019753	0.07079	0.041086	4609	1
GO:0090208	GO:0090208	positive regulation of triglyceride metabolic process	1/17	21/17913	0.019753	0.07079	0.041086	84649	1
GO:0097186	GO:0097186	amelogenesis	1/17	21/17913	0.019753	0.07079	0.041086	2308	1
GO:1903055	GO:1903055	positive regulation of extracellular matrix organization	1/17	21/17913	0.019753	0.07079	0.041086	6624	1
GO:0018105	GO:0018105	peptidyl-serine phosphorylation	2/17	231/17913	0.019825	0.070837	0.041114	3320/581	2
GO:0006309	GO:0006309	apoptotic DNA fragmentation	1/17	22/17913	0.020684	0.07282	0.042264	581	1
GO:0009251	GO:0009251	glucan catabolic process	1/17	22/17913	0.020684	0.07282	0.042264	2538	1
GO:0010866	GO:0010866	regulation of triglyceride biosynthetic process	1/17	22/17913	0.020684	0.07282	0.042264	84649	1
GO:0043496	GO:0043496	regulation of protein homodimerization activity	1/17	22/17913	0.020684	0.07282	0.042264	581	1
GO:0071378	GO:0071378	cellular response to growth hormone stimulus	1/17	22/17913	0.020684	0.07282	0.042264	8835	1
GO:0097237	GO:0097237	cellular response to toxic substance	2/17	241/17913	0.021463	0.074346	0.043151	2308/4193	2
GO:0001783	GO:0001783	B cell apoptotic process	1/17	23/17913	0.021615	0.074346	0.043151	581	1
GO:0002347	GO:0002347	response to tumor cell	1/17	23/17913	0.021615	0.074346	0.043151	3329	1
GO:0006458	GO:0006458	"de novo" protein folding	1/17	23/17913	0.021615	0.074346	0.043151	3329	1
GO:0044247	GO:0044247	cellular polysaccharide catabolic process	1/17	23/17913	0.021615	0.074346	0.043151	2538	1
GO:0072215	GO:0072215	regulation of metanephros development	1/17	23/17913	0.021615	0.074346	0.043151	4609	1
GO:0097150	GO:0097150	neuronal stem cell population maintenance	1/17	23/17913	0.021615	0.074346	0.043151	2308	1

Supplementary Table S2 Continued

	ID	Description	Generation	Bg Ratio	*P* value	*P*. adjust	Q value	Gene ID	Count
GO:1902254	GO:1902254	negative regulation of intrinsic apoptotic signaling pathway by p53 class mediator	1/17	23/17913	0.021615	0.074346	0.043151	4193	1
GO:0046651	GO:0046651	lymphocyte proliferation	2/17	244/17913	0.021965	0.075336	0.043725	3329/581	2
GO:0032943	GO:0032943	mononuclear cell proliferation	2/17	246/17913	0.022303	0.076276	0.04427	3329/581	2
GO:0032528	GO:0032528	microvillus organization	1/17	24/17913	0.022544	0.076446	0.044369	6624	1
GO:0044346	GO:0044346	fibroblast apoptotic process	1/17	24/17913	0.022544	0.076446	0.044369	4609	1
GO:2001026	GO:2001026	regulation of endothelial cell chemotaxis	1/17	24/17913	0.022544	0.076446	0.044369	3315	1
GO:0003007	GO:0003007	heart morphogenesis	2/17	249/17913	0.022813	0.07714	0.044772	4194/4193	2
GO:2000027	GO:2000027	regulation of animal organ morphogenesis	2/17	250/17913	0.022984	0.0775	0.044981	4609/581	2
GO:0018209	GO:0018209	peptidyl-serine modification	2/17	252/17913	0.023329	0.077617	0.045049	3320/581	2
GO:0000272	GO:0000272	polysaccharide catabolic process	1/17	25/17913	0.023473	0.077617	0.045049	2538	1
GO:0032461	GO:0032461	positive regulation of protein oligomerization	1/17	25/17913	0.023473	0.077617	0.045049	581	1
GO:0035336	GO:0035336	long-chain fatty-acyl-CoA metabolic process	1/17	25/17913	0.023473	0.077617	0.045049	84649	1
GO:0046426	GO:0046426	negative regulation of JAK-STAT cascade	1/17	25/17913	0.023473	0.077617	0.045049	8835	1
GO:0060561	GO:0060561	apoptotic process involved in morphogenesis	1/17	25/17913	0.023473	0.077617	0.045049	581	1
GO:1900101	GO:1900101	regulation of endoplasmic reticulum unfolded protein response	1/17	25/17913	0.023473	0.077617	0.045049	581	1
GO:0002053	GO:0002053	positive regulation of mesenchymal cell proliferation	1/17	26/17913	0.024401	0.079586	0.046192	4609	1
GO:0032647	GO:0032647	regulation of interferon-alpha production	1/17	26/17913	0.024401	0.079586	0.046192	3329	1
GO:0033598	GO:0033598	mammary gland epithelial cell proliferation	1/17	26/17913	0.024401	0.079586	0.046192	581	1
GO:1902175	GO:1902175	regulation of oxidative stress-induced intrinsic apoptotic signaling pathway	1/17	26/17913	0.024401	0.079586	0.046192	3315	1
GO:1904754	GO:1904754	positive regulation of vascular associated smooth muscle cell migration	1/17	26/17913	0.024401	0.079586	0.046192	4193	1
GO:0001782	GO:0001782	B cell homeostasis	1/17	27/17913	0.025328	0.0815	0.047303	581	1
GO:0009651	GO:0009651	response to salt stress	1/17	27/17913	0.025328	0.0815	0.047303	581	1
GO:0033137	GO:0033137	negative regulation of peptidyl-serine phosphorylation	1/17	27/17913	0.025328	0.0815	0.047303	581	1
GO:0035456	GO:0035456	response to interferon-beta	1/17	27/17913	0.025328	0.0815	0.047303	8519	1
GO:0035902	GO:0035902	response to immobilization stress	1/17	27/17913	0.025328	0.0815	0.047303	4193	1
GO:0015980	GO:0015980	energy derivation by oxidation of organic compounds	2/17	264/17913	0.025438	0.081634	0.04738	4609/2538	2
GO:0000737	GO:0000737	DNA catabolic process, endonucleolytic	1/17	28/17913	0.026255	0.082485	0.047874	581	1

	ID	Description	Generation	Bg Ratio	*P* value	*P*. adjust	Q value	Gene ID	Count
GO:0032607	GO:0032607	interferon-alpha production	1/17	28/17913	0.026255	0.082485	0.047874	3329	1
GO:0042533	GO:0042533	tumor necrosis factor biosynthetic process	1/17	28/17913	0.026255	0.082485	0.047874	3315	1
GO:0042534	GO:0042534	regulation of tumor necrosis factor biosynthetic process	1/17	28/17913	0.026255	0.082485	0.047874	3315	1
GO:0044030	GO:0044030	regulation of DNA methylation	1/17	28/17913	0.026255	0.082485	0.047874	4609	1
GO:0090200	GO:0090200	positive regulation of release of cytochrome c from mitochondria	1/17	28/17913	0.026255	0.082485	0.047874	581	1
GO:1902745	GO:1902745	positive regulation of lamellipodium organization	1/17	28/17913	0.026255	0.082485	0.047874	6624	1
GO:2000108	GO:2000108	positive regulation of leukocyte apoptotic process	1/17	28/17913	0.026255	0.082485	0.047874	581	1
GO:0072593	GO:0072593	reactive oxygen species metabolic process	2/17	269/17913	0.02634	0.082535	0.047903	3320/2308	2
GO:0009410	GO:0009410	response to xenobiotic stimulus	2/17	270/17913	0.026522	0.082673	0.047983	2308/4193	2
GO:0070661	GO:0070661	leukocyte proliferation	2/17	270/17913	0.026522	0.082673	0.047983	3329/581	2
GO:0001844	GO:0001844	protein insertion into mitochondrial membrane involved in apoptotic signaling pathway	1/17	29/17913	0.02718	0.083423	0.048418	581	1
GO:0046134	GO:0046134	pyrimidine nucleoside biosynthetic process	1/17	29/17913	0.02718	0.083423	0.048418	7083	1
GO:0048873	GO:0048873	homeostasis of number of cells within a tissue	1/17	29/17913	0.02718	0.083423	0.048418	581	1
GO:0071480	GO:0071480	cellular response to gamma radiation	1/17	29/17913	0.02718	0.083423	0.048418	4193	1
GO:0071549	GO:0071549	cellular response to dexamethasone stimulus	1/17	29/17913	0.02718	0.083423	0.048418	2308	1
GO:1900117	GO:1900117	regulation of execution phase of apoptosis	1/17	29/17913	0.02718	0.083423	0.048418	581	1
GO:0001822	GO:0001822	kidney development	2/17	276/17913	0.027624	0.084566	0.049082	4609/581	2
GO:0038111	GO:0038111	interleukin-7-mediated signaling pathway	1/17	30/17913	0.028105	0.084954	0.049307	8835	1
GO:0046320	GO:0046320	regulation of fatty acid oxidation	1/17	30/17913	0.028105	0.084954	0.049307	84649	1
GO:0046596	GO:0046596	regulation of viral entry into host cell	1/17	30/17913	0.028105	0.084954	0.049307	8519	1
GO:0051491	GO:0051491	positive regulation of filopodium assembly	1/17	30/17913	0.028105	0.084954	0.049307	6624	1
GO:1904837	GO:1904837	beta-catenin-TCF complex assembly	1/17	30/17913	0.028105	0.084954	0.049307	4609	1
GO:0010591	GO:0010591	regulation of lamellipodium assembly	1/17	31/17913	0.029029	0.086222	0.050043	6624	1
GO:0030262	GO:0030262	apoptotic nuclear changes	1/17	31/17913	0.029029	0.086222	0.050043	581	1
GO:0035767	GO:0035767	endothelial cell chemotaxis	1/17	31/17913	0.029029	0.086222	0.050043	3315	1
GO:0042572	GO:0042572	retinol metabolic process	1/17	31/17913	0.029029	0.086222	0.050043	84649	1
GO:0051204	GO:0051204	protein insertion into mitochondrial membrane	1/17	31/17913	0.029029	0.086222	0.050043	581	1

Supplementary Table S2 Continued

	ID	Description	Generation	Bg Ratio	*P* value	*P*. adjust	Q value	Gene ID	Count
GO:1902253	GO:1902253	regulation of intrinsic apoptotic signaling pathway by p53 class mediator	1/17	31/17913	0.029029	0.086222	0.050043	4193	1
GO:1904893	GO:1904893	negative regulation of STAT cascade	1/17	31/17913	0.029029	0.086222	0.050043	8835	1
GO:0051235	GO:0051235	maintenance of location	2/17	286/17913	0.029502	0.086752	0.050351	84649/581	2
GO:0090150	GO:0090150	establishment of protein localization to membrane	2/17	287/17913	0.029692	0.086752	0.050351	3320/581	2
GO:0003299	GO:0003299	muscle hypertrophy in response to stress	1/17	32/17913	0.029952	0.086752	0.050351	2308	1
GO:0014887	GO:0014887	cardiac muscle adaptation	1/17	32/17913	0.029952	0.086752	0.050351	2308	1
GO:0014898	GO:0014898	cardiac muscle hypertrophy in response to stress	1/17	32/17913	0.029952	0.086752	0.050351	2308	1
GO:0030851	GO:0030851	granulocyte differentiation	1/17	32/17913	0.029952	0.086752	0.050351	2194	1
GO:0032733	GO:0032733	positive regulation of interleukin-10 production	1/17	32/17913	0.029952	0.086752	0.050351	3329	1
GO:0038128	GO:0038128	ERBB2 signaling pathway	1/17	32/17913	0.029952	0.086752	0.050351	3320	1
GO:0046949	GO:0046949	fatty-acyl-CoA biosynthetic process	1/17	32/17913	0.029952	0.086752	0.050351	2194	1
GO:0071295	GO:0071295	cellular response to vitamin	1/17	32/17913	0.029952	0.086752	0.050351	4193	1
GO:0072001	GO:0072001	renal system development	2/17	291/17913	0.03046	0.086752	0.050351	4609/581	2
GO:0006084	GO:0006084	acetyl-CoA metabolic process	1/17	33/17913	0.030874	0.086752	0.050351	2194	1
GO:0006308	GO:0006308	DNA catabolic process	1/17	33/17913	0.030874	0.086752	0.050351	581	1
GO:0006921	GO:0006921	cellular component disassembly involved in execution phase of apoptosis	1/17	33/17913	0.030874	0.086752	0.050351	581	1
GO:0010039	GO:0010039	response to iron ion	1/17	33/17913	0.030874	0.086752	0.050351	4193	1
GO:0032094	GO:0032094	response to food	1/17	33/17913	0.030874	0.086752	0.050351	2538	1
GO:0043032	GO:0043032	positive regulation of macrophage activation	1/17	33/17913	0.030874	0.086752	0.050351	3329	1
GO:0043094	GO:0043094	cellular metabolic compound salvage	1/17	33/17913	0.030874	0.086752	0.050351	7083	1
GO:0043516	GO:0043516	regulation of DNA damage response, signal transduction by p53 class mediator	1/17	33/17913	0.030874	0.086752	0.050351	4193	1
GO:0045922	GO:0045922	negative regulation of fatty acid metabolic process	1/17	33/17913	0.030874	0.086752	0.050351	84649	1
GO:0071353	GO:0071353	cellular response to interleukin-4	1/17	33/17913	0.030874	0.086752	0.050351	2194	1
GO:1901797	GO:1901797	negative regulation of signal transduction by p53 class mediator	1/17	33/17913	0.030874	0.086752	0.050351	4193	1
GO:1902742	GO:1902742	apoptotic process involved in development	1/17	33/17913	0.030874	0.086752	0.050351	581	1
GO:0010464	GO:0010464	regulation of mesenchymal cell proliferation	1/17	34/17913	0.031796	0.087493	0.050781	4609	1

	ID	Description	Generation	Bg Ratio	*P* value	*P*. adjust	Q value	Gene ID	Count
GO:0014072	GO:0014072	response to isoquinoline alkaloid	1/17	34/17913	0.031796	0.087493	0.050781	4193	1
GO:0021955	GO:0021955	central nervous system neuron axonogenesis	1/17	34/17913	0.031796	0.087493	0.050781	3320	1
GO:0032735	GO:0032735	positive regulation of interleukin-12 production	1/17	34/17913	0.031796	0.087493	0.050781	3329	1
GO:0035088	GO:0035088	establishment or maintenance of apical/basal cell polarity	1/17	34/17913	0.031796	0.087493	0.050781	6624	1
GO:0043278	GO:0043278	response to morphine	1/17	34/17913	0.031796	0.087493	0.050781	4193	1
GO:0061245	GO:0061245	establishment or maintenance of bipolar cell polarity	1/17	34/17913	0.031796	0.087493	0.050781	6624	1
GO:0097009	GO:0097009	energy homeostasis	1/17	34/17913	0.031796	0.087493	0.050781	2308	1
GO:1901030	GO:1901030	positive regulation of mitochondrial outer membrane permeabilization involved in apoptotic signaling pathway	1/17	34/17913	0.031796	0.087493	0.050781	581	1
GO:0060416	GO:0060416	response to growth hormone	1/17	35/17913	0.032716	0.08941	0.051893	8835	1
GO:0061077	GO:0061077	chaperone-mediated protein folding	1/17	35/17913	0.032716	0.08941	0.051893	3315	1
GO:0071312	GO:0071312	cellular response to alkaloid	1/17	35/17913	0.032716	0.08941	0.051893	4193	1
GO:0002755	GO:0002755	MyD88-dependent toll-like receptor signaling pathway	1/17	36/17913	0.033636	0.090886	0.05275	3329	1
GO:0046825	GO:0046825	regulation of protein export from nucleus	1/17	36/17913	0.033636	0.090886	0.05275	4193	1
GO:0055090	GO:0055090	acylglycerol homeostasis	1/17	36/17913	0.033636	0.090886	0.05275	84649	1
GO:0070328	GO:0070328	triglyceride homeostasis	1/17	36/17913	0.033636	0.090886	0.05275	84649	1
GO:0070670	GO:0070670	response to interleukin-4	1/17	36/17913	0.033636	0.090886	0.05275	2194	1
GO:0010614	GO:0010614	negative regulation of cardiac muscle hypertrophy	1/17	37/17913	0.034555	0.092327	0.053586	2308	1
GO:0010955	GO:0010955	negative regulation of protein processing	1/17	37/17913	0.034555	0.092327	0.053586	4193	1
GO:0043029	GO:0043029	T cell homeostasis	1/17	37/17913	0.034555	0.092327	0.053586	581	1
GO:1903318	GO:1903318	negative regulation of protein maturation	1/17	37/17913	0.034555	0.092327	0.053586	4193	1
GO:1905898	GO:1905898	positive regulation of response to endoplasmic reticulum stress	1/17	37/17913	0.034555	0.092327	0.053586	581	1
GO:0010506	GO:0010506	regulation of autophagy	2/17	314/17913	0.035025	0.093374	0.054194	3315/2308	2
GO:0002711	GO:0002711	positive regulation of T cell mediated immunity	1/17	38/17913	0.035473	0.094149	0.054644	3329	1
GO:1902042	GO:1902042	negative regulation of extrinsic apoptotic signaling pathway *via* death domain receptors	1/17	38/17913	0.035473	0.094149	0.054644	355	1
GO:0014741	GO:0014741	negative regulation of muscle hypertrophy	1/17	39/17913	0.036391	0.095525	0.055443	2308	1

Supplementary Table S2 Continued

	ID	Description	Generation	Bg Ratio	*P* value	*P*. adjust	Q value	Gene ID	Count
GO:0019432	GO:0019432	triglyceride biosynthetic process	1/17	39/17913	0.036391	0.095525	0.055443	84649	1
GO:0044275	GO:0044275	cellular carbohydrate catabolic process	1/17	39/17913	0.036391	0.095525	0.055443	2538	1
GO:0071548	GO:0071548	response to dexamethasone	1/17	39/17913	0.036391	0.095525	0.055443	2308	1
GO:0090207	GO:0090207	regulation of triglyceride metabolic process	1/17	39/17913	0.036391	0.095525	0.055443	84649	1
GO:0009615	GO:0009615	response to virus	2/17	323/17913	0.036881	0.096601	0.056067	8519/3315	2
GO:0001655	GO:0001655	urogenital system development	2/17	324/17913	0.037089	0.096869	0.056222	4609/581	2
GO:0038084	GO:0038084	vascular endothelial growth factor signaling pathway	1/17	40/17913	0.037307	0.096869	0.056222	3315	1
GO:0051281	GO:0051281	positive regulation of release of sequestered calcium ion into cytosol	1/17	40/17913	0.037307	0.096869	0.056222	581	1
GO:0061028	GO:0061028	establishment of endothelial barrier	1/17	40/17913	0.037307	0.096869	0.056222	2194	1
GO:0008631	GO:0008631	intrinsic apoptotic signaling pathway in response to oxidative stress	1/17	41/17913	0.038223	0.097139	0.056379	3315	1
GO:0009163	GO:0009163	nucleoside biosynthetic process	1/17	41/17913	0.038223	0.097139	0.056379	7083	1
GO:0046460	GO:0046460	neutral lipid biosynthetic process	1/17	41/17913	0.038223	0.097139	0.056379	84649	1
GO:0046463	GO:0046463	acylglycerol biosynthetic process	1/17	41/17913	0.038223	0.097139	0.056379	84649	1
GO:0048066	GO:0048066	developmental pigmentation	1/17	41/17913	0.038223	0.097139	0.056379	581	1
GO:0060612	GO:0060612	adipose tissue development	1/17	41/17913	0.038223	0.097139	0.056379	84649	1
GO:0090184	GO:0090184	positive regulation of kidney development	1/17	41/17913	0.038223	0.097139	0.056379	4609	1
GO:0098760	GO:0098760	response to interleukin-7	1/17	41/17913	0.038223	0.097139	0.056379	8835	1
GO:0098761	GO:0098761	cellular response to interleukin-7	1/17	41/17913	0.038223	0.097139	0.056379	8835	1
GO:1903053	GO:1903053	regulation of extracellular matrix organization	1/17	41/17913	0.038223	0.097139	0.056379	6624	1
GO:0001101	GO:0001101	response to acid chemical	2/17	330/17913	0.038351	0.097258	0.056448	2308/84649	2
GO:0032731	GO:0032731	positive regulation of interleukin-1 beta production	1/17	42/17913	0.039138	0.098419	0.057122	3315	1
GO:0045429	GO:0045429	positive regulation of nitric oxide biosynthetic process	1/17	42/17913	0.039138	0.098419	0.057122	3320	1
GO:0051489	GO:0051489	regulation of filopodium assembly	1/17	42/17913	0.039138	0.098419	0.057122	6624	1
GO:1901659	GO:1901659	glycosyl compound biosynthetic process	1/17	42/17913	0.039138	0.098419	0.057122	7083	1
GO:0008089	GO:0008089	anterograde axonal transport	1/17	43/17913	0.040052	0.099053	0.05749	3315	1
GO:0031018	GO:0031018	endocrine pancreas development	1/17	43/17913	0.040052	0.099053	0.05749	2308	1
GO:0034105	GO:0034105	positive regulation of tissue remodeling	1/17	43/17913	0.040052	0.099053	0.05749	581	1

	ID	Description	Generation	Bg Ratio	*P* value	*P*. adjust	Q value	Gene ID	Count
GO:0034198	GO:0034198	cellular response to amino acid starvation	1/17	43/17913	0.040052	0.099053	0.05749	355	1
GO:0060443	GO:0060443	mammary gland morphogenesis	1/17	43/17913	0.040052	0.099053	0.05749	581	1
GO:1901028	GO:1901028	regulation of mitochondrial outer membrane permeabilization involved in apoptotic signaling pathway	1/17	43/17913	0.040052	0.099053	0.05749	581	1
GO:1902743	GO:1902743	regulation of lamellipodium organization	1/17	43/17913	0.040052	0.099053	0.05749	6624	1
GO:1904407	GO:1904407	positive regulation of nitric oxide metabolic process	1/17	43/17913	0.040052	0.099053	0.05749	3320	1
GO:0006066	GO:0006066	alcohol metabolic process	2/17	339/17913	0.040273	0.099192	0.057571	2194/84649	2
GO:0006631	GO:0006631	fatty acid metabolic process	2/17	339/17913	0.040273	0.099192	0.057571	2194/84649	2
GO:0032459	GO:0032459	regulation of protein oligomerization	1/17	44/17913	0.040965	0.100276	0.0582	581	1
GO:0034383	GO:0034383	low-density lipoprotein particle clearance	1/17	44/17913	0.040965	0.100276	0.0582	84649	1
GO:0051205	GO:0051205	protein insertion into membrane	1/17	44/17913	0.040965	0.100276	0.0582	581	1
GO:0051260	GO:0051260	protein homo-oligomerization	2/17	345/17913	0.041576	0.101472	0.058894	7083/581	2
GO:0006790	GO:0006790	sulfur compound metabolic process	2/17	346/17913	0.041794	0.101472	0.058894	2194/84649	2
GO:0002833	GO:0002833	positive regulation of response to biotic stimulus	1/17	45/17913	0.041877	0.101472	0.058894	3329	1
GO:0010463	GO:0010463	mesenchymal cell proliferation	1/17	45/17913	0.041877	0.101472	0.058894	4609	1
GO:0070266	GO:0070266	necroptotic process	1/17	45/17913	0.041877	0.101472	0.058894	355	1
GO:0031648	GO:0031648	protein destabilization	1/17	46/17913	0.042789	0.102028	0.059217	4193	1
GO:0048806	GO:0048806	genitalia development	1/17	46/17913	0.042789	0.102028	0.059217	581	1
GO:0070231	GO:0070231	T cell apoptotic process	1/17	46/17913	0.042789	0.102028	0.059217	581	1
GO:0090199	GO:0090199	regulation of release of cytochrome c from mitochondria	1/17	46/17913	0.042789	0.102028	0.059217	581	1
GO:1900087	GO:1900087	positive regulation of G1/S transition of mitotic cell cycle	1/17	46/17913	0.042789	0.102028	0.059217	4193	1
GO:1904738	GO:1904738	vascular associated smooth muscle cell migration	1/17	46/17913	0.042789	0.102028	0.059217	4193	1
GO:1904752	GO:1904752	regulation of vascular associated smooth muscle cell migration	1/17	46/17913	0.042789	0.102028	0.059217	4193	1
GO:1990928	GO:1990928	response to amino acid starvation	1/17	46/17913	0.042789	0.102028	0.059217	355	1
GO:0051604	GO:0051604	protein maturation	2/17	353/17913	0.043337	0.102969	0.059763	3329/4193	2
GO:0002204	GO:0002204	somatic recombination of immunoglobulin genes involved in immune response	1/17	47/17913	0.043699	0.102969	0.059763	3329	1

Supplementary Table S2 Continued

	ID	Description	Generation	Bg Ratio	*P* value	*P*. adjust	Q value	Gene ID	Count
GO:0002208	GO:0002208	somatic diversification of immunoglobulins involved in immune response	1/17	47/17913	0.043699	0.102969	0.059763	3329	1
GO:0014911	GO:0014911	positive regulation of smooth muscle cell migration	1/17	47/17913	0.043699	0.102969	0.059763	4193	1
GO:0045023	GO:0045023	G0 to G1 transition	1/17	47/17913	0.043699	0.102969	0.059763	4194	1
GO:0045190	GO:0045190	isotype switching	1/17	47/17913	0.043699	0.102969	0.059763	3329	1
GO:0002762	GO:0002762	negative regulation of myeloid leukocyte differentiation	1/17	48/17913	0.044609	0.104496	0.060649	4609	1
GO:0032653	GO:0032653	regulation of interleukin-10 production	1/17	48/17913	0.044609	0.104496	0.060649	3329	1
GO:0042220	GO:0042220	response to cocaine	1/17	48/17913	0.044609	0.104496	0.060649	4193	1
GO:0007595	GO:0007595	lactation	1/17	49/17913	0.045519	0.106003	0.061524	8835	1
GO:0016447	GO:0016447	somatic recombination of immunoglobulin gene segments	1/17	49/17913	0.045519	0.106003	0.061524	3329	1
GO:1904707	GO:1904707	positive regulation of vascular smooth muscle cell proliferation	1/17	49/17913	0.045519	0.106003	0.061524	4193	1
GO:1901293	GO:1901293	nucleoside phosphate biosynthetic process	2/17	365/17913	0.046032	0.106871	0.062028	7083/2194	2
GO:0006732	GO:0006732	coenzyme metabolic process	2/17	366/17913	0.046259	0.106871	0.062028	2194/84649	2
GO:0032732	GO:0032732	positive regulation of interleukin-1 production	1/17	50/17913	0.046427	0.106871	0.062028	3315	1
GO:0045540	GO:0045540	regulation of cholesterol biosynthetic process	1/17	50/17913	0.046427	0.106871	0.062028	2194	1
GO:0055081	GO:0055081	anion homeostasis	1/17	50/17913	0.046427	0.106871	0.062028	84649	1
GO:0106118	GO:0106118	regulation of sterol biosynthetic process	1/17	50/17913	0.046427	0.106871	0.062028	2194	1
GO:0050678	GO:0050678	regulation of epithelial cell proliferation	2/17	369/17913	0.046944	0.107106	0.062164	4609/581	2
GO:0006213	GO:0006213	pyrimidine nucleoside metabolic process	1/17	51/17913	0.047334	0.107106	0.062164	7083	1
GO:0032613	GO:0032613	interleukin-10 production	1/17	51/17913	0.047334	0.107106	0.062164	3329	1
GO:0045599	GO:0045599	negative regulation of fat cell differentiation	1/17	51/17913	0.047334	0.107106	0.062164	2308	1
GO:0048146	GO:0048146	positive regulation of fibroblast proliferation	1/17	51/17913	0.047334	0.107106	0.062164	4609	1
GO:0050999	GO:0050999	regulation of nitric-oxide synthase activity	1/17	51/17913	0.047334	0.107106	0.062164	3320	1
GO:0070228	GO:0070228	regulation of lymphocyte apoptotic process	1/17	51/17913	0.047334	0.107106	0.062164	581	1
GO:0097366	GO:0097366	response to bronchodilator	1/17	51/17913	0.047334	0.107106	0.062164	2308	1
GO:0098930	GO:0098930	axonal transport	1/17	51/17913	0.047334	0.107106	0.062164	3315	1
GO:0072528	GO:0072528	pyrimidine-containing compound biosynthetic process	1/17	52/17913	0.048241	0.108731	0.063107	7083	1

	ID	Description	Generation	Bg Ratio	*P* value	*P*. adjust	Q value	Gene ID	Count
GO:0097345	GO:0097345	mitochondrial outer membrane permeabilization	1/17	52/17913	0.048241	0.108731	0.063107	581	1
GO:0048545	GO:0048545	response to steroid hormone	2/17	375/17913	0.048325	0.108731	0.063107	2308/4193	2
GO:0032655	GO:0032655	regulation of interleukin-12 production	1/17	53/17913	0.049147	0.109551	0.063583	3329	1
GO:0035384	GO:0035384	thioester biosynthetic process	1/17	53/17913	0.049147	0.109551	0.063583	2194	1
GO:0043551	GO:0043551	regulation of phosphatidylinositol 3-kinase activity	1/17	53/17913	0.049147	0.109551	0.063583	8835	1
GO:0050853	GO:0050853	B cell receptor signaling pathway	1/17	53/17913	0.049147	0.109551	0.063583	581	1
GO:0071616	GO:0071616	acyl-CoA biosynthetic process	1/17	53/17913	0.049147	0.109551	0.063583	2194	1
GO:0001541	GO:0001541	ovarian follicle development	1/17	54/17913	0.050052	0.110949	0.064394	581	1
GO:0010524	GO:0010524	positive regulation of calcium ion transport into cytosol	1/17	54/17913	0.050052	0.110949	0.064394	581	1
GO:0071385	GO:0071385	cellular response to glucocorticoid stimulus	1/17	54/17913	0.050052	0.110949	0.064394	2308	1
GO:0046486	GO:0046486	glycerolipid metabolic process	2/17	385/17913	0.05066	0.111917	0.064956	2538/84649	2
GO:0001885	GO:0001885	endothelial cell development	1/17	55/17913	0.050956	0.111917	0.064956	2194	1
GO:0002381	GO:0002381	immunoglobulin production involved in immunoglobulin mediated immune response	1/17	55/17913	0.050956	0.111917	0.064956	3329	1
GO:0016445	GO:0016445	somatic diversification of immunoglobulins	1/17	55/17913	0.050956	0.111917	0.064956	3329	1
GO:0032615	GO:0032615	interleukin-12 production	1/17	55/17913	0.050956	0.111917	0.064956	3329	1
GO:0001503	GO:0001503	ossification	2/17	388/17913	0.051368	0.112615	0.065362	8519/2194	2
GO:0014888	GO:0014888	striated muscle adaptation	1/17	56/17913	0.05186	0.112866	0.065507	2308	1
GO:0071398	GO:0071398	cellular response to fatty acid	1/17	56/17913	0.05186	0.112866	0.065507	84649	1
GO:1902808	GO:1902808	positive regulation of cell cycle G1/S phase transition	1/17	56/17913	0.05186	0.112866	0.065507	4193	1
GO:1903202	GO:1903202	negative regulation of oxidative stress-induced cell death	1/17	56/17913	0.05186	0.112866	0.065507	3315	1
GO:0001836	GO:0001836	release of cytochrome c from mitochondria	1/17	57/17913	0.052762	0.113184	0.065692	581	1
GO:0006879	GO:0006879	cellular iron ion homeostasis	1/17	57/17913	0.052762	0.113184	0.065692	4609	1
GO:0035418	GO:0035418	protein localization to synapse	1/17	57/17913	0.052762	0.113184	0.065692	3315	1
GO:0043525	GO:0043525	positive regulation of neuron apoptotic process	1/17	57/17913	0.052762	0.113184	0.065692	581	1
GO:0090183	GO:0090183	regulation of kidney development	1/17	57/17913	0.052762	0.113184	0.065692	4609	1
GO:1900408	GO:1900408	negative regulation of cellular response to oxidative stress	1/17	57/17913	0.052762	0.113184	0.065692	3315	1

Supplementary Table S2 Continued

	ID	Description	Generation	Bg Ratio	*P* value	*P*. adjust	Q value	Gene ID	Count
GO:1902041	GO:1902041	regulation of extrinsic apoptotic signaling pathway *via* death domain receptors	1/17	57/17913	0.052762	0.113184	0.065692	355	1
GO:1903428	GO:1903428	positive regulation of reactive oxygen species biosynthetic process	1/17	57/17913	0.052762	0.113184	0.065692	3320	1
GO:0046847	GO:0046847	filopodium assembly	1/17	58/17913	0.053664	0.114299	0.066339	6624	1
GO:0070527	GO:0070527	platelet aggregation	1/17	58/17913	0.053664	0.114299	0.066339	3315	1
GO:0071384	GO:0071384	cellular response to corticosteroid stimulus	1/17	58/17913	0.053664	0.114299	0.066339	2308	1
GO:1902110	GO:1902110	positive regulation of mitochondrial membrane permeability involved in apoptotic process	1/17	58/17913	0.053664	0.114299	0.066339	581	1
GO:0002429	GO:0002429	immune response-activating cell surface receptor signaling pathway	2/17	399/17913	0.053997	0.114804	0.066632	3320/581	2
GO:0045071	GO:0045071	negative regulation of viral genome replication	1/17	59/17913	0.054565	0.115397	0.066976	8519	1
GO:0070059	GO:0070059	intrinsic apoptotic signaling pathway in response to endoplasmic reticulum stress	1/17	59/17913	0.054565	0.115397	0.066976	581	1
GO:1902883	GO:1902883	negative regulation of response to oxidative stress	1/17	59/17913	0.054565	0.115397	0.066976	3315	1
GO:0001658	GO:0001658	branching involved in ureteric bud morphogenesis	1/17	60/17913	0.055465	0.11607	0.067367	4609	1
GO:0002260	GO:0002260	lymphocyte homeostasis	1/17	60/17913	0.055465	0.11607	0.067367	581	1
GO:0002562	GO:0002562	somatic diversification of immune receptors *via* germline recombination within a single locus	1/17	60/17913	0.055465	0.11607	0.067367	3329	1
GO:0016444	GO:0016444	somatic cell DNA recombination	1/17	60/17913	0.055465	0.11607	0.067367	3329	1
GO:0046824	GO:0046824	positive regulation of nucleocytoplasmic transport	1/17	60/17913	0.055465	0.11607	0.067367	4193	1
GO:1902686	GO:1902686	mitochondrial outer membrane permeabilization involved in programmed cell death	1/17	60/17913	0.055465	0.11607	0.067367	581	1
GO:0002709	GO:0002709	regulation of T cell mediated immunity	1/17	61/17913	0.056365	0.117133	0.067984	3329	1
GO:0008088	GO:0008088	axo-dendritic transport	1/17	61/17913	0.056365	0.117133	0.067984	3315	1
GO:0030032	GO:0030032	lamellipodium assembly	1/17	61/17913	0.056365	0.117133	0.067984	6624	1
GO:0042733	GO:0042733	embryonic digit morphogenesis	1/17	61/17913	0.056365	0.117133	0.067984	581	1
GO:0030099	GO:0030099	myeloid cell differentiation	2/17	411/17913	0.056921	0.117976	0.068473	4609/2194	2

	ID	Description	Generation	Bg Ratio	*P* value	*P*. adjust	Q value	Gene ID	Count
GO:0032729	GO:0032729	positive regulation of interferon-gamma production	1/17	62/17913	0.057263	0.117976	0.068473	3329	1
GO:0035794	GO:0035794	positive regulation of mitochondrial membrane permeability	1/17	62/17913	0.057263	0.117976	0.068473	581	1
GO:0043550	GO:0043550	regulation of lipid kinase activity	1/17	62/17913	0.057263	0.117976	0.068473	8835	1
GO:2001244	GO:2001244	positive regulation of intrinsic apoptotic signaling pathway	1/17	62/17913	0.057263	0.117976	0.068473	581	1
GO:0036498	GO:0036498	IRE1-mediated unfolded protein response	1/17	63/17913	0.058161	0.119415	0.069308	581	1
GO:0042108	GO:0042108	positive regulation of cytokine biosynthetic process	1/17	63/17913	0.058161	0.119415	0.069308	3315	1
GO:0019915	GO:0019915	lipid storage	1/17	64/17913	0.059058	0.12043	0.069898	84649	1
GO:0032768	GO:0032768	regulation of monooxygenase activity	1/17	64/17913	0.059058	0.12043	0.069898	3320	1
GO:1902108	GO:1902108	regulation of mitochondrial membrane permeability involved in apoptotic process	1/17	64/17913	0.059058	0.12043	0.069898	581	1
GO:1905710	GO:1905710	positive regulation of membrane permeability	1/17	64/17913	0.059058	0.12043	0.069898	581	1
GO:0006687	GO:0006687	glycosphingolipid metabolic process	1/17	65/17913	0.059954	0.12143	0.070478	581	1
GO:0035924	GO:0035924	cellular response to vascular endothelial growth factor stimulus	1/17	65/17913	0.059954	0.12143	0.070478	3315	1
GO:0045428	GO:0045428	regulation of nitric oxide biosynthetic process	1/17	65/17913	0.059954	0.12143	0.070478	3320	1
GO:0097194	GO:0097194	execution phase of apoptosis	1/17	65/17913	0.059954	0.12143	0.070478	581	1
GO:0050673	GO:0050673	epithelial cell proliferation	2/17	425/17913	0.060401	0.122129	0.070884	4609/581	2
GO:0043030	GO:0043030	regulation of macrophage activation	1/17	66/17913	0.06085	0.122364	0.07102	3329	1
GO:0060675	GO:0060675	ureteric bud morphogenesis	1/17	66/17913	0.06085	0.122364	0.07102	4609	1
GO:0002200	GO:0002200	somatic diversification of immune receptors	1/17	67/17913	0.061744	0.122364	0.07102	3329	1
GO:0007004	GO:0007004	telomere maintenance *via* telomerase	1/17	67/17913	0.061744	0.122364	0.07102	3320	1
GO:0033866	GO:0033866	nucleoside bisphosphate biosynthetic process	1/17	67/17913	0.061744	0.122364	0.07102	2194	1
GO:0034030	GO:0034030	ribonucleoside bisphosphate biosynthetic process	1/17	67/17913	0.061744	0.122364	0.07102	2194	1
GO:0034033	GO:0034033	purine nucleoside bisphosphate biosynthetic process	1/17	67/17913	0.061744	0.122364	0.07102	2194	1
GO:0040014	GO:0040014	regulation of multicellular organism growth	1/17	67/17913	0.061744	0.122364	0.07102	8835	1
GO:0045669	GO:0045669	positive regulation of osteoblast differentiation	1/17	67/17913	0.061744	0.122364	0.07102	8519	1
GO:0061180	GO:0061180	mammary gland epithelium development	1/17	67/17913	0.061744	0.122364	0.07102	581	1

Supplementary Table S2 Continued

	ID	Description	Generation	Bg Ratio	*P* value	*P*. adjust	Q value	Gene ID	Count
GO:0070227	GO:0070227	lymphocyte apoptotic process	1/17	67/17913	0.061744	0.122364	0.07102	581	1
GO:0072171	GO:0072171	mesonephric tubule morphogenesis	1/17	67/17913	0.061744	0.122364	0.07102	4609	1
GO:0032436	GO:0032436	positive regulation of proteasomal ubiquitin-dependent protein catabolic process	1/17	68/17913	0.062638	0.123805	0.071856	4193	1
GO:0002768	GO:0002768	immune response-regulating cell surface receptor signaling pathway	2/17	434/17913	0.062678	0.123805	0.071856	3320/581	2
GO:0006446	GO:0006446	regulation of translational initiation	1/17	69/17913	0.063531	0.124667	0.072356	3315	1
GO:0051865	GO:0051865	protein auto-ubiquitination	1/17	69/17913	0.063531	0.124667	0.072356	4193	1
GO:0071479	GO:0071479	cellular response to ionizing radiation	1/17	69/17913	0.063531	0.124667	0.072356	4193	1
GO:1901264	GO:1901264	carbohydrate derivative transport	1/17	69/17913	0.063531	0.124667	0.072356	2538	1
GO:0034381	GO:0034381	plasma lipoprotein particle clearance	1/17	70/17913	0.064423	0.126004	0.073133	84649	1
GO:1904427	GO:1904427	positive regulation of calcium ion transmembrane transport	1/17	70/17913	0.064423	0.126004	0.073133	581	1
GO:0042110	GO:0042110	T cell activation	2/17	443/17913	0.064985	0.126508	0.073425	3329/581	2
GO:0002312	GO:0002312	B cell activation involved in immune response	1/17	71/17913	0.065315	0.126508	0.073425	3329	1
GO:0005977	GO:0005977	glycogen metabolic process	1/17	71/17913	0.065315	0.126508	0.073425	2538	1
GO:0006305	GO:0006305	DNA alkylation	1/17	71/17913	0.065315	0.126508	0.073425	4609	1
GO:0006306	GO:0006306	DNA methylation	1/17	71/17913	0.065315	0.126508	0.073425	4609	1
GO:0031670	GO:0031670	cellular response to nutrient	1/17	71/17913	0.065315	0.126508	0.073425	4193	1
GO:0001819	GO:0001819	positive regulation of cytokine production	2/17	446/17913	0.06576	0.126796	0.073592	3329/3315	2
GO:0006073	GO:0006073	cellular glucan metabolic process	1/17	72/17913	0.066205	0.126796	0.073592	2538	1
GO:0006695	GO:0006695	cholesterol biosynthetic process	1/17	72/17913	0.066205	0.126796	0.073592	2194	1
GO:0044042	GO:0044042	glucan metabolic process	1/17	72/17913	0.066205	0.126796	0.073592	2538	1
GO:0072347	GO:0072347	response to anesthetic	1/17	72/17913	0.066205	0.126796	0.073592	4193	1
GO:1902117	GO:1902117	positive regulation of organelle assembly	1/17	72/17913	0.066205	0.126796	0.073592	6624	1
GO:1902653	GO:1902653	secondary alcohol biosynthetic process	1/17	72/17913	0.066205	0.126796	0.073592	2194	1
GO:0001895	GO:0001895	retina homeostasis	1/17	73/17913	0.067095	0.127683	0.074107	3315	1
GO:0021536	GO:0021536	diencephalon development	1/17	73/17913	0.067095	0.127683	0.074107	581	1
GO:0043627	GO:0043627	response to estrogen	1/17	73/17913	0.067095	0.127683	0.074107	4193	1
GO:0051881	GO:0051881	regulation of mitochondrial membrane potential	1/17	73/17913	0.067095	0.127683	0.074107	581	1
GO:0072594	GO:0072594	establishment of protein localization to organelle	2/17	452/17913	0.067321	0.12791	0.074239	3320/581	2

	ID	Description	Generation	Bg Ratio	*P* value	*P*. adjust	Q value	Gene ID	Count
GO:0006278	GO:0006278	RNA-dependent DNA biosynthetic process	1/17	74/17913	0.067984	0.128355	0.074497	3320	1
GO:0032481	GO:0032481	positive regulation of type I interferon production	1/17	74/17913	0.067984	0.128355	0.074497	3329	1
GO:0046902	GO:0046902	regulation of mitochondrial membrane permeability	1/17	74/17913	0.067984	0.128355	0.074497	581	1
GO:0055072	GO:0055072	iron ion homeostasis	1/17	74/17913	0.067984	0.128355	0.074497	4609	1
GO:0016126	GO:0016126	sterol biosynthetic process	1/17	75/17913	0.068872	0.129014	0.07488	2194	1
GO:0031016	GO:0031016	pancreas development	1/17	75/17913	0.068872	0.129014	0.07488	2308	1
GO:0048678	GO:0048678	response to axon injury	1/17	75/17913	0.068872	0.129014	0.07488	581	1
GO:0072078	GO:0072078	nephron tubule morphogenesis	1/17	75/17913	0.068872	0.129014	0.07488	4609	1
GO:1903201	GO:1903201	regulation of oxidative stress-induced cell death	1/17	75/17913	0.068872	0.129014	0.07488	3315	1
GO:0006809	GO:0006809	nitric oxide biosynthetic process	1/17	76/17913	0.06976	0.130066	0.07549	3320	1
GO:0010611	GO:0010611	regulation of cardiac muscle hypertrophy	1/17	76/17913	0.06976	0.130066	0.07549	2308	1
GO:0021954	GO:0021954	central nervous system neuron development	1/17	76/17913	0.06976	0.130066	0.07549	3320	1
GO:0034109	GO:0034109	homotypic cell-cell adhesion	1/17	77/17913	0.070646	0.130744	0.075883	3315	1
GO:0060411	GO:0060411	cardiac septum morphogenesis	1/17	77/17913	0.070646	0.130744	0.075883	4193	1
GO:0071260	GO:0071260	cellular response to mechanical stimulus	1/17	77/17913	0.070646	0.130744	0.075883	355	1
GO:0072088	GO:0072088	nephron epithelium morphogenesis	1/17	77/17913	0.070646	0.130744	0.075883	4609	1
GO:0007005	GO:0007005	mitochondrion organization	2/17	466/17913	0.071012	0.130744	0.075883	3320/581	2
GO:0016049	GO:0016049	cell growth	2/17	466/17913	0.071012	0.130744	0.075883	8835/3320	2
GO:0006875	GO:0006875	cellular metal ion homeostasis	2/17	467/17913	0.071278	0.130744	0.075883	4609/581	2
GO:0016925	GO:0016925	protein sumoylation	1/17	78/17913	0.071532	0.130744	0.075883	4193	1
GO:0022617	GO:0022617	extracellular matrix disassembly	1/17	78/17913	0.071532	0.130744	0.075883	6624	1
GO:0032760	GO:0032760	positive regulation of tumor necrosis factor production	1/17	78/17913	0.071532	0.130744	0.075883	3315	1
GO:0034308	GO:0034308	primary alcohol metabolic process	1/17	78/17913	0.071532	0.130744	0.075883	84649	1
GO:0051279	GO:0051279	regulation of release of sequestered calcium ion into cytosol	1/17	78/17913	0.071532	0.130744	0.075883	581	1
GO:0010833	GO:0010833	telomere maintenance *via* telomere lengthening	1/17	79/17913	0.072417	0.130744	0.075883	3320	1
GO:0014743	GO:0014743	regulation of muscle hypertrophy	1/17	79/17913	0.072417	0.130744	0.075883	2308	1
GO:0032204	GO:0032204	regulation of telomere maintenance	1/17	79/17913	0.072417	0.130744	0.075883	4609	1

Supplementary Table S2 Continued

	ID	Description	Generation	Bg Ratio	*P* value	*P*. adjust	Q value	Gene ID	Count
GO:0043154	GO:0043154	negative regulation of cysteine-type endopeptidase activity involved in apoptotic process	1/17	79/17913	0.072417	0.130744	0.075883	4193	1
GO:0061333	GO:0061333	renal tubule morphogenesis	1/17	79/17913	0.072417	0.130744	0.075883	4609	1
GO:0072028	GO:0072028	nephron morphogenesis	1/17	79/17913	0.072417	0.130744	0.075883	4609	1
GO:0097581	GO:0097581	lamellipodium organization	1/17	79/17913	0.072417	0.130744	0.075883	6624	1
GO:1900034	GO:1900034	regulation of cellular response to heat	1/17	79/17913	0.072417	0.130744	0.075883	3320	1
GO:2001021	GO:2001021	negative regulation of response to DNA damage stimulus	1/17	79/17913	0.072417	0.130744	0.075883	4193	1
GO:0043536	GO:0043536	positive regulation of blood vessel endothelial cell migration	1/17	80/17913	0.073301	0.132141	0.076694	3315	1
GO:0032755	GO:0032755	positive regulation of interleukin-6 production	1/17	81/17913	0.074185	0.132536	0.076924	3329	1
GO:0046209	GO:0046209	nitric oxide metabolic process	1/17	81/17913	0.074185	0.132536	0.076924	3320	1
GO:0046889	GO:0046889	positive regulation of lipid biosynthetic process	1/17	81/17913	0.074185	0.132536	0.076924	84649	1
GO:0051702	GO:0051702	interaction with symbiont	1/17	81/17913	0.074185	0.132536	0.076924	3329	1
GO:1902930	GO:1902930	regulation of alcohol biosynthetic process	1/17	81/17913	0.074185	0.132536	0.076924	2194	1
GO:1903557	GO:1903557	positive regulation of tumor necrosis factor superfamily cytokine production	1/17	81/17913	0.074185	0.132536	0.076924	3315	1
GO:0006970	GO:0006970	response to osmotic stress	1/17	82/17913	0.075067	0.133119	0.077262	581	1
GO:0032651	GO:0032651	regulation of interleukin-1 beta production	1/17	82/17913	0.075067	0.133119	0.077262	3315	1
GO:1901992	GO:1901992	positive regulation of mitotic cell cycle phase transition	1/17	82/17913	0.075067	0.133119	0.077262	4193	1
GO:2000060	GO:2000060	positive regulation of ubiquitin-dependent protein catabolic process	1/17	82/17913	0.075067	0.133119	0.077262	4193	1
GO:2000106	GO:2000106	regulation of leukocyte apoptotic process	1/17	82/17913	0.075067	0.133119	0.077262	581	1
GO:0046916	GO:0046916	cellular transition metal ion homeostasis	1/17	83/17913	0.075949	0.134285	0.077939	4609	1
GO:1905897	GO:1905897	regulation of response to endoplasmic reticulum stress	1/17	83/17913	0.075949	0.134285	0.077939	581	1
GO:0001776	GO:0001776	leukocyte homeostasis	1/17	84/17913	0.07683	0.135045	0.07838	581	1
GO:0006626	GO:0006626	protein targeting to mitochondrion	1/17	84/17913	0.07683	0.135045	0.07838	3320	1
GO:0090559	GO:0090559	regulation of membrane permeability	1/17	84/17913	0.07683	0.135045	0.07838	581	1
GO:2001057	GO:2001057	reactive nitrogen species metabolic process	1/17	84/17913	0.07683	0.135045	0.07838	3320	1

	ID	Description	Generation	Bg Ratio	*P* value	*P*. adjust	Q value	Gene ID	Count
GO:0014910	GO:0014910	regulation of smooth muscle cell migration	1/17	85/17913	0.07771	0.135993	0.07893	4193	1
GO:0070542	GO:0070542	response to fatty acid	1/17	85/17913	0.07771	0.135993	0.07893	84649	1
GO:1903901	GO:1903901	negative regulation of viral life cycle	1/17	85/17913	0.07771	0.135993	0.07893	8519	1
GO:0033138	GO:0033138	positive regulation of peptidyl-serine phosphorylation	1/17	86/17913	0.07859	0.136732	0.079359	3320	1
GO:0034103	GO:0034103	regulation of tissue remodeling	1/17	86/17913	0.07859	0.136732	0.079359	581	1
GO:1903725	GO:1903725	regulation of phospholipid metabolic process	1/17	86/17913	0.07859	0.136732	0.079359	8835	1
GO:2000177	GO:2000177	regulation of neural precursor cell proliferation	1/17	86/17913	0.07859	0.136732	0.079359	2308	1
GO:2000117	GO:2000117	negative regulation of cysteine-type endopeptidase activity	1/17	87/17913	0.079468	0.137786	0.079971	4193	1
GO:0050878	GO:0050878	regulation of body fluid levels	2/17	499/17913	0.079972	0.137786	0.079971	8835/3315	2
GO:0009150	GO:0009150	purine ribonucleotide metabolic process	2/17	500/17913	0.08025	0.137786	0.079971	2194/84649	2
GO:1901615	GO:1901615	organic hydroxy compound metabolic process	2/17	500/17913	0.08025	0.137786	0.079971	2194/84649	2
GO:0002456	GO:0002456	T cell mediated immunity	1/17	88/17913	0.080346	0.137786	0.079971	3329	1
GO:0009791	GO:0009791	post-embryonic development	1/17	88/17913	0.080346	0.137786	0.079971	581	1
GO:0044264	GO:0044264	cellular polysaccharide metabolic process	1/17	88/17913	0.080346	0.137786	0.079971	2538	1
GO:1900407	GO:1900407	regulation of cellular response to oxidative stress	1/17	88/17913	0.080346	0.137786	0.079971	3315	1
GO:1904705	GO:1904705	regulation of vascular smooth muscle cell proliferation	1/17	88/17913	0.080346	0.137786	0.079971	4193	1
GO:1990874	GO:1990874	vascular smooth muscle cell proliferation	1/17	88/17913	0.080346	0.137786	0.079971	4193	1
GO:0002824	GO:0002824	positive regulation of adaptive immune response based on somatic recombination of immune receptors built from immunoglobulin superfamily domains	1/17	89/17913	0.081223	0.138694	0.080498	3329	1
GO:0044728	GO:0044728	DNA methylation or demethylation	1/17	89/17913	0.081223	0.138694	0.080498	4609	1
GO:0051289	GO:0051289	protein homotetramerization	1/17	89/17913	0.081223	0.138694	0.080498	7083	1
GO:0001523	GO:0001523	retinoid metabolic process	1/17	91/17913	0.082975	0.139889	0.081191	84649	1
GO:0001656	GO:0001656	metanephros development	1/17	91/17913	0.082975	0.139889	0.081191	4609	1
GO:0002708	GO:0002708	positive regulation of lymphocyte mediated immunity	1/17	91/17913	0.082975	0.139889	0.081191	3329	1
GO:0014909	GO:0014909	smooth muscle cell migration	1/17	91/17913	0.082975	0.139889	0.081191	4193	1
GO:0043473	GO:0043473	pigmentation	1/17	91/17913	0.082975	0.139889	0.081191	581	1

Supplementary Table S2 Continued

	ID	Description	Generation	Bg Ratio	*P* value	*P*. adjust	Q value	Gene ID	Count
GO:0045833	GO:0045833	negative regulation of lipid metabolic process	1/17	91/17913	0.082975	0.139889	0.081191	84649	1
GO:0060337	GO:0060337	type I interferon signaling pathway	1/17	91/17913	0.082975	0.139889	0.081191	8519	1
GO:0071357	GO:0071357	cellular response to type I interferon	1/17	91/17913	0.082975	0.139889	0.081191	8519	1
GO:1901655	GO:1901655	cellular response to ketone	1/17	91/17913	0.082975	0.139889	0.081191	2308	1
GO:0036473	GO:0036473	cell death in response to oxidative stress	1/17	92/17913	0.08385	0.140439	0.081511	3315	1
GO:0045638	GO:0045638	negative regulation of myeloid cell differentiation	1/17	92/17913	0.08385	0.140439	0.081511	4609	1
GO:2001022	GO:2001022	positive regulation of response to DNA damage stimulus	1/17	92/17913	0.08385	0.140439	0.081511	4609	1
GO:0019395	GO:0019395	fatty acid oxidation	1/17	93/17913	0.084724	0.140439	0.081511	84649	1
GO:0032611	GO:0032611	interleukin-1 beta production	1/17	93/17913	0.084724	0.140439	0.081511	3315	1
GO:0032649	GO:0032649	regulation of interferon-gamma production	1/17	93/17913	0.084724	0.140439	0.081511	3329	1
GO:0072080	GO:0072080	nephron tubule development	1/17	93/17913	0.084724	0.140439	0.081511	4609	1
GO:1901216	GO:1901216	positive regulation of neuron death	1/17	93/17913	0.084724	0.140439	0.081511	581	1
GO:1901800	GO:1901800	positive regulation of proteasomal protein catabolic process	1/17	93/17913	0.084724	0.140439	0.081511	4193	1
GO:0002821	GO:0002821	positive regulation of adaptive immune response	1/17	94/17913	0.085597	0.140439	0.081511	3329	1
GO:0015748	GO:0015748	organophosphate ester transport	1/17	94/17913	0.085597	0.140439	0.081511	2538	1
GO:0045778	GO:0045778	positive regulation of ossification	1/17	94/17913	0.085597	0.140439	0.081511	8519	1
GO:0048284	GO:0048284	organelle fusion	1/17	94/17913	0.085597	0.140439	0.081511	581	1
GO:0048640	GO:0048640	negative regulation of developmental growth	1/17	94/17913	0.085597	0.140439	0.081511	8835	1
GO:0007589	GO:0007589	body fluid secretion	1/17	95/17913	0.086469	0.140439	0.081511	8835	1
GO:0008585	GO:0008585	female gonad development	1/17	95/17913	0.086469	0.140439	0.081511	581	1
GO:0019217	GO:0019217	regulation of fatty acid metabolic process	1/17	95/17913	0.086469	0.140439	0.081511	84649	1
GO:0033273	GO:0033273	response to vitamin	1/17	95/17913	0.086469	0.140439	0.081511	4193	1
GO:0034340	GO:0034340	response to type I interferon	1/17	95/17913	0.086469	0.140439	0.081511	8519	1
GO:0034440	GO:0034440	lipid oxidation	1/17	95/17913	0.086469	0.140439	0.081511	84649	1
GO:0045069	GO:0045069	regulation of viral genome replication	1/17	95/17913	0.086469	0.140439	0.081511	8519	1
GO:0050810	GO:0050810	regulation of steroid biosynthetic process	1/17	95/17913	0.086469	0.140439	0.081511	2194	1
GO:0060993	GO:0060993	kidney morphogenesis	1/17	95/17913	0.086469	0.140439	0.081511	4609	1

	ID	Description	Generation	Bg Ratio	*P* value	*P*. adjust	Q value	Gene ID	Count
GO:0061326	GO:0061326	renal tubule development	1/17	95/17913	0.086469	0.140439	0.081511	4609	1
GO:0072527	GO:0072527	pyrimidine-containing compound metabolic process	1/17	95/17913	0.086469	0.140439	0.081511	7083	1
GO:0097711	GO:0097711	ciliary basal body-plasma membrane docking	1/17	95/17913	0.086469	0.140439	0.081511	3320	1
GO:0120034	GO:0120034	positive regulation of plasma membrane bounded cell projection assembly	1/17	95/17913	0.086469	0.140439	0.081511	6624	1
GO:0032652	GO:0032652	regulation of interleukin-1 production	1/17	96/17913	0.087341	0.141089	0.081888	3315	1
GO:0042116	GO:0042116	macrophage activation	1/17	96/17913	0.087341	0.141089	0.081888	3329	1
GO:0097327	GO:0097327	response to antineoplastic agent	1/17	96/17913	0.087341	0.141089	0.081888	2308	1
GO:1901570	GO:1901570	fatty acid derivative biosynthetic process	1/17	96/17913	0.087341	0.141089	0.081888	2194	1
GO:0016101	GO:0016101	diterpenoid metabolic process	1/17	97/17913	0.088212	0.141921	0.082371	84649	1
GO:1901989	GO:1901989	positive regulation of cell cycle phase transition	1/17	97/17913	0.088212	0.141921	0.082371	4193	1
GO:1902882	GO:1902882	regulation of response to oxidative stress	1/17	97/17913	0.088212	0.141921	0.082371	3315	1
GO:0001657	GO:0001657	ureteric bud development	1/17	98/17913	0.089082	0.142175	0.082518	4609	1
GO:0002027	GO:0002027	regulation of heart rate	1/17	98/17913	0.089082	0.142175	0.082518	4193	1
GO:0002377	GO:0002377	immunoglobulin production	1/17	98/17913	0.089082	0.142175	0.082518	3329	1
GO:0007043	GO:0007043	cell-cell junction assembly	1/17	98/17913	0.089082	0.142175	0.082518	6624	1
GO:0010522	GO:0010522	regulation of calcium ion transport into cytosol	1/17	98/17913	0.089082	0.142175	0.082518	581	1
GO:0042136	GO:0042136	neurotransmitter biosynthetic process	1/17	98/17913	0.089082	0.142175	0.082518	3320	1
GO:0005976	GO:0005976	polysaccharide metabolic process	1/17	99/17913	0.089951	0.142611	0.082771	2538	1
GO:0048525	GO:0048525	negative regulation of viral process	1/17	99/17913	0.089951	0.142611	0.082771	8519	1
GO:0072163	GO:0072163	mesonephric epithelium development	1/17	99/17913	0.089951	0.142611	0.082771	4609	1
GO:0072164	GO:0072164	mesonephric tubule development	1/17	99/17913	0.089951	0.142611	0.082771	4609	1
GO:1902106	GO:1902106	negative regulation of leukocyte differentiation	1/17	99/17913	0.089951	0.142611	0.082771	4609	1
GO:0046545	GO:0046545	development of primary female sexual characteristics	1/17	100/17913	0.09082	0.143418	0.08324	581	1
GO:0046822	GO:0046822	regulation of nucleocytoplasmic transport	1/17	100/17913	0.09082	0.143418	0.08324	4193	1
GO:2001237	GO:2001237	negative regulation of extrinsic apoptotic signaling pathway	1/17	100/17913	0.09082	0.143418	0.08324	355	1
GO:0002367	GO:0002367	cytokine production involved in immune response	1/17	101/17913	0.091687	0.14384	0.083484	3329	1

Supplementary Table S2 Continued

	ID	Description	Generation	Bg Ratio	*P* value	*P*. adjust	Q value	Gene ID	Count
GO:0048661	GO:0048661	positive regulation of smooth muscle cell proliferation	1/17	101/17913	0.091687	0.14384	0.083484	4193	1
GO:0048675	GO:0048675	axon extension	1/17	101/17913	0.091687	0.14384	0.083484	3320	1
GO:1903426	GO:1903426	regulation of reactive oxygen species biosynthetic process	1/17	101/17913	0.091687	0.14384	0.083484	3320	1
GO:2000379	GO:2000379	positive regulation of reactive oxygen species metabolic process	1/17	101/17913	0.091687	0.14384	0.083484	3320	1
GO:0097006	GO:0097006	regulation of plasma lipoprotein particle levels	1/17	102/17913	0.092554	0.14501	0.084163	84649	1
GO:0001823	GO:0001823	mesonephros development	1/17	103/17913	0.09342	0.145604	0.084508	4609	1
GO:0014812	GO:0014812	muscle cell migration	1/17	103/17913	0.09342	0.145604	0.084508	4193	1
GO:0032091	GO:0032091	negative regulation of protein binding	1/17	103/17913	0.09342	0.145604	0.084508	581	1
GO:0071887	GO:0071887	leukocyte apoptotic process	1/17	103/17913	0.09342	0.145604	0.084508	581	1
GO:0032609	GO:0032609	interferon-gamma production	1/17	105/17913	0.09515	0.147532	0.085627	3329	1
GO:0045446	GO:0045446	endothelial cell differentiation	1/17	105/17913	0.09515	0.147532	0.085627	2194	1
GO:0051341	GO:0051341	regulation of oxidoreductase activity	1/17	105/17913	0.09515	0.147532	0.085627	3320	1
GO:0055076	GO:0055076	transition metal ion homeostasis	1/17	105/17913	0.09515	0.147532	0.085627	4609	1
GO:0006721	GO:0006721	terpenoid metabolic process	1/17	106/17913	0.096014	0.148104	0.085959	84649	1
GO:0021761	GO:0021761	limbic system development	1/17	106/17913	0.096014	0.148104	0.085959	581	1
GO:0043279	GO:0043279	response to alkaloid	1/17	106/17913	0.096014	0.148104	0.085959	4193	1
GO:0043502	GO:0043502	regulation of muscle adaptation	1/17	106/17913	0.096014	0.148104	0.085959	2308	1
GO:0062014	GO:0062014	negative regulation of small molecule metabolic process	1/17	107/17913	0.096877	0.149243	0.08662	84649	1
GO:0003300	GO:0003300	cardiac muscle hypertrophy	1/17	108/17913	0.097739	0.149992	0.087055	2308	1
GO:0006664	GO:0006664	glycolipid metabolic process	1/17	108/17913	0.097739	0.149992	0.087055	581	1
GO:0032612	GO:0032612	interleukin-1 production	1/17	108/17913	0.097739	0.149992	0.087055	3315	1
GO:0048593	GO:0048593	camera-type eye morphogenesis	1/17	109/17913	0.098601	0.150542	0.087374	581	1
GO:0072009	GO:0072009	nephron epithelium development	1/17	109/17913	0.098601	0.150542	0.087374	4609	1
GO:1903052	GO:1903052	positive regulation of proteolysis involved in cellular protein catabolic process	1/17	109/17913	0.098601	0.150542	0.087374	4193	1
GO:1903509	GO:1903509	liposaccharide metabolic process	1/17	109/17913	0.098601	0.150542	0.087374	581	1
GO:0032434	GO:0032434	regulation of proteasomal ubiquitin-dependent protein catabolic process	1/17	110/17913	0.099461	0.15147	0.087913	4193	1
GO:0042035	GO:0042035	regulation of cytokine biosynthetic process	1/17	110/17913	0.099461	0.15147	0.087913	3315	1

	ID	Description	Generation	Bg Ratio	*P* value	*P*. adjust	Q value	Gene ID	Count
GO:0014897	GO:0014897	striated muscle hypertrophy	1/17	111/17913	0.100321	0.152585	0.08856	2308	1
GO:0021987	GO:0021987	cerebral cortex development	1/17	112/17913	0.10118	0.15292	0.088755	581	1
GO:0030968	GO:0030968	endoplasmic reticulum unfolded protein response	1/17	112/17913	0.10118	0.15292	0.088755	581	1
GO:0042157	GO:0042157	lipoprotein metabolic process	1/17	112/17913	0.10118	0.15292	0.088755	84649	1
GO:0051209	GO:0051209	release of sequestered calcium ion into cytosol	1/17	112/17913	0.10118	0.15292	0.088755	581	1
GO:0051283	GO:0051283	negative regulation of sequestering of calcium ion	1/17	112/17913	0.10118	0.15292	0.088755	581	1
GO:0010508	GO:0010508	positive regulation of autophagy	1/17	113/17913	0.102039	0.153636	0.08917	2308	1
GO:0014896	GO:0014896	muscle hypertrophy	1/17	113/17913	0.102039	0.153636	0.08917	2308	1
GO:0030183	GO:0030183	B cell differentiation	1/17	113/17913	0.102039	0.153636	0.08917	581	1
GO:0006304	GO:0006304	DNA modification	1/17	114/17913	0.102896	0.154151	0.089469	4609	1
GO:0009116	GO:0009116	nucleoside metabolic process	1/17	114/17913	0.102896	0.154151	0.089469	7083	1
GO:0046660	GO:0046660	female sex differentiation	1/17	114/17913	0.102896	0.154151	0.089469	581	1
GO:0051282	GO:0051282	regulation of sequestering of calcium ion	1/17	114/17913	0.102896	0.154151	0.089469	581	1
GO:0010822	GO:0010822	positive regulation of mitochondrion organization	1/17	115/17913	0.103753	0.155047	0.089989	581	1
GO:0019751	GO:0019751	polyol metabolic process	1/17	115/17913	0.103753	0.155047	0.089989	84649	1
GO:0022612	GO:0022612	gland morphogenesis	1/17	116/17913	0.104609	0.155937	0.090506	581	1
GO:0032273	GO:0032273	positive regulation of protein polymerization	1/17	116/17913	0.104609	0.155937	0.090506	3320	1
GO:0002705	GO:0002705	positive regulation of leukocyte mediated immunity	1/17	117/17913	0.105465	0.156239	0.090681	3329	1
GO:0002761	GO:0002761	regulation of myeloid leukocyte differentiation	1/17	117/17913	0.105465	0.156239	0.090681	4609	1
GO:0034605	GO:0034605	cellular response to heat	1/17	117/17913	0.105465	0.156239	0.090681	3320	1
GO:0034754	GO:0034754	cellular hormone metabolic process	1/17	117/17913	0.105465	0.156239	0.090681	84649	1
GO:0051208	GO:0051208	sequestering of calcium ion	1/17	117/17913	0.105465	0.156239	0.090681	581	1
GO:0008637	GO:0008637	apoptotic mitochondrial changes	1/17	119/17913	0.107173	0.158378	0.091922	581	1
GO:0051928	GO:0051928	positive regulation of calcium ion transport	1/17	119/17913	0.107173	0.158378	0.091922	581	1
GO:0019079	GO:0019079	viral genome replication	1/17	120/17913	0.108026	0.159049	0.092312	8519	1
GO:0042089	GO:0042089	cytokine biosynthetic process	1/17	120/17913	0.108026	0.159049	0.092312	3315	1
GO:0046425	GO:0046425	regulation of JAK-STAT cascade	1/17	120/17913	0.108026	0.159049	0.092312	8835	1
GO:0006720	GO:0006720	isoprenoid metabolic process	1/17	121/17913	0.108878	0.159519	0.092585	84649	1

Supplementary Table S2 Continued

	ID	Description	Generation	Bg Ratio	*P* value	*P*. adjust	Q value	Gene ID	Count
GO:0042107	GO:0042107	cytokine metabolic process	1/17	121/17913	0.108878	0.159519	0.092585	3315	1
GO:0043500	GO:0043500	muscle adaptation	1/17	121/17913	0.108878	0.159519	0.092585	2308	1
GO:0046718	GO:0046718	viral entry into host cell	1/17	121/17913	0.108878	0.159519	0.092585	8519	1
GO:0035270	GO:0035270	endocrine system development	1/17	122/17913	0.10973	0.160374	0.093081	2308	1
GO:1903409	GO:1903409	reactive oxygen species biosynthetic process	1/17	122/17913	0.10973	0.160374	0.093081	3320	1
GO:0030326	GO:0030326	embryonic limb morphogenesis	1/17	123/17913	0.11058	0.160832	0.093347	581	1
GO:0032479	GO:0032479	regulation of type I interferon production	1/17	123/17913	0.11058	0.160832	0.093347	3329	1
GO:0034620	GO:0034620	cellular response to unfolded protein	1/17	123/17913	0.11058	0.160832	0.093347	581	1
GO:0035113	GO:0035113	embryonic appendage morphogenesis	1/17	123/17913	0.11058	0.160832	0.093347	581	1
GO:0003158	GO:0003158	endothelium development	1/17	124/17913	0.11143	0.161675	0.093836	2194	1
GO:0042177	GO:0042177	negative regulation of protein catabolic process	1/17	124/17913	0.11143	0.161675	0.093836	4194	1
GO:0032606	GO:0032606	type I interferon production	1/17	125/17913	0.112279	0.16271	0.094437	3329	1
GO:0060359	GO:0060359	response to ammonium ion	1/17	126/17913	0.113128	0.163543	0.09492	4193	1
GO:0097553	GO:0097553	calcium ion transmembrane import into cytosol	1/17	126/17913	0.113128	0.163543	0.09492	581	1
GO:0008286	GO:0008286	insulin receptor signaling pathway	1/17	128/17913	0.114822	0.165195	0.095879	2308	1
GO:0032355	GO:0032355	response to estradiol	1/17	128/17913	0.114822	0.165195	0.095879	8835	1
GO:0045216	GO:0045216	cell-cell junction organization	1/17	128/17913	0.114822	0.165195	0.095879	6624	1
GO:0045667	GO:0045667	regulation of osteoblast differentiation	1/17	128/17913	0.114822	0.165195	0.095879	8519	1
GO:0003206	GO:0003206	cardiac chamber morphogenesis	1/17	129/17913	0.115668	0.165814	0.096238	4193	1
GO:0010595	GO:0010595	positive regulation of endothelial cell migration	1/17	129/17913	0.115668	0.165814	0.096238	3315	1
GO:1904892	GO:1904892	regulation of STAT cascade	1/17	129/17913	0.115668	0.165814	0.096238	8835	1
GO:0050921	GO:0050921	positive regulation of chemotaxis	1/17	130/17913	0.116514	0.166826	0.096826	3315	1
GO:0045598	GO:0045598	regulation of fat cell differentiation	1/17	131/17913	0.117358	0.167835	0.097411	2308	1
GO:1901657	GO:1901657	glycosyl compound metabolic process	1/17	132/17913	0.118202	0.16884	0.097994	7083	1
GO:0030260	GO:0030260	entry into host cell	1/17	134/17913	0.119887	0.170434	0.09892	8519	1
GO:0044409	GO:0044409	entry into host	1/17	134/17913	0.119887	0.170434	0.09892	8519	1
GO:0051806	GO:0051806	entry into cell of other organism involved in symbiotic interaction	1/17	134/17913	0.119887	0.170434	0.09892	8519	1
GO:0051828	GO:0051828	entry into other organism involved in symbiotic interaction	1/17	134/17913	0.119887	0.170434	0.09892	8519	1

	ID	Description	Generation	Bg Ratio	*P* value	*P*. adjust	Q value	Gene ID	Count
GO:0002822	GO:0002822	regulation of adaptive immune response based on somatic recombination of immune receptors built from immunoglobulin superfamily domains	1/17	135/17913	0.120729	0.171021	0.09926	3329	1
GO:0010977	GO:0010977	negative regulation of neuron projection development	1/17	135/17913	0.120729	0.171021	0.09926	4193	1
GO:0035967	GO:0035967	cellular response to topologically incorrect protein	1/17	135/17913	0.120729	0.171021	0.09926	581	1
GO:0002706	GO:0002706	regulation of lymphocyte mediated immunity	1/17	136/17913	0.12157	0.171401	0.099481	3329	1
GO:0051017	GO:0051017	actin filament bundle assembly	1/17	136/17913	0.12157	0.171401	0.099481	6624	1
GO:0060041	GO:0060041	retina development in camera-type eye	1/17	136/17913	0.12157	0.171401	0.099481	581	1
GO:0071901	GO:0071901	negative regulation of protein serine/threonine kinase activity	1/17	136/17913	0.12157	0.171401	0.099481	3315	1
GO:0008584	GO:0008584	male gonad development	1/17	137/17913	0.12241	0.171977	0.099815	581	1
GO:0061572	GO:0061572	actin filament bundle organization	1/17	137/17913	0.12241	0.171977	0.099815	6624	1
GO:2000058	GO:2000058	regulation of ubiquitin-dependent protein catabolic process	1/17	137/17913	0.12241	0.171977	0.099815	4193	1
GO:0038127	GO:0038127	ERBB signaling pathway	1/17	138/17913	0.123249	0.172549	0.100147	3320	1
GO:0046546	GO:0046546	development of primary male sexual characteristics	1/17	138/17913	0.123249	0.172549	0.100147	581	1
GO:0048515	GO:0048515	spermatid differentiation	1/17	138/17913	0.123249	0.172549	0.100147	581	1
GO:0002433	GO:0002433	immune response-regulating cell surface receptor signaling pathway involved in phagocytosis	1/17	139/17913	0.124088	0.172713	0.100242	3320	1
GO:0002831	GO:0002831	regulation of response to biotic stimulus	1/17	139/17913	0.124088	0.172713	0.100242	3329	1
GO:0032675	GO:0032675	regulation of interleukin-6 production	1/17	139/17913	0.124088	0.172713	0.100242	3329	1
GO:0038096	GO:0038096	Fc-gamma receptor signaling pathway involved in phagocytosis	1/17	139/17913	0.124088	0.172713	0.100242	3320	1
GO:0051384	GO:0051384	response to glucocorticoid	1/17	139/17913	0.124088	0.172713	0.100242	2308	1
GO:0007259	GO:0007259	JAK-STAT cascade	1/17	140/17913	0.124925	0.173074	0.100452	8835	1
GO:0017038	GO:0017038	protein import	1/17	140/17913	0.124925	0.173074	0.100452	3320	1
GO:0072073	GO:0072073	kidney epithelium development	1/17	140/17913	0.124925	0.173074	0.100452	4609	1
GO:1904064	GO:1904064	positive regulation of cation transmembrane transport	1/17	140/17913	0.124925	0.173074	0.100452	581	1
GO:0038094	GO:0038094	Fc-gamma receptor signaling pathway	1/17	141/17913	0.125762	0.173831	0.100891	3320	1
GO:0140056	GO:0140056	organelle localization by membrane tethering	1/17	141/17913	0.125762	0.173831	0.100891	3320	1

Supplementary Table S2 Continued

	ID	Description	Generation	Bg Ratio	*P* value	*P*. adjust	Q value	Gene ID	Count
GO:0001678	GO:0001678	cellular glucose homeostasis	1/17	142/17913	0.126599	0.174383	0.101211	2308	1
GO:0002431	GO:0002431	Fc receptor mediated stimulatory signaling pathway	1/17	142/17913	0.126599	0.174383	0.101211	3320	1
GO:0072006	GO:0072006	nephron development	1/17	142/17913	0.126599	0.174383	0.101211	4609	1
GO:0010970	GO:0010970	transport along microtubule	1/17	143/17913	0.127434	0.175131	0.101646	3315	1
GO:0099111	GO:0099111	microtubule-based transport	1/17	143/17913	0.127434	0.175131	0.101646	3315	1
GO:0006633	GO:0006633	fatty acid biosynthetic process	1/17	144/17913	0.128269	0.175272	0.101727	2194	1
GO:0006665	GO:0006665	sphingolipid metabolic process	1/17	144/17913	0.128269	0.175272	0.101727	581	1
GO:0045834	GO:0045834	positive regulation of lipid metabolic process	1/17	144/17913	0.128269	0.175272	0.101727	84649	1
GO:0061351	GO:0061351	neural precursor cell proliferation	1/17	144/17913	0.128269	0.175272	0.101727	2308	1
GO:1903169	GO:1903169	regulation of calcium ion transmembrane transport	1/17	144/17913	0.128269	0.175272	0.101727	581	1
GO:0002262	GO:0002262	myeloid cell homeostasis	1/17	145/17913	0.129103	0.17541	0.101808	581	1
GO:0042133	GO:0042133	neurotransmitter metabolic process	1/17	145/17913	0.129103	0.17541	0.101808	3320	1
GO:0046165	GO:0046165	alcohol biosynthetic process	1/17	145/17913	0.129103	0.17541	0.101808	2194	1
GO:0048592	GO:0048592	eye morphogenesis	1/17	145/17913	0.129103	0.17541	0.101808	581	1
GO:0060402	GO:0060402	calcium ion transport into cytosol	1/17	145/17913	0.129103	0.17541	0.101808	581	1
GO:0035107	GO:0035107	appendage morphogenesis	1/17	146/17913	0.129936	0.175744	0.102002	581	1
GO:0035108	GO:0035108	limb morphogenesis	1/17	146/17913	0.129936	0.175744	0.102002	581	1
GO:0043524	GO:0043524	negative regulation of neuron apoptotic process	1/17	146/17913	0.129936	0.175744	0.102002	581	1
GO:0048754	GO:0048754	branching morphogenesis of an epithelial tube	1/17	146/17913	0.129936	0.175744	0.102002	4609	1
GO:0002224	GO:0002224	toll-like receptor signaling pathway	1/17	147/17913	0.130769	0.176471	0.102424	3329	1
GO:1903900	GO:1903900	regulation of viral life cycle	1/17	147/17913	0.130769	0.176471	0.102424	8519	1
GO:1990138	GO:1990138	neuron projection extension	1/17	148/17913	0.1316	0.177394	0.102959	3320	1
GO:0032635	GO:0032635	interleukin-6 production	1/17	149/17913	0.132431	0.178313	0.103493	3329	1
GO:0002819	GO:0002819	regulation of adaptive immune response	1/17	150/17913	0.133262	0.178627	0.103675	3329	1
GO:0022406	GO:0022406	membrane docking	1/17	150/17913	0.133262	0.178627	0.103675	3320	1
GO:0090316	GO:0090316	positive regulation of intracellular protein transport	1/17	150/17913	0.133262	0.178627	0.103675	4193	1
GO:0097696	GO:0097696	STAT cascade	1/17	150/17913	0.133262	0.178627	0.103675	8835	1
GO:0032680	GO:0032680	regulation of tumor necrosis factor production	1/17	151/17913	0.134091	0.179338	0.104087	3315	1

	ID	Description	Generation	Bg Ratio	*P* value	*P*. adjust	Q value	Gene ID	Count
GO:2001236	GO:2001236	regulation of extrinsic apoptotic signaling pathway	1/17	151/17913	0.134091	0.179338	0.104087	355	1
GO:0001764	GO:0001764	neuron migration	1/17	152/17913	0.13492	0.179643	0.104265	581	1
GO:0030168	GO:0030168	platelet activation	1/17	152/17913	0.13492	0.179643	0.104265	3315	1
GO:0034767	GO:0034767	positive regulation of ion transmembrane transport	1/17	152/17913	0.13492	0.179643	0.104265	581	1
GO:1903707	GO:1903707	negative regulation of hemopoiesis	1/17	152/17913	0.13492	0.179643	0.104265	4609	1
GO:0030705	GO:0030705	cytoskeleton-dependent intracellular transport	1/17	154/17913	0.136575	0.181242	0.105193	3315	1
GO:0031214	GO:0031214	biomineral tissue development	1/17	154/17913	0.136575	0.181242	0.105193	2308	1
GO:0045931	GO:0045931	positive regulation of mitotic cell cycle	1/17	154/17913	0.136575	0.181242	0.105193	4193	1
GO:0031960	GO:0031960	response to corticosteroid	1/17	155/17913	0.137402	0.181735	0.105479	2308	1
GO:0032640	GO:0032640	tumor necrosis factor production	1/17	155/17913	0.137402	0.181735	0.105479	3315	1
GO:1903555	GO:1903555	regulation of tumor necrosis factor superfamily cytokine production	1/17	155/17913	0.137402	0.181735	0.105479	3315	1
GO:0006338	GO:0006338	chromatin remodeling	1/17	156/17913	0.138228	0.182625	0.105995	4609	1
GO:0060401	GO:0060401	cytosolic calcium ion transport	1/17	157/17913	0.139053	0.183513	0.10651	581	1
GO:0002285	GO:0002285	lymphocyte activation involved in immune response	1/17	158/17913	0.139877	0.183588	0.106554	3329	1
GO:0009408	GO:0009408	response to heat	1/17	158/17913	0.139877	0.183588	0.106554	3320	1
GO:0019827	GO:0019827	stem cell population maintenance	1/17	158/17913	0.139877	0.183588	0.106554	2308	1
GO:0043488	GO:0043488	regulation of mRNA stability	1/17	158/17913	0.139877	0.183588	0.106554	3315	1
GO:0043535	GO:0043535	regulation of blood vessel endothelial cell migration	1/17	158/17913	0.139877	0.183588	0.106554	3315	1
GO:0046661	GO:0046661	male sex differentiation	1/17	160/17913	0.141523	0.185343	0.107573	581	1
GO:0098727	GO:0098727	maintenance of cell number	1/17	160/17913	0.141523	0.185343	0.107573	2308	1
GO:0071706	GO:0071706	tumor necrosis factor superfamily cytokine production	1/17	161/17913	0.142345	0.186216	0.108079	3315	1
GO:0051100	GO:0051100	negative regulation of binding	1/17	162/17913	0.143167	0.186882	0.108466	581	1
GO:0051302	GO:0051302	regulation of cell division	1/17	162/17913	0.143167	0.186882	0.108466	4609	1
GO:0006611	GO:0006611	protein export from nucleus	1/17	163/17913	0.143987	0.187544	0.10885	4193	1
GO:0043487	GO:0043487	regulation of RNA stability	1/17	163/17913	0.143987	0.187544	0.10885	3315	1
GO:0071466	GO:0071466	cellular response to xenobiotic stimulus	1/17	164/17913	0.144807	0.188407	0.109351	2308	1
GO:0021543	GO:0021543	pallium development	1/17	166/17913	0.146445	0.189712	0.110109	581	1
GO:0031345	GO:0031345	negative regulation of cell projection organization	1/17	166/17913	0.146445	0.189712	0.110109	4193	1

Supplementary Table S2 Continued

	ID	Description	Generation	Bg Ratio	*P* value	*P*. adjust	Q value	Gene ID	Count
GO:0051262	GO:0051262	protein tetramerization	1/17	166/17913	0.146445	0.189712	0.110109	7083	1
GO:0120032	GO:0120032	regulation of plasma membrane bounded cell projection assembly	1/17	166/17913	0.146445	0.189712	0.110109	6624	1
GO:0061136	GO:0061136	regulation of proteasomal protein catabolic process	1/17	167/17913	0.147262	0.190565	0.110604	4193	1
GO:0060491	GO:0060491	regulation of cell projection assembly	1/17	168/17913	0.148079	0.191415	0.111097	6624	1
GO:0090090	GO:0090090	negative regulation of canonical Wnt signaling pathway	1/17	169/17913	0.148895	0.192263	0.111589	2308	1
GO:0009566	GO:0009566	fertilization	1/17	170/17913	0.149711	0.193107	0.112079	581	1
GO:0016064	GO:0016064	immunoglobulin mediated immune response	1/17	171/17913	0.150525	0.193949	0.112568	3329	1
GO:0001659	GO:0001659	temperature homeostasis	1/17	172/17913	0.151339	0.194579	0.112933	2308	1
GO:0048660	GO:0048660	regulation of smooth muscle cell proliferation	1/17	172/17913	0.151339	0.194579	0.112933	4193	1
GO:0010634	GO:0010634	positive regulation of epithelial cell migration	1/17	173/17913	0.152152	0.19458	0.112934	3315	1
GO:0019724	GO:0019724	B cell mediated immunity	1/17	173/17913	0.152152	0.19458	0.112934	3329	1
GO:0034341	GO:0034341	response to interferon-gamma	1/17	173/17913	0.152152	0.19458	0.112934	8519	1
GO:0051897	GO:0051897	positive regulation of protein kinase B signaling	1/17	173/17913	0.152152	0.19458	0.112934	3320	1
GO:0070613	GO:0070613	regulation of protein processing	1/17	173/17913	0.152152	0.19458	0.112934	4193	1
GO:0048659	GO:0048659	smooth muscle cell proliferation	1/17	174/17913	0.152965	0.19541	0.113416	4193	1
GO:0010821	GO:0010821	regulation of mitochondrion organization	1/17	175/17913	0.153777	0.19582	0.113654	581	1
GO:0015893	GO:0015893	drug transport	1/17	175/17913	0.153777	0.19582	0.113654	4609	1
GO:1903317	GO:1903317	regulation of protein maturation	1/17	175/17913	0.153777	0.19582	0.113654	4193	1
GO:0048736	GO:0048736	appendage development	1/17	176/17913	0.154588	0.196226	0.113889	581	1
GO:0051168	GO:0051168	nuclear export	1/17	176/17913	0.154588	0.196226	0.113889	4193	1
GO:0060173	GO:0060173	limb development	1/17	176/17913	0.154588	0.196226	0.113889	581	1
GO:0021953	GO:0021953	central nervous system neuron differentiation	1/17	177/17913	0.155398	0.196837	0.114244	3320	1
GO:0042098	GO:0042098	T cell proliferation	1/17	177/17913	0.155398	0.196837	0.114244	581	1
GO:0006367	GO:0006367	transcription initiation from RNA polymerase II promoter	1/17	178/17913	0.156208	0.197029	0.114355	581	1
GO:0010565	GO:0010565	regulation of cellular ketone metabolic process	1/17	178/17913	0.156208	0.197029	0.114355	84649	1
GO:0061013	GO:0061013	regulation of mRNA catabolic process	1/17	178/17913	0.156208	0.197029	0.114355	3315	1

	ID	Description	Generation	Bg Ratio	*P* value	*P*. adjust	Q value	Gene ID	Count
GO:0061138	GO:0061138	morphogenesis of a branching epithelium	1/17	178/17913	0.156208	0.197029	0.114355	4609	1
GO:0007219	GO:0007219	Notch signaling pathway	1/17	179/17913	0.157016	0.197841	0.114826	4609	1
GO:0032271	GO:0032271	regulation of protein polymerization	1/17	180/17913	0.157824	0.19865	0.115296	3320	1
GO:0043534	GO:0043534	blood vessel endothelial cell migration	1/17	182/17913	0.159438	0.20026	0.116231	3315	1
GO:0043901	GO:0043901	negative regulation of multi-organism process	1/17	182/17913	0.159438	0.20026	0.116231	8519	1
GO:0006413	GO:0006413	translational initiation	1/17	183/17913	0.160244	0.201061	0.116696	3315	1
GO:0002703	GO:0002703	regulation of leukocyte mediated immunity	1/17	185/17913	0.161854	0.202233	0.117376	3329	1
GO:0010389	GO:0010389	regulation of G2/M transition of mitotic cell cycle	1/17	185/17913	0.161854	0.202233	0.117376	3320	1
GO:0016052	GO:0016052	carbohydrate catabolic process	1/17	185/17913	0.161854	0.202233	0.117376	2538	1
GO:1902115	GO:1902115	regulation of organelle assembly	1/17	185/17913	0.161854	0.202233	0.117376	6624	1
GO:0050920	GO:0050920	regulation of chemotaxis	1/17	186/17913	0.162658	0.203025	0.117836	3315	1
GO:0006694	GO:0006694	steroid biosynthetic process	1/17	190/17913	0.165865	0.206598	0.119909	2194	1
GO:0044272	GO:0044272	sulfur compound biosynthetic process	1/17	190/17913	0.165865	0.206598	0.119909	2194	1
GO:0002440	GO:0002440	production of molecular mediator of immune response	1/17	191/17913	0.166666	0.206949	0.120113	3329	1
GO:0006643	GO:0006643	membrane lipid metabolic process	1/17	191/17913	0.166666	0.206949	0.120113	581	1
GO:1901654	GO:1901654	response to ketone	1/17	191/17913	0.166666	0.206949	0.120113	2308	1
GO:0001763	GO:0001763	morphogenesis of a branching structure	1/17	192/17913	0.167465	0.207511	0.120439	4609	1
GO:0030258	GO:0030258	lipid modification	1/17	192/17913	0.167465	0.207511	0.120439	84649	1
GO:0071241	GO:0071241	cellular response to inorganic substance	1/17	194/17913	0.169062	0.209273	0.121461	2308	1
GO:0006473	GO:0006473	protein acetylation	1/17	195/17913	0.169859	0.210042	0.121908	2308	1
GO:0009124	GO:0009124	nucleoside monophosphate biosynthetic process	1/17	196/17913	0.170655	0.210592	0.122227	7083	1
GO:0031396	GO:0031396	regulation of protein ubiquitination	1/17	196/17913	0.170655	0.210592	0.122227	3320	1
GO:0002221	GO:0002221	pattern recognition receptor signaling pathway	1/17	197/17913	0.171451	0.211139	0.122545	3329	1
GO:0032869	GO:0032869	cellular response to insulin stimulus	1/17	197/17913	0.171451	0.211139	0.122545	2308	1
GO:0002699	GO:0002699	positive regulation of immune effector process	1/17	198/17913	0.172246	0.211141	0.122546	3329	1
GO:0030178	GO:0030178	negative regulation of Wnt signaling pathway	1/17	198/17913	0.172246	0.211141	0.122546	2308	1
GO:0050870	GO:0050870	positive regulation of T cell activation	1/17	198/17913	0.172246	0.211141	0.122546	3329	1

Supplementary Table S2 Continued

	ID	Description	Generation	Bg Ratio	*P* value	*P*. adjust	Q value	Gene ID	Count
GO:1903050	GO:1903050	regulation of proteolysis involved in cellular protein catabolic process	1/17	198/17913	0.172246	0.211141	0.122546	4193	1
GO:0002064	GO:0002064	epithelial cell development	1/17	199/17913	0.17304	0.211141	0.122546	2194	1
GO:0030278	GO:0030278	regulation of ossification	1/17	199/17913	0.17304	0.211141	0.122546	8519	1
GO:0034764	GO:0034764	positive regulation of transmembrane transport	1/17	199/17913	0.17304	0.211141	0.122546	581	1
GO:0050679	GO:0050679	positive regulation of epithelial cell proliferation	1/17	199/17913	0.17304	0.211141	0.122546	4609	1
GO:0071229	GO:0071229	cellular response to acid chemical	1/17	199/17913	0.17304	0.211141	0.122546	84649	1
GO:0009612	GO:0009612	response to mechanical stimulus	1/17	200/17913	0.173834	0.211893	0.122983	355	1
GO:0045665	GO:0045665	negative regulation of neuron differentiation	1/17	201/17913	0.174627	0.212643	0.123418	4193	1
GO:1902749	GO:1902749	regulation of cell cycle G2/M phase transition	1/17	202/17913	0.175419	0.213391	0.123852	3320	1
GO:0032388	GO:0032388	positive regulation of intracellular transport	1/17	203/17913	0.176211	0.213919	0.124158	4193	1
GO:0045766	GO:0045766	positive regulation of angiogenesis	1/17	203/17913	0.176211	0.213919	0.124158	3315	1
GO:0050792	GO:0050792	regulation of viral process	1/17	204/17913	0.177001	0.214661	0.124589	8519	1
GO:1901215	GO:1901215	negative regulation of neuron death	1/17	207/17913	0.179369	0.217313	0.126128	581	1
GO:0022618	GO:0022618	ribonucleoprotein complex assembly	1/17	209/17913	0.180945	0.219	0.127107	3320	1
GO:0010951	GO:0010951	negative regulation of endopeptidase activity	1/17	210/17913	0.181731	0.219508	0.127402	4193	1
GO:0043523	GO:0043523	regulation of neuron apoptotic process	1/17	210/17913	0.181731	0.219508	0.127402	581	1
GO:0034404	GO:0034404	nucleobase-containing small molecule biosynthetic process	1/17	211/17913	0.182517	0.220013	0.127695	7083	1
GO:0051701	GO:0051701	interaction with host	1/17	211/17913	0.182517	0.220013	0.127695	8519	1
GO:0034329	GO:0034329	cell junction assembly	1/17	214/17913	0.18487	0.222178	0.128952	6624	1
GO:0060560	GO:0060560	developmental growth involved in morphogenesis	1/17	214/17913	0.18487	0.222178	0.128952	3320	1
GO:1903039	GO:1903039	positive regulation of leukocyte cell-cell adhesion	1/17	214/17913	0.18487	0.222178	0.128952	3329	1
GO:0007584	GO:0007584	response to nutrient	1/17	215/17913	0.185653	0.222672	0.129238	4193	1
GO:0042445	GO:0042445	hormone metabolic process	1/17	215/17913	0.185653	0.222672	0.129238	84649	1
GO:0048588	GO:0048588	developmental cell growth	1/17	216/17913	0.186435	0.223386	0.129653	3320	1
GO:0008406	GO:0008406	gonad development	1/17	217/17913	0.187217	0.223874	0.129936	581	1
GO:0043393	GO:0043393	regulation of protein binding	1/17	217/17913	0.187217	0.223874	0.129936	581	1
GO:0033157	GO:0033157	regulation of intracellular protein transport	1/17	220/17913	0.189557	0.226447	0.131429	4193	1

	ID	Description	Generation	Bg Ratio	*P* value	*P*. adjust	Q value	Gene ID	Count
GO:0071826	GO:0071826	ribonucleoprotein complex subunit organization	1/17	221/17913	0.190336	0.22715	0.131838	3320	1
GO:0006469	GO:0006469	negative regulation of protein kinase activity	1/17	222/17913	0.191114	0.227625	0.132113	3315	1
GO:0045444	GO:0045444	fat cell differentiation	1/17	222/17913	0.191114	0.227625	0.132113	2308	1
GO:0010466	GO:0010466	negative regulation of peptidase activity	1/17	223/17913	0.191891	0.227645	0.132125	4193	1
GO:0034504	GO:0034504	protein localization to nucleus	1/17	223/17913	0.191891	0.227645	0.132125	4193	1
GO:0045137	GO:0045137	development of primary sexual characteristics	1/17	223/17913	0.191891	0.227645	0.132125	581	1
GO:1903320	GO:1903320	regulation of protein modification by small protein conjugation or removal	1/17	223/17913	0.191891	0.227645	0.132125	3320	1
GO:0007018	GO:0007018	microtubule-based movement	1/17	224/17913	0.192667	0.227888	0.132266	3315	1
GO:0043122	GO:0043122	regulation of I-kappaB kinase/NF-kappaB signaling	1/17	224/17913	0.192667	0.227888	0.132266	3315	1
GO:0050851	GO:0050851	antigen receptor-mediated signaling pathway	1/17	224/17913	0.192667	0.227888	0.132266	581	1
GO:0006352	GO:0006352	DNA-templated transcription, initiation	1/17	231/17913	0.198084	0.233833	0.135716	581	1
GO:0010594	GO:0010594	regulation of endothelial cell migration	1/17	231/17913	0.198084	0.233833	0.135716	3315	1
GO:0043903	GO:0043903	regulation of symbiosis, encompassing mutualism through parasitism	1/17	232/17913	0.198855	0.23405	0.135843	8519	1
GO:0051258	GO:0051258	protein polymerization	1/17	232/17913	0.198855	0.23405	0.135843	3320	1
GO:1904018	GO:1904018	positive regulation of vasculature development	1/17	232/17913	0.198855	0.23405	0.135843	3315	1
GO:0043543	GO:0043543	protein acylation	1/17	233/17913	0.199625	0.234726	0.136235	2308	1
GO:0000086	GO:0000086	G2/M transition of mitotic cell cycle	1/17	238/17913	0.203466	0.23854	0.138448	3320	1
GO:0007281	GO:0007281	germ cell development	1/17	238/17913	0.203466	0.23854	0.138448	581	1
GO:0051402	GO:0051402	neuron apoptotic process	1/17	238/17913	0.203466	0.23854	0.138448	581	1
GO:0071824	GO:0071824	protein-DNA complex subunit organization	1/17	240/17913	0.204998	0.239822	0.139193	4609	1
GO:0038093	GO:0038093	Fc receptor signaling pathway	1/17	241/17913	0.205762	0.239822	0.139193	3320	1
GO:0042180	GO:0042180	cellular ketone metabolic process	1/17	241/17913	0.205762	0.239822	0.139193	84649	1
GO:0048872	GO:0048872	homeostasis of number of cells	1/17	241/17913	0.205762	0.239822	0.139193	581	1
GO:0051896	GO:0051896	regulation of protein kinase B signaling	1/17	241/17913	0.205762	0.239822	0.139193	3320	1
GO:1901617	GO:1901617	organic hydroxy compound biosynthetic process	1/17	241/17913	0.205762	0.239822	0.139193	2194	1
GO:0033002	GO:0033002	muscle cell proliferation	1/17	243/17913	0.20729	0.24085	0.139789	4193	1
GO:0061448	GO:0061448	connective tissue development	1/17	243/17913	0.20729	0.24085	0.139789	84649	1

Supplementary Table S2 Continued

	ID	Description	Generation	Bg Ratio	*P* value	*P*. adjust	Q value	Gene ID	Count
GO:0071383	GO:0071383	cellular response to steroid hormone stimulus	1/17	243/17913	0.20729	0.24085	0.139789	2308	1
GO:0006310	GO:0006310	DNA recombination	1/17	244/17913	0.208052	0.24085	0.139789	3329	1
GO:0033673	GO:0033673	negative regulation of kinase activity	1/17	244/17913	0.208052	0.24085	0.139789	3315	1
GO:0045926	GO:0045926	negative regulation of growth	1/17	244/17913	0.208052	0.24085	0.139789	8835	1
GO:0051924	GO:0051924	regulation of calcium ion transport	1/17	244/17913	0.208052	0.24085	0.139789	581	1
GO:0009108	GO:0009108	coenzyme biosynthetic process	1/17	245/17913	0.208814	0.241498	0.140165	2194	1
GO:0021537	GO:0021537	telencephalon development	1/17	246/17913	0.209576	0.241911	0.140405	581	1
GO:0045017	GO:0045017	glycerolipid biosynthetic process	1/17	246/17913	0.209576	0.241911	0.140405	84649	1
GO:0008016	GO:0008016	regulation of heart contraction	1/17	249/17913	0.211855	0.244307	0.141796	4193	1
GO:0045637	GO:0045637	regulation of myeloid cell differentiation	1/17	250/17913	0.212614	0.24471	0.14203	4609	1
GO:0090596	GO:0090596	sensory organ morphogenesis	1/17	250/17913	0.212614	0.24471	0.14203	581	1
GO:0022409	GO:0022409	positive regulation of cell-cell adhesion	1/17	251/17913	0.213372	0.245111	0.142262	3329	1
GO:0090257	GO:0090257	regulation of muscle system process	1/17	251/17913	0.213372	0.245111	0.142262	2308	1
GO:0032868	GO:0032868	response to insulin	1/17	252/17913	0.214129	0.245745	0.14263	2308	1
GO:0007249	GO:0007249	I-kappaB kinase/NF-kappaB signaling	1/17	253/17913	0.214885	0.246141	0.14286	3315	1
GO:0060326	GO:0060326	cell chemotaxis	1/17	253/17913	0.214885	0.246141	0.14286	3315	1
GO:0034976	GO:0034976	response to endoplasmic reticulum stress	1/17	254/17913	0.215641	0.246771	0.143225	581	1
GO:0007204	GO:0007204	positive regulation of cytosolic calcium ion concentration	1/17	255/17913	0.216396	0.247399	0.14359	581	1
GO:0044839	GO:0044839	cell cycle G2/M phase transition	1/17	257/17913	0.217904	0.248885	0.144452	3320	1
GO:0051251	GO:0051251	positive regulation of lymphocyte activation	1/17	258/17913	0.218657	0.249507	0.144813	3329	1
GO:0060828	GO:0060828	regulation of canonical Wnt signaling pathway	1/17	264/17913	0.223161	0.254404	0.147656	2308	1
GO:0009152	GO:0009152	purine ribonucleotide biosynthetic process	1/17	266/17913	0.224657	0.255622	0.148363	2194	1
GO:0043270	GO:0043270	positive regulation of ion transport	1/17	266/17913	0.224657	0.255622	0.148363	581	1
GO:1902105	GO:1902105	regulation of leukocyte differentiation	1/17	267/17913	0.225404	0.256229	0.148715	4609	1
GO:0043491	GO:0043491	protein kinase B signaling	1/17	268/17913	0.22615	0.256589	0.148924	3320	1
GO:0090305	GO:0090305	nucleic acid phosphodiester bond hydrolysis	1/17	268/17913	0.22615	0.256589	0.148924	581	1
GO:0007548	GO:0007548	sex differentiation	1/17	269/17913	0.226896	0.257192	0.149274	581	1
GO:0050768	GO:0050768	negative regulation of neurogenesis	1/17	271/17913	0.228385	0.258635	0.150111	4193	1
GO:0051348	GO:0051348	negative regulation of transferase activity	1/17	272/17913	0.229129	0.259231	0.150457	3315	1
GO:0043542	GO:0043542	endothelial cell migration	1/17	275/17913	0.231355	0.26101	0.15149	3315	1

	ID	Description	Generation	Bg Ratio	*P* value	*P*. adjust	Q value	Gene ID	Count
GO:0060047	GO:0060047	heart contraction	1/17	275/17913	0.231355	0.26101	0.15149	4193	1
GO:1903311	GO:1903311	regulation of mRNA metabolic process	1/17	275/17913	0.231355	0.26101	0.15149	3315	1
GO:0043414	GO:0043414	macromolecule methylation	1/17	276/17913	0.232096	0.2616	0.151832	4609	1
GO:0034330	GO:0034330	cell junction organization	1/17	277/17913	0.232836	0.262187	0.152173	6624	1
GO:0009260	GO:0009260	ribonucleotide biosynthetic process	1/17	279/17913	0.234314	0.263604	0.152995	2194	1
GO:0006164	GO:0006164	purine nucleotide biosynthetic process	1/17	280/17913	0.235053	0.264186	0.153333	2194	1
GO:0003015	GO:0003015	heart process	1/17	284/17913	0.237998	0.266995	0.154964	4193	1
GO:0046390	GO:0046390	ribose phosphate biosynthetic process	1/17	284/17913	0.237998	0.266995	0.154964	2194	1
GO:0009895	GO:0009895	negative regulation of catabolic process	1/17	288/17913	0.240934	0.270035	0.156728	4194	1
GO:0051480	GO:0051480	regulation of cytosolic calcium ion concentration	1/17	289/17913	0.241666	0.27035	0.15691	581	1
GO:0070371	GO:0070371	ERK1 and ERK2 cascade	1/17	289/17913	0.241666	0.27035	0.15691	4609	1
GO:0051961	GO:0051961	negative regulation of nervous system development	1/17	290/17913	0.242397	0.270915	0.157238	4193	1
GO:0072522	GO:0072522	purine-containing compound biosynthetic process	1/17	291/17913	0.243128	0.271478	0.157565	2194	1
GO:1903522	GO:1903522	regulation of blood circulation	1/17	292/17913	0.243858	0.27204	0.157891	4193	1
GO:0002449	GO:0002449	lymphocyte mediated immunity	1/17	293/17913	0.244588	0.272346	0.158069	3329	1
GO:0070588	GO:0070588	calcium ion transmembrane transport	1/17	293/17913	0.244588	0.272346	0.158069	581	1
GO:0010632	GO:0010632	regulation of epithelial cell migration	1/17	294/17913	0.245317	0.2727	0.158274	3315	1
GO:0002460	GO:0002460	adaptive immune response based on somatic recombination of immune receptors built from immunoglobulin superfamily domains	1/17	295/17913	0.246045	0.2727	0.158274	3329	1
GO:0016485	GO:0016485	protein processing	1/17	295/17913	0.246045	0.2727	0.158274	4193	1
GO:0043010	GO:0043010	camera-type eye development	1/17	295/17913	0.246045	0.2727	0.158274	581	1
GO:1903037	GO:1903037	regulation of leukocyte cell-cell adhesion	1/17	295/17913	0.246045	0.2727	0.158274	3329	1
GO:0032956	GO:0032956	regulation of actin cytoskeleton organization	1/17	297/17913	0.247499	0.274058	0.159063	6624	1
GO:0002758	GO:0002758	innate immune response-activating signal transduction	1/17	298/17913	0.248225	0.274354	0.159235	3329	1
GO:0032984	GO:0032984	protein-containing complex disassembly	1/17	298/17913	0.248225	0.274354	0.159235	4609	1
GO:0006898	GO:0006898	receptor-mediated endocytosis	1/17	301/17913	0.2504	0.276503	0.160482	3320	1
GO:0006913	GO:0006913	nucleocytoplasmic transport	1/17	302/17913	0.251124	0.277046	0.160797	4193	1
GO:0002696	GO:0002696	positive regulation of leukocyte activation	1/17	305/17913	0.25329	0.278922	0.161886	3329	1
GO:0051169	GO:0051169	nuclear transport	1/17	305/17913	0.25329	0.278922	0.161886	4193	1
GO:0050863	GO:0050863	regulation of T cell activation	1/17	307/17913	0.254731	0.280251	0.162657	3329	1

Supplementary Table S2 Continued

	ID	Description	Generation	Bg Ratio	*P* value	*P*. adjust	Q value	Gene ID	Count
GO:0006909	GO:0006909	phagocytosis	1/17	308/17913	0.255451	0.280785	0.162967	3320	1
GO:0051188	GO:0051188	cofactor biosynthetic process	1/17	310/17913	0.256888	0.282106	0.163734	2194	1
GO:0033044	GO:0033044	regulation of chromosome organization	1/17	311/17913	0.257606	0.282376	0.16389	4609	1
GO:0060070	GO:0060070	canonical Wnt signaling pathway	1/17	311/17913	0.257606	0.282376	0.16389	2308	1
GO:1904062	GO:1904062	regulation of cation transmembrane transport	1/17	312/17913	0.258323	0.282903	0.164196	581	1
GO:0060562	GO:0060562	epithelial tube morphogenesis	1/17	313/17913	0.259039	0.283169	0.164351	4609	1
GO:1901214	GO:1901214	regulation of neuron death	1/17	313/17913	0.259039	0.283169	0.164351	581	1
GO:0032103	GO:0032103	positive regulation of response to external stimulus	1/17	315/17913	0.26047	0.284473	0.165108	3315	1
GO:0010721	GO:0010721	negative regulation of cell development	1/17	317/17913	0.261898	0.285772	0.165862	4193	1
GO:0072330	GO:0072330	monocarboxylic acid biosynthetic process	1/17	318/17913	0.262611	0.286029	0.166011	2194	1
GO:1903829	GO:1903829	positive regulation of cellular protein localization	1/17	318/17913	0.262611	0.286029	0.166011	4193	1
GO:0002218	GO:0002218	activation of innate immune response	1/17	319/17913	0.263324	0.286284	0.166159	3329	1
GO:0050867	GO:0050867	positive regulation of cell activation	1/17	319/17913	0.263324	0.286284	0.166159	3329	1
GO:0001505	GO:0001505	regulation of neurotransmitter levels	1/17	321/17913	0.264747	0.28757	0.166905	3320	1
GO:0019058	GO:0019058	viral life cycle	1/17	324/17913	0.266876	0.28934	0.167932	8519	1
GO:0045861	GO:0045861	negative regulation of proteolysis	1/17	324/17913	0.266876	0.28934	0.167932	4193	1
GO:0007015	GO:0007015	actin filament organization	1/17	325/17913	0.267585	0.28934	0.167932	6624	1
GO:0030098	GO:0030098	lymphocyte differentiation	1/17	325/17913	0.267585	0.28934	0.167932	581	1
GO:0048638	GO:0048638	regulation of developmental growth	1/17	325/17913	0.267585	0.28934	0.167932	8835	1
GO:0022412	GO:0022412	cellular process involved in reproduction in multicellular organism	1/17	326/17913	0.268293	0.289843	0.168225	581	1
GO:0007159	GO:0007159	leukocyte cell-cell adhesion	1/17	328/17913	0.269707	0.290846	0.168807	3329	1
GO:0043161	GO:0043161	proteasome-mediated ubiquitin-dependent protein catabolic process	1/17	328/17913	0.269707	0.290846	0.168807	4193	1
GO:0007596	GO:0007596	blood coagulation	1/17	331/17913	0.271823	0.292864	0.169978	3315	1
GO:0030336	GO:0030336	negative regulation of cell migration	1/17	332/17913	0.272527	0.293359	0.170265	8519	1
GO:0030198	GO:0030198	extracellular matrix organization	1/17	334/17913	0.273933	0.294607	0.17099	6624	1
GO:0010038	GO:0010038	response to metal ion	1/17	335/17913	0.274635	0.294833	0.17112	4193	1
GO:0060271	GO:0060271	cilium assembly	1/17	335/17913	0.274635	0.294833	0.17112	3320	1
GO:0007599	GO:0007599	hemostasis	1/17	336/17913	0.275337	0.295057	0.17125	3315	1

	ID	Description	Generation	Bg Ratio	*P* value	*P*. adjust	Q value	Gene ID	Count
GO:0032259	GO:0032259	methylation	1/17	336/17913	0.275337	0.295057	0.17125	4609	1
GO:0050817	GO:0050817	coagulation	1/17	337/17913	0.276038	0.295543	0.171533	3315	1
GO:0006402	GO:0006402	mRNA catabolic process	1/17	338/17913	0.276738	0.296028	0.171814	3315	1
GO:0030111	GO:0030111	regulation of Wnt signaling pathway	1/17	341/17913	0.278835	0.297739	0.172807	2308	1
GO:0032970	GO:0032970	regulation of actin filament-based process	1/17	341/17913	0.278835	0.297739	0.172807	6624	1
GO:0001654	GO:0001654	eye development	1/17	342/17913	0.279533	0.298218	0.173085	581	1
GO:0044782	GO:0044782	cilium organization	1/17	346/17913	0.282317	0.300652	0.174498	3320	1
GO:0150063	GO:0150063	visual system development	1/17	346/17913	0.282317	0.300652	0.174498	581	1
GO:0070997	GO:0070997	neuron death	1/17	347/17913	0.283011	0.300857	0.174617	581	1
GO:2000146	GO:2000146	negative regulation of cell motility	1/17	347/17913	0.283011	0.300857	0.174617	8519	1
GO:0009123	GO:0009123	nucleoside monophosphate metabolic process	1/17	349/17913	0.284399	0.302063	0.175317	7083	1
GO:0048880	GO:0048880	sensory system development	1/17	351/17913	0.285783	0.303265	0.176014	581	1
GO:0010631	GO:0010631	epithelial cell migration	1/17	354/17913	0.287855	0.305193	0.177134	3315	1
GO:0045666	GO:0045666	positive regulation of neuron differentiation	1/17	355/17913	0.288545	0.305653	0.177401	8835	1
GO:0090132	GO:0090132	epithelium migration	1/17	357/17913	0.289922	0.30684	0.17809	3315	1
GO:0009165	GO:0009165	nucleotide biosynthetic process	1/17	360/17913	0.291983	0.308476	0.179039	2194	1
GO:0043687	GO:0043687	post-translational protein modification	1/17	360/17913	0.291983	0.308476	0.179039	8835	1
GO:0031346	GO:0031346	positive regulation of cell projection organization	1/17	362/17913	0.293354	0.309651	0.179721	6624	1
GO:0090130	GO:0090130	tissue migration	1/17	363/17913	0.294038	0.3101	0.179982	3315	1
GO:0006605	GO:0006605	protein targeting	1/17	364/17913	0.294722	0.310548	0.180241	3320	1
GO:0010959	GO:0010959	regulation of metal ion transport	1/17	365/17913	0.295405	0.310721	0.180342	581	1
GO:0051271	GO:0051271	negative regulation of cellular component movement	1/17	365/17913	0.295405	0.310721	0.180342	8519	1
GO:0010876	GO:0010876	lipid localization	1/17	366/17913	0.296088	0.311165	0.1806	84649	1
GO:0006401	GO:0006401	RNA catabolic process	1/17	368/17913	0.297451	0.31205	0.181113	3315	1
GO:0051098	GO:0051098	regulation of binding	1/17	368/17913	0.297451	0.31205	0.181113	581	1
GO:0030900	GO:0030900	forebrain development	1/17	371/17913	0.299491	0.313915	0.182196	581	1
GO:0045089	GO:0045089	positive regulation of innate immune response	1/17	373/17913	0.300848	0.315062	0.182861	3329	1
GO:0042391	GO:0042391	regulation of membrane potential	1/17	374/17913	0.301526	0.315495	0.183113	581	1
GO:0006874	GO:0006874	cellular calcium ion homeostasis	1/17	375/17913	0.302203	0.315928	0.183364	581	1
GO:0018205	GO:0018205	peptidyl-lysine modification	1/17	376/17913	0.302879	0.316358	0.183614	4193	1

Supplementary Table S2 Continued

	ID	Description	Generation	Bg Ratio	*P* value	*P*. adjust	Q value	Gene ID	Count
GO:0040013	GO:0040013	negative regulation of locomotion	1/17	377/17913	0.303555	0.316788	0.183863	8519	1
GO:0022613	GO:0022613	ribonucleoprotein complex biogenesis	1/17	381/17913	0.306252	0.319324	0.185335	3320	1
GO:0010498	GO:0010498	proteasomal protein catabolic process	1/17	382/17913	0.306925	0.319747	0.185581	4193	1
GO:0045765	GO:0045765	regulation of angiogenesis	1/17	383/17913	0.307597	0.320168	0.185825	3315	1
GO:0043062	GO:0043062	extracellular structure organization	1/17	387/17913	0.310279	0.32268	0.187283	6624	1
GO:0045785	GO:0045785	positive regulation of cell adhesion	1/17	388/17913	0.310948	0.322814	0.187361	3329	1
GO:0055074	GO:0055074	calcium ion homeostasis	1/17	388/17913	0.310948	0.322814	0.187361	581	1
GO:0022407	GO:0022407	regulation of cell-cell adhesion	1/17	389/17913	0.311617	0.323228	0.187601	3329	1
GO:0072503	GO:0072503	cellular divalent inorganic cation homeostasis	1/17	392/17913	0.313618	0.325022	0.188643	581	1
GO:0007409	GO:0007409	axonogenesis	1/17	393/17913	0.314284	0.325431	0.18888	3320	1
GO:0001558	GO:0001558	regulation of cell growth	1/17	396/17913	0.316278	0.327213	0.189914	8835	1
GO:0006417	GO:0006417	regulation of translation	1/17	397/17913	0.316942	0.327616	0.190148	3315	1
GO:0032386	GO:0032386	regulation of intracellular transport	1/17	400/17913	0.318929	0.329386	0.191175	4193	1
GO:0051249	GO:0051249	regulation of lymphocyte activation	1/17	401/17913	0.31959	0.329784	0.191406	3329	1
GO:0006644	GO:0006644	phospholipid metabolic process	1/17	402/17913	0.320251	0.330181	0.191636	8835	1
GO:0051222	GO:0051222	positive regulation of protein transport	1/17	403/17913	0.320911	0.330576	0.191866	4193	1
GO:0043900	GO:0043900	regulation of multi-organism process	1/17	408/17913	0.324201	0.333679	0.193667	8519	1
GO:0072507	GO:0072507	divalent inorganic cation homeostasis	1/17	412/17913	0.326823	0.336088	0.195065	581	1
GO:0006816	GO:0006816	calcium ion transport	1/17	415/17913	0.328783	0.337814	0.196066	581	1
GO:0051346	GO:0051346	negative regulation of hydrolase activity	1/17	420/17913	0.332037	0.340865	0.197837	4193	1
GO:1901342	GO:1901342	regulation of vasculature development	1/17	425/17913	0.335277	0.343896	0.199597	3315	1
GO:0046394	GO:0046394	carboxylic acid biosynthetic process	1/17	426/17913	0.335923	0.343969	0.199639	2194	1
GO:0048608	GO:0048608	reproductive structure development	1/17	426/17913	0.335923	0.343969	0.199639	581	1
GO:0016053	GO:0016053	organic acid biosynthetic process	1/17	427/17913	0.336569	0.344336	0.199852	2194	1
GO:0061458	GO:0061458	reproductive system development	1/17	429/17913	0.337858	0.34536	0.200446	581	1
GO:0015711	GO:0015711	organic anion transport	1/17	430/17913	0.338502	0.345723	0.200657	2538	1
GO:0061564	GO:0061564	axon development	1/17	438/17913	0.343631	0.350662	0.203524	3320	1
GO:0045088	GO:0045088	regulation of innate immune response	1/17	440/17913	0.344908	0.351366	0.203932	3329	1
GO:1904951	GO:1904951	positive regulation of establishment of protein localization	1/17	440/17913	0.344908	0.351366	0.203932	4193	1
GO:0002697	GO:0002697	regulation of immune effector process	1/17	441/17913	0.345545	0.351716	0.204135	3329	1
GO:0001667	GO:0001667	ameboidal-type cell migration	1/17	446/17913	0.348723	0.354649	0.205838	3315	1

	ID	Description	Generation	Bg Ratio	*P* value	*P*. adjust	Q value	Gene ID	Count
GO:0003012	GO:0003012	muscle system process	1/17	448/17913	0.34999	0.355635	0.20641	2308	1
GO:0002683	GO:0002683	negative regulation of immune system process	1/17	451/17913	0.351887	0.357259	0.207353	4609	1
GO:0034248	GO:0034248	regulation of cellular amide metabolic process	1/17	452/17913	0.352518	0.357596	0.207548	3315	1
GO:0034765	GO:0034765	regulation of ion transmembrane transport	1/17	453/17913	0.353148	0.357932	0.207743	581	1
GO:0070838	GO:0070838	divalent metal ion transport	1/17	454/17913	0.353778	0.358267	0.207938	581	1
GO:0050769	GO:0050769	positive regulation of neurogenesis	1/17	455/17913	0.354407	0.358601	0.208132	8835	1
GO:0010975	GO:0010975	regulation of neuron projection development	1/17	458/17913	0.356291	0.359596	0.208709	4193	1
GO:0016311	GO:0016311	dephosphorylation	1/17	458/17913	0.356291	0.359596	0.208709	2538	1
GO:0072511	GO:0072511	divalent inorganic cation transport	1/17	458/17913	0.356291	0.359596	0.208709	581	1
GO:1903706	GO:1903706	regulation of hemopoiesis	1/17	468/17913	0.362535	0.365589	0.212187	4609	1
GO:0051493	GO:0051493	regulation of cytoskeleton organization	1/17	472/17913	0.365016	0.367781	0.21346	6624	1
GO:0071900	GO:0071900	regulation of protein serine/threonine kinase activity	1/17	479/17913	0.369337	0.371822	0.215805	3315	1
GO:0043312	GO:0043312	neutrophil degranulation	1/17	485/17913	0.373018	0.375212	0.217773	3320	1
GO:0002283	GO:0002283	neutrophil activation involved in immune response	1/17	488/17913	0.374851	0.37674	0.218659	3320	1
GO:0002694	GO:0002694	regulation of leukocyte activation	1/17	492/17913	0.377287	0.37887	0.219896	3329	1
GO:0016055	GO:0016055	Wnt signaling pathway	1/17	498/17913	0.380925	0.382133	0.221789	2308	1
GO:0002446	GO:0002446	neutrophil mediated immunity	1/17	499/17913	0.381529	0.382133	0.221789	3320	1
GO:0042119	GO:0042119	neutrophil activation	1/17	499/17913	0.381529	0.382133	0.221789	3320	1
GO:0048667	GO:0048667	cell morphogenesis involved in neuron differentiation	1/17	500/17913	0.382133	0.382133	0.221789	3320	1
GO:0198738	GO:0198738	cell-cell signaling by wnt	1/17	500/17913	0.382133	0.382133	0.221789	2308	1

Supplementary Table S3 Metabolism associated gene sets from MsigDB

Classification	Pathways
carbohydrates metabolism	KEGG_FRUCTOSE_AND_MANNOSE_METABOLISM
carbohydrates metabolism	KEGG_GALACTOSE_METABOLISM
carbohydrates metabolism	KEGG_STARCH_AND_SUCROSE_METABOLISM
carbohydrates metabolism	KEGG_PYRUVATE_METABOLISM
carbohydrates metabolism	KEGG_GLYOXYLATE_AND_DICARBOXYLATE_METABOLISM
carbohydrates metabolism	KEGG_OTHER_GLYCAN_DEGRADATION
carbohydrates metabolism	REACTOME_GLUCOSE_METABOLISM
carbohydrates metabolism	REACTOME_GLYOXYLATE_METABOLISM_AND_GLYCINE_DEGRADATION
lipid metabolism	KEGG_FATTY_ACID_METABOLISM
lipid metabolism	KEGG_ETHER_LIPID_METABOLISM
lipid metabolism	KEGG_STEROID_HORMONE_BIOSYNTHESIS
lipid metabolism	KEGG_BIOSYNTHESIS_OF_UNSATURATED_FATTY_ACIDS
lipid metabolism	REACTOME_TRIGLYCERIDE_BIOSYNTHESIS
lipid metabolism	REACTOME_FATTY_ACYL_COA_BIOSYNTHESIS
lipid metabolism	REACTOME_CHOLESTEROL_BIOSYNTHESIS
lipid metabolism	REACTOME_REGULATION_OF_CHOLESTEROL_BIOSYNTHESIS_BY_SREBP_SREBF
lipid metabolism	REACTOME_MITOCHONDRIAL_FATTY_ACID_BETA_OXIDATION
lipid metabolism	REACTOME_SYNTHESIS_OF_VERY_LONG_CHAIN_FATTY_ACYL_COAS
lipid metabolism	REACTOME_BETA_OXIDATION_OF_VERY_LONG_CHAIN_FATTY_ACIDS
lipid metabolism	REACTOME_MITOCHONDRIAL_FATTY_ACID_BETA_OXIDATION_OF_SATURATED_FATTY_ACIDS
lipid metabolism	REACTOME_MITOCHONDRIAL_FATTY_ACID_BETA_OXIDATION_OF_UNSATURATED_FATTY_ACIDS
lipid metabolism	REACTOME_TRANSPORT_OF_FATTY_ACIDS
lipid metabolism	REACTOME_PEROXISOMAL_LIPID_METABOLISM
lipid metabolism	REACTOME_KETONE_BODY_METABOLISM
lipid metabolism	REACTOME_TRIGLYCERIDE_METABOLISM
amino acid metabolism	KEGG_GLYCINE_SERINE_AND_THREONINE_METABOLISM
amino acid metabolism	KEGG_CYSTEINE_AND_METHIONINE_METABOLISM
amino acid metabolism	KEGG_ARGININE_AND_PROLINE_METABOLISM
amino acid metabolism	KEGG_HISTIDINE_METABOLISM
amino acid metabolism	KEGG_TYROSINE_METABOLISM
amino acid metabolism	KEGG_PHENYLALANINE_METABOLISM
amino acid metabolism	KEGG_TRYPTOPHAN_METABOLISM
amino acid metabolism	KEGG_BETA_ALANINE_METABOLISM
amino acid metabolism	KEGG_TAURINE_AND_HYPOTAURINE_METABOLISM
amino acid metabolism	KEGG_SELENOAMINO_ACID_METABOLISM
amino acid metabolism	KEGG_GLUTATHIONE_METABOLISM
amino acid metabolism	KEGG_VALINE_LEUCINE_AND_ISOLEUCINE_DEGRADATION
amino acid metabolism	KEGG_LYSINE_DEGRADATION
amino acid metabolism	REACTOME_METABOLISM_OF_AMINO_ACIDS_AND_DERIVATIVES
amino acid metabolism	KEGG_PURINE_METABOLISM
amino acid metabolism	KEGG_PYRIMIDINE_METABOLISM
other metabolism	KEGG_ALANINE_ASPARTATE_AND_GLUTAMATE_METABOLISM
other metabolism	KEGG_INOSITOL_PHOSPHATE_METABOLISM
other metabolism	KEGG_ARACHIDONIC_ACID_METABOLISM

Supplementary Table S3 Continued

Classification	Pathways
other metabolism	KEGG_LINOLEIC_ACID_METABOLISM
other metabolism	KEGG_ALPHA_LINOLENIC_ACID_METABOLISM
other metabolism	REACTOME_GLYCOSAMINOGLYCAN_METABOLISM
other metabolism	REACTOME_ASPARTATE_AND_ASPARAGINE_METABOLISM
other metabolism	REACTOME_GLUTAMATE_AND_GLUTAMINE_METABOLISM

Supplementary Table S4 Univariate Cox analysis of DEGs among 3 m6Aclusters

Gene	HR	lower_95	upper_95	*P*
DHX9	1.633843	1.265992	2.108577	0.000162
ARL6IP6	1.754889	1.370438	2.247189	8.28E-06
CPSF6	1.977523	1.50328	2.601377	1.09E-06
KPNB1	1.84941	1.446524	2.364507	9.35E-07
CBX1	1.645566	1.341473	2.018593	1.77E-06
SMARCC1	1.78726	1.390534	2.297173	5.78E-06
ADNP	1.460464	1.133426	1.881866	0.003409
TOPBP1	1.763756	1.383423	2.248651	4.67E-06
ACTL6A	1.783425	1.422593	2.235779	5.27E-07
MSH2	1.85358	1.48736	2.309972	3.91E-08
NUP205	2.080123	1.597189	2.709078	5.51E-08
DCAF7	1.659214	1.308409	2.104077	2.94E-05
UBA2	1.716606	1.348014	2.185983	1.18E-05
ILF3	1.585569	1.225688	2.051117	0.000449
RBBP4	1.808016	1.420093	2.301906	1.54E-06
INCENP	1.788071	1.431509	2.233446	3.04E-07
USP1	1.676229	1.346374	2.086899	3.84E-06
MAPRE1	1.535861	1.261389	1.870057	1.94E-05
MAPK1	1.54419	1.200883	1.98564	0.000707
DCAF16	1.478802	1.186783	1.842674	0.000491
UBAP2L	1.634528	1.298929	2.056835	2.78E-05
FUBP1	1.628169	1.29538	2.046451	2.94E-05
PRRC2A	1.388239	1.120059	1.720631	0.002742
RCC2	1.463681	1.232288	1.738524	1.43E-05
RACGAP1	1.679514	1.415363	1.992962	2.87E-09
HIATL1	1.485514	1.179595	1.87077	0.000769
IARS	1.756585	1.393032	2.215018	1.92E-06
ECT2	1.577703	1.357705	1.833347	2.67E-09
NCAPD2	1.713872	1.431721	2.051627	4.35E-09
PRRC2C	1.353676	1.083277	1.691571	0.007732
SMC3	1.300787	1.063746	1.59065	0.010406
WHSC1	1.707651	1.352782	2.155612	6.73E-06
SPTLC1	1.291114	1.027296	1.622683	0.02846
ANLN	1.759062	1.510092	2.04908	4.06E-13
POGK	1.556567	1.25966	1.923458	4.17E-05
SMC4	1.471375	1.237075	1.750052	1.28E-05
SPATS2	1.866591	1.476124	2.360346	1.87E-07
BUB1B	1.712282	1.445926	2.027704	4.53E-10
TMEM194A	1.738784	1.403186	2.154648	4.28E-07
FNBP1L	1.302398	1.06712	1.589549	0.009349
KIF5B	1.375641	1.097724	1.723921	0.005611

Continued

Gene	HR	lower_95	upper_95	*P*
VHL	1.571319	1.239593	1.991819	0.000188
ABL1	1.343296	1.064797	1.694637	0.012789
CAD	1.985547	1.589615	2.480094	1.50E-09
ACLY	1.599402	1.280129	1.998305	3.57E-05
HMGN4	1.274654	1.069139	1.519673	0.006826
LMNB2	1.604253	1.36277	1.888526	1.36E-08
CORO1C	1.757719	1.409408	2.192109	5.57E-07
FAM168B	1.40641	1.114791	1.774312	0.004021
CDK2	1.685099	1.367181	2.076944	9.99E-07
NCAPG2	1.858395	1.507963	2.290262	6.14E-09
SPIN1	1.167303	0.952239	1.430939	0.136506
YWHAQ	1.849184	1.493894	2.288973	1.63E-08
NCAPH	1.764117	1.468488	2.11926	1.31E-09
DYNC1I2	1.427989	1.146756	1.778192	0.001454
CENPF	1.551853	1.326213	1.815882	4.21E-08
DCK	1.580328	1.271727	1.963816	3.65E-05
KIF23	1.775681	1.49252	2.112563	9.28E-11
MCM3	1.463432	1.248493	1.715374	2.62E-06
PIP5K1A	1.219548	0.975399	1.524808	0.08161
DNMT1	1.490359	1.228024	1.808734	5.36E-05
RNF44	1.406288	1.118304	1.768434	0.003541
ZBTB12	1.389537	1.133699	1.703108	0.001532
FAM60A	1.352516	1.136631	1.609405	0.000666
TCF3	1.466672	1.195646	1.799134	0.000239
TUBA1B	1.440566	1.237805	1.67654	2.40E-06
KIAA0907	1.39708	1.12448	1.735765	0.002534
MCM6	1.673566	1.41769	1.975625	1.18E-09
KIF11	1.672672	1.388653	2.014782	6.02E-08
TAF6	1.617737	1.290274	2.028309	3.07E-05
PRC1	1.551915	1.340139	1.797158	4.33E-09
NDRG3	1.65889	1.348627	2.040532	1.66E-06
RAD51AP1	1.663509	1.387695	1.994143	3.75E-08
WASF2	1.620405	1.297307	2.023972	2.10E-05
PAPSS1	1.392953	1.137788	1.705342	0.001326
TOP2A	1.402754	1.247856	1.57688	1.44E-08
LDB1	1.073609	0.870988	1.323367	0.505681
MCM2	1.52158	1.329472	1.741448	1.09E-09
PRRC2B	1.201654	0.958844	1.505953	0.110702
BUB1	1.862314	1.551645	2.235184	2.42E-11
LAMC1	1.209622	1.054453	1.387625	0.006589
SLC39A6	1.440792	1.201733	1.727406	7.97E-05
CDK4	1.817074	1.511922	2.183815	1.93E-10

Supplementary Table S4 Continued

Gene	HR	lower_95	upper_95	*P*
PRKDC	1.554749	1.276924	1.893022	1.11E-05
RAD21	1.487258	1.214455	1.821342	0.000123
C11orf84	1.66982	1.405931	1.98324	5.16E-09
MELK	1.656179	1.416141	1.936904	2.70E-10
CEP55	1.684272	1.447183	1.960203	1.64E-11
MCM4	1.517572	1.298477	1.773634	1.58E-07
CALU	1.631187	1.354868	1.963861	2.38E-07
GINS1	1.708585	1.450781	2.012201	1.37E-10
TRIP13	1.760912	1.518016	2.042673	7.92E-14
ARHGAP11A	1.729447	1.415534	2.112975	8.30E-08
CDCA8	1.780715	1.534029	2.067071	3.35E-14
MPZL1	1.576393	1.313693	1.891626	9.91E-07
PIGS	1.506544	1.24971	1.816161	1.73E-05
GTSE1	1.749898	1.46316	2.092827	8.88E-10
CKAP2	1.653488	1.356176	2.015979	6.61E-07
FAM49B	1.502273	1.237696	1.823408	3.83E-05
RAN	2.006556	1.6253	2.477244	9.33E-11
DEK	1.380363	1.13992	1.671522	0.000963
ASAP1	1.44473	1.196327	1.744711	0.000132
MAD2L1	1.758154	1.472401	2.099364	4.51E-10
XPOT	1.610708	1.31571	1.971847	3.87E-06
PNMA1	1.336918	1.14364	1.562861	0.000268
NUF2	1.614842	1.391461	1.874084	2.81E-10
PLXNA1	1.462333	1.215666	1.759051	5.53E-05
CDC42SE1	1.174922	0.957516	1.441689	0.122558
PLK1	1.667485	1.441089	1.929448	6.51E-12
LMNB1	1.551774	1.333873	1.80527	1.26E-08
RRM1	1.66651	1.346517	2.062549	2.67E-06
M6PR	1.412273	1.154492	1.727612	0.000788
PRR11	1.945132	1.631461	2.319111	1.21E-13
DLGAP5	1.775231	1.500642	2.100064	2.17E-11
NAP1L1	1.662887	1.374369	2.011973	1.69E-07
KIF20A	1.787953	1.521089	2.101635	1.85E-12
ZMIZ1	1.205179	0.983802	1.476369	0.071504
BCL9L	1.238959	1.020293	1.504488	0.030561
FOXM1	1.594791	1.383762	1.838002	1.16E-10
MKI67	1.56225	1.341144	1.819809	1.01E-08
YAP1	1.05967	0.869417	1.291555	0.565949
KIF2C	1.732937	1.500855	2.000906	6.64E-14
RNF145	1.612289	1.342552	1.936221	3.16E-07
NEK2	1.598953	1.37634	1.857572	8.47E-10
NCAPG	1.848446	1.551723	2.201909	5.93E-12

Gene	HR	lower_95	upper_95	*P*
BCL9	1.335075	1.102703	1.616413	0.003056
ZNF189	1.198932	0.980287	1.466345	0.077371
KIFC1	1.541013	1.345242	1.765273	4.42E-10
ADAM9	1.414167	1.224773	1.632849	2.32E-06
CLSTN1	1.253416	1.088075	1.443882	0.001751
TPX2	1.603356	1.404697	1.83011	2.65E-12
UHRF1	1.548191	1.314151	1.823911	1.72E-07
NUSAP1	1.398369	1.21635	1.607625	2.44E-06
CHAF1B	1.567346	1.310412	1.874659	8.68E-07
KIF4A	1.658472	1.430165	1.923225	2.16E-11
CENPA	1.853751	1.579586	2.175503	4.08E-14
EZH2	1.855253	1.532051	2.246637	2.48E-10
CDK1	1.579566	1.369221	1.822224	3.62E-10
DTL	1.532323	1.299989	1.80618	3.63E-07
TFDP1	1.486999	1.25143	1.766912	6.52E-06
RFC4	1.650568	1.384196	1.968201	2.40E-08
WASF1	1.535861	1.266361	1.862715	1.31E-05
SNHG1	1.375912	1.13527	1.667564	0.00114
CCNB2	1.460051	1.267259	1.682173	1.62E-07
KPNA2	1.849156	1.582088	2.161306	1.13E-14
SLC27A5	0.842209	0.779709	0.909719	1.27E-05
DDX21	1.353586	1.114414	1.644089	0.002273
DLG5	1.308062	1.08809	1.572505	0.004255
CDC6	1.532233	1.328385	1.767362	4.67E-09
C12orf49	1.394071	1.167923	1.664007	0.000234
APOC1	0.835347	0.772169	0.903695	7.34E-06
LIMK1	1.355673	1.147746	1.601269	0.000341
HPX	0.857421	0.807456	0.910476	5.12E-07
ZWINT	1.520355	1.322088	1.748354	4.19E-09
LRIG3	1.236273	1.040952	1.468243	0.015632
CDCA5	1.603028	1.378675	1.863889	8.55E-10
HMGB2	1.435482	1.232381	1.672055	3.41E-06
ASF1B	1.4721	1.281271	1.691351	4.79E-08
HP	0.91731	0.868921	0.968393	0.001799
STMN1	1.515771	1.317808	1.743473	5.72E-09
TUBA1C	1.631845	1.382482	1.926186	7.13E-09
APOC3	0.908037	0.86139	0.95721	0.000337
B4GALT5	1.50409	1.249558	1.810471	1.59E-05
ZNF468	1.288008	1.07715	1.540143	0.005525
HMGA1	1.397174	1.242442	1.571175	2.34E-08
MMD	1.419927	1.220372	1.652114	5.70E-06
ITGB1	1.203863	1.030198	1.406803	0.019582

Supplementary Table S4 Continued

Gene	HR	lower_95	upper_95	*P*
SRC	1.276877	1.112133	1.466025	0.000525
ARHGEF2	1.295457	1.10864	1.513755	0.001122
HRSP12	0.814514	0.732478	0.905739	0.000152
CDCA7L	1.422539	1.183944	1.709216	0.000168
ATP1A1	1.343783	1.165298	1.549605	4.83E-05
MYBL2	1.401593	1.272114	1.544251	8.69E-12
MAT2A	1.379937	1.141645	1.667966	0.00087
HIF1A	1.219053	1.045867	1.420917	0.01129
ANG	0.846513	0.775646	0.923855	0.000187
MCM5	1.549858	1.304835	1.840892	6.02E-07
CCNB1	1.613814	1.401719	1.858001	2.79E-11
UBE2T	1.539781	1.323752	1.791064	2.19E-08
FEN1	1.629316	1.378264	1.926097	1.08E-08
CDC45	1.640992	1.397889	1.926372	1.41E-09
MCM7	1.484774	1.274881	1.729222	3.71E-07
ASPM	1.555121	1.309383	1.846978	4.86E-07
TYMS	1.424967	1.242324	1.63446	4.18E-07
NDC80	1.840448	1.545947	2.19105	7.05E-12
TACC3	1.548105	1.326755	1.806385	2.83E-08
PCNA	1.545459	1.286399	1.856688	3.31E-06
FBP1	0.865606	0.794685	0.942857	0.000936
YWHAZ	1.556322	1.302671	1.859363	1.10E-06
AGXT	0.895711	0.842891	0.951842	0.000383
RBP4	0.879879	0.826195	0.937051	6.77E-05
PAQR4	1.381297	1.19441	1.597427	1.33E-05
H2AFZ	1.650859	1.392365	1.957343	7.94E-09
TCF19	1.392544	1.216133	1.594545	1.66E-06
SERPING1	0.714615	0.629007	0.811875	2.45E-07
ENAH	1.365585	1.150387	1.621039	0.000369
SLC10A1	0.859954	0.810606	0.912306	5.62E-07
MASP2	0.866072	0.806888	0.929597	6.85E-05
HPR	0.905498	0.848952	0.96581	0.00255
KIAA0101	1.544996	1.329159	1.795881	1.46E-08
NFE2L3	1.326811	1.123929	1.566314	0.000838
RNASE4	0.776159	0.670696	0.898207	0.000672
ITIH4	0.904748	0.810817	1.009562	0.073485
ATP1B3	1.450811	1.2677	1.660371	6.45E-08
APOF	0.891954	0.827449	0.961488	0.002832
HMMR	1.794553	1.50978	2.133041	3.29E-11
KLKB1	0.796452	0.713481	0.889073	5.02E-05
STX3	1.439407	1.207015	1.716542	5.03E-05
G6PD	1.418832	1.28654	1.564726	2.46E-12

Gene	HR	lower_95	upper_95	*P*
CDC20	1.47768	1.325913	1.646819	1.64E-12
TTLL4	1.403225	1.194051	1.649042	3.90E-05
PPT1	1.508137	1.260416	1.804545	7.19E-06
AZGP1	0.88558	0.819575	0.9569	0.002107
BAK1	1.482516	1.261594	1.742123	1.73E-06
SERPINC1	0.913554	0.867502	0.96205	0.000613
ALDOB	0.905812	0.858559	0.955665	0.000296
TROAP	1.556247	1.345224	1.800373	2.70E-09
MARCKS	1.3481	1.162523	1.563303	7.72E-05
F9	0.88688	0.83209	0.945278	0.000225
GLYATL1	0.808054	0.740597	0.881655	1.65E-06
CFB	0.844365	0.763143	0.934233	0.001044
ADD3	1.177362	1.003749	1.381005	0.044863
UBE2C	1.415857	1.267166	1.581996	8.11E-10
ITPR3	1.216795	1.066315	1.388512	0.003577
IQGAP1	1.129354	0.958058	1.331276	0.147217
NCK2	1.16119	1.024062	1.31668	0.019764
CD14	0.830675	0.737834	0.935199	0.002156
C8B	0.853145	0.790616	0.920619	4.32E-05
LAMB1	1.239264	1.095771	1.401549	0.000634
SAA4	0.894363	0.838306	0.954169	0.000723
TMCO3	1.474943	1.261576	1.724396	1.09E-06
TMEM51	1.125179	0.984373	1.286127	0.083798
HPD	0.932254	0.888051	0.978658	0.004649
TACC1	0.926582	0.778079	1.103428	0.392215
SOX4	1.191935	1.068108	1.330118	0.001705
ACSM2A	0.840948	0.777879	0.909131	1.33E-05
CCNA2	1.467484	1.288077	1.67188	8.17E-09
SCRN1	1.229557	1.071033	1.411544	0.003342
CKAP4	1.460279	1.249682	1.706366	1.89E-06
PON1	0.848762	0.796341	0.904634	4.63E-07
TMEM132A	1.146459	1.019126	1.2897	0.022884
NRM	1.262782	1.098071	1.452201	0.001068
KIAA1522	1.352749	1.159227	1.578579	0.000125
GPT	0.839287	0.758723	0.928406	0.000667
WWTR1	1.074538	0.907211	1.272725	0.405176
TTYH3	1.265091	1.091407	1.466414	0.001803
CLIC1	1.39655	1.220001	1.598646	1.27E-06
TAT	0.891911	0.84551	0.940858	2.71E-05
CFHR2	0.864606	0.796296	0.938775	0.000531
ITGAV	1.233429	1.073535	1.417139	0.00306
TPM4	1.147008	0.999362	1.316467	0.051071

Supplementary Table S4 Continued

Gene	HR	lower_95	upper_95	*P*
PTPLB	1.232869	1.063315	1.42946	0.00555
LDHD	0.776193	0.688626	0.874895	3.35E-05
CDT1	1.557378	1.355341	1.789532	4.14E-10
DSG2	1.252624	1.120976	1.399733	7.02E-05
HRG	0.8974	0.854781	0.942144	1.30E-05
MMP14	1.187165	1.064149	1.324401	0.002112
IGF2BP3	1.583305	1.36811	1.832349	7.04E-10
BMF	1.201639	1.042513	1.385053	0.011264
NR1I3	0.893837	0.813671	0.981901	0.019235
NCEH1	1.157625	1.006204	1.331832	0.040713
SLC22A1	0.852555	0.804222	0.903792	8.46E-08
PSPH	1.470569	1.258069	1.718961	1.28E-06
SNHG3	1.430073	1.222728	1.672579	7.60E-06
DCXR	0.86176	0.781842	0.949848	0.002734
TTC36	0.845955	0.780505	0.916894	4.67E-05
FTCD	0.851161	0.794185	0.912224	5.14E-06
F12	0.887832	0.825486	0.954887	0.001362
C8G	0.859275	0.779674	0.947003	0.002229
C12orf75	1.171861	1.070853	1.282397	0.000564
H2AFX	1.479433	1.27189	1.72084	3.80E-07
TTR	0.908994	0.856151	0.965098	0.001793
AURKB	1.474298	1.292284	1.681947	7.75E-09
GAMT	0.850473	0.758316	0.953829	0.005644
RRM2	1.569556	1.365104	1.804629	2.44E-10
MARCKSL1	1.332901	1.186177	1.497774	1.37E-06
C8A	0.872079	0.808884	0.940212	0.000362
CYP4A11	0.885342	0.824775	0.950356	0.000756
BIRC5	1.493355	1.329296	1.677661	1.44E-11
LRRC1	1.328325	1.147251	1.537977	0.000146
TST	0.809385	0.692371	0.946175	0.007945
HAAO	0.841192	0.742572	0.952909	0.006565
FAM83D	1.548369	1.33467	1.796284	7.94E-09
AGRN	1.331743	1.157865	1.531733	5.99E-05
RGN	0.834741	0.758639	0.918476	0.000213
JAG1	1.052161	0.911757	1.214186	0.486561
LAMA5	1.073447	0.916969	1.256629	0.377957
C3P1	0.843251	0.780829	0.910664	1.39E-05
MFSD10	1.205691	1.054206	1.378944	0.006323
APOC1P1	0.895649	0.828173	0.968623	0.005821
SERPINH1	1.14924	1.003106	1.316662	0.044999
PFKFB1	0.856081	0.773577	0.947384	0.002653
ALDH2	0.723434	0.628767	0.832354	6.06E-06
ITIH3	0.918369	0.852927	0.988832	0.023962
C6	0.841037	0.779233	0.907743	8.77E-06
IGSF3	1.449834	1.263151	1.664107	1.28E-07
ABCC1	1.266368	1.092541	1.467852	0.001719
RDH16	0.857921	0.800055	0.919973	1.70E-05
PON3	0.870735	0.789588	0.960222	0.005551
SLC27A2	0.88438	0.810066	0.965513	0.006076
HPN	0.819965	0.745121	0.902327	4.81E-05
AURKA	1.439667	1.254226	1.652525	2.22E-07
PCK2	0.836885	0.749323	0.934679	0.001589
HFE2	0.896882	0.835093	0.963243	0.002806
A1BG	0.851321	0.79204	0.91504	1.24E-05
MST1	0.858565	0.771087	0.955966	0.005414
C1R	0.786988	0.691528	0.895627	0.000283
TFR2	0.877951	0.807642	0.954381	0.002241
TEAD2	1.188049	1.040597	1.356394	0.010817
HSD17B6	0.889775	0.834524	0.948684	0.000356
PRAP1	0.949183	0.885562	1.017374	0.140653
DBN1	1.152952	1.024387	1.297652	0.018305
CYP4F2	0.874655	0.807718	0.947138	0.000977
CKS2	1.58371	1.357068	1.848203	5.39E-09
C4BPB	0.875697	0.799569	0.959073	0.00423
KNG1	0.891702	0.84093	0.945539	0.000127
FMNL2	1.120855	0.959214	1.309735	0.151032
FETUB	0.908059	0.847861	0.972532	0.005855
ITIH1	0.888562	0.830038	0.951214	0.000677
NT5DC2	1.28691	1.142545	1.449516	3.25E-05
DDR1	1.095005	0.986397	1.215572	0.088574
MCAM	1.147148	0.989866	1.32942	0.068064
PCK1	0.912018	0.859884	0.967312	0.002165
MAPK13	1.095778	0.980148	1.22505	0.107934
AMBP	0.904961	0.846268	0.967725	0.003513
GLS	1.218106	1.044635	1.420385	0.011834
SLC38A1	1.275461	1.133088	1.435723	5.60E-05
LEAP2	0.852932	0.781715	0.930638	0.000349
TNFRSF21	1.20762	1.076913	1.354191	0.001248
CENPM	1.439395	1.270029	1.631348	1.18E-08
CENPW	1.445459	1.255188	1.664573	3.12E-07
SERPINA11	0.921994	0.857015	0.9919	0.029401
HGD	0.889748	0.810945	0.976209	0.013555
SLC6A6	1.143847	0.986105	1.326822	0.075873
SPHK1	1.130617	1.035591	1.234363	0.00613

Supplementary Table S4 Continued

Gene	HR	lower_95	upper_95	*P*
BDH1	0.773519	0.693723	0.862494	3.78E-06
RBP5	0.855871	0.779292	0.939976	0.001137
APOA1	0.913452	0.867739	0.961573	0.000548
LPCAT1	1.517387	1.326342	1.73595	1.25E-09
FGA	0.899486	0.841581	0.961376	0.001808
PEMT	0.903923	0.773375	1.056507	0.204347
FLNA	1.077118	0.961768	1.206302	0.198638
CDKN3	1.425579	1.248702	1.62751	1.55E-07
ORM2	0.921514	0.856084	0.991945	0.029615
CDO1	0.867497	0.810517	0.928482	4.12E-05
PIPOX	0.885035	0.811391	0.965362	0.005865
AMDHD1	0.86833	0.782751	0.963266	0.007655
F11	0.778243	0.701774	0.863045	2.02E-06
CLIP2	1.166458	1.011395	1.345295	0.034375
SARDH	0.812023	0.713166	0.924582	0.001667
HAO1	0.868479	0.807506	0.934055	0.000147
STK39	1.247353	1.091879	1.424964	0.001137
F2	0.910605	0.847207	0.978747	0.010977
FGB	0.896775	0.842306	0.954767	0.000655
GYS2	0.834983	0.768289	0.907468	2.18E-05
PROZ	0.847426	0.767874	0.93522	0.000996
CFHR3	0.811426	0.755057	0.872005	1.28E-08
SLC1A5	1.303029	1.180541	1.438226	1.48E-07
UGT2B10	0.911845	0.850783	0.97729	0.009066
TM6SF2	0.829909	0.732419	0.940376	0.003454
ACMSD	0.916438	0.8236	1.019742	0.109327
CYP27A1	0.822752	0.746261	0.907083	8.90E-05
GLIS2	1.187982	1.025954	1.3756	0.02131
C1S	0.794676	0.707799	0.892217	1.00E-04
TES	1.154295	0.998168	1.334842	0.052962
FAM129B	1.147916	1.006165	1.309638	0.040233
ADH1C	0.868741	0.825061	0.914733	8.99E-08
MAT1A	0.90371	0.834839	0.978263	0.012303
NRSN2	1.197342	1.068039	1.342299	0.002009
GNMT	0.877729	0.817729	0.942132	0.000306
APOC4	0.803469	0.722411	0.893622	5.51E-05
AKR7A3	0.865589	0.801952	0.934275	0.000211
E2F1	1.370113	1.217171	1.542273	1.85E-07
AFM	0.841951	0.788315	0.899236	3.02E-07
BLMH	1.284305	1.106258	1.491007	0.001015
ABCB4	0.859906	0.785594	0.941248	0.001064
APOC2	0.905978	0.834034	0.984127	0.019337

Gene	HR	lower_95	upper_95	*P*
GMNN	1.184466	1.032703	1.358533	0.015523
ASL	0.891528	0.770429	1.031662	0.123199
TMPRSS6	0.837448	0.769834	0.911	3.63E-05
UPB1	0.833127	0.770056	0.901364	5.48E-06
SLC25A47	0.929701	0.877253	0.985284	0.01388
ALDH8A1	0.812994	0.741912	0.890886	9.21E-06
SERPINA10	0.868497	0.791371	0.953141	0.002964
CYP2D6	0.900165	0.836102	0.969136	0.005234
TMEM176B	0.927592	0.828173	1.038947	0.193798
SLC13A5	0.915074	0.848956	0.98634	0.020373
SERPINA6	0.886509	0.813692	0.965843	0.005874
ACOX2	0.877643	0.785405	0.980715	0.02124
CES2	0.867056	0.787316	0.954873	0.003755
CYP2C8	0.879038	0.828499	0.93266	1.98E-05
ORM1	0.917916	0.856535	0.983697	0.01529
ASGR1	0.885153	0.809525	0.967845	0.007424
ITGA3	1.129339	1.008866	1.264197	0.034572
PFKFB3	1.151312	1.035717	1.279808	0.009053
RARRES2	0.854319	0.769037	0.949058	0.003342
PTTG1	1.500046	1.322988	1.7008	2.49E-10
EHF	1.047834	0.9325	1.177432	0.432254
ADH1B	0.875156	0.825983	0.927257	6.19E-06
CYP2J2	0.944889	0.839916	1.062983	0.345458
APOE	0.826037	0.747831	0.912423	0.000166
FGG	0.910235	0.849274	0.975572	0.007832
INSIG1	0.909033	0.826277	1.000078	0.050187
HMGCS2	0.89112	0.835237	0.950742	0.000486
SEC14L2	0.812807	0.736413	0.897125	3.86E-05
FBLIM1	1.121998	0.984651	1.278504	0.084026
PYCR1	1.156696	1.060857	1.261193	0.000971
ADH1A	0.882655	0.830819	0.937724	5.29E-05
EPHX1	0.908914	0.825573	1.000669	0.051614
SAA2	0.945331	0.896867	0.996415	0.036284
PROC	0.86067	0.784051	0.944776	0.00161
SERPINA4	0.919353	0.852487	0.991464	0.029074
CYP8B1	0.896843	0.847617	0.948928	0.000157
FABP1	0.959137	0.905252	1.01623	0.157293
ITGB4	1.0937	0.97338	1.228893	0.132008
LAPTM4B	1.386235	1.233122	1.558359	4.52E-08
LRG1	0.92772	0.84289	1.021086	0.125162
PLG	0.900989	0.843297	0.962628	0.002014
GPD1	0.877422	0.79748	0.965378	0.007299

Supplementary Table S4 Continued

Gene	HR	lower_95	upper_95	*P*
ARG1	0.944909	0.885912	1.007834	0.084939
CYP4F3	0.869504	0.786748	0.960964	0.006139
GAL3ST1	1.12933	1.030151	1.238057	0.009504
SLC38A3	0.885166	0.819027	0.956647	0.00208
C3	0.851348	0.782503	0.926249	0.000184
C4BPA	0.89394	0.839035	0.952438	0.000527
SERPINF2	0.877346	0.815481	0.943903	0.000452
APOH	0.934433	0.879609	0.992675	0.027928
BAAT	0.87359	0.806141	0.946682	0.000979
ALB	0.9176	0.861815	0.976996	0.007205
GADD45G	0.847611	0.764068	0.940289	0.001791
CTSC	1.260128	1.11177	1.428284	0.000297
SDS	0.933667	0.885482	0.984474	0.011124
VTN	0.88916	0.822938	0.960711	0.00293
ASGR2	0.887152	0.814073	0.966791	0.006334
GSTA1	0.944583	0.891064	1.001316	0.055395
PAPLN	1.062316	0.935356	1.206508	0.351915
PGLYRP2	0.881582	0.820617	0.947077	0.000567
NDRG1	1.403065	1.254661	1.569022	2.90E-09
DPYS	0.882072	0.82138	0.947247	0.000561
SHMT1	0.82168	0.733146	0.920907	0.000734
TK1	1.349555	1.190137	1.530327	2.95E-06
CYP2C9	0.853493	0.803129	0.907016	3.31E-07
APOA2	0.950565	0.898397	1.005762	0.07833
IGF2BP2	1.191318	1.063905	1.33399	0.002419
F10	0.953358	0.849002	1.07054	0.419348
SULT1A2	0.947591	0.841354	1.067243	0.37492
CFHR1	0.91181	0.861251	0.965337	0.001514
PLBD1	1.257787	1.128405	1.402004	3.45E-05
SULT2A1	0.904334	0.853297	0.958423	0.000692
CYP2B6	0.928652	0.862335	1.000068	0.050211
PAFAH1B3	1.289242	1.148887	1.446744	1.56E-05
COL5A2	1.096405	0.966344	1.243971	0.153128
DNASE1L3	0.71604	0.634104	0.808563	7.16E-08
HAO2	0.878572	0.818661	0.942869	0.000328
ASS1	0.867204	0.767823	0.979449	0.021771
SEL1L3	1.095302	0.980888	1.223062	0.105846
CPS1	0.893046	0.847394	0.941157	2.39E-05
ZNF83	1.124201	0.987611	1.279681	0.076503
CPB2	0.856373	0.794777	0.922743	4.68E-05
OSGIN1	0.96687	0.876862	1.066118	0.499186
KCTD17	1.244337	1.102281	1.404701	0.000409

Gene	HR	lower_95	upper_95	*P*
ADH4	0.88024	0.839063	0.923438	1.80E-07
APOC41	0.803469	0.722411	0.893622	5.51E-05
ITM2C	1.101164	0.973879	1.245084	0.124137
LCAT	0.733005	0.654222	0.821275	8.61E-08
PFN2	1.283036	1.157963	1.421618	1.91E-06
UGT2B7	0.944527	0.882215	1.011239	0.101216
ACSM5	0.889234	0.817776	0.966935	0.006021
TMEM176A	0.988693	0.876646	1.115061	0.852999
SAA1	0.939879	0.897313	0.984465	0.008739
ANGPTL3	0.922026	0.855315	0.99394	0.034125
BAMBI	1.226982	1.10026	1.368299	0.000235
PFKP	1.199927	1.085233	1.326744	0.000377
PRODH2	0.965893	0.883387	1.056103	0.446208
ROBO1	1.175788	1.045715	1.32204	0.006784
TMED3	1.112712	0.990696	1.249756	0.071509
KHK	0.916919	0.822024	1.022769	0.119694
FXYD1	0.925628	0.853957	1.003315	0.06018
CFH	0.847059	0.768569	0.933564	0.000821
FKBP10	1.080208	0.975937	1.19562	0.13631
H2AFY2	1.080937	0.969518	1.205161	0.160845
SPINT1	1.012634	0.936807	1.094598	0.751886
SLC25A18	0.953637	0.859772	1.05775	0.369204
COL4A1	1.068293	0.946116	1.206248	0.286379
AQP9	0.894909	0.841222	0.952023	0.000436
FBLN1	1.107907	0.999963	1.227503	0.050083
F7	0.858551	0.774656	0.951533	0.00365
S100A11	1.139368	1.042256	1.245528	0.004098
SHBG	0.956177	0.874046	1.046026	0.328097
CYP4F11	0.953039	0.857483	1.059244	0.372247
UGT2B15	0.880945	0.826823	0.93861	8.91E-05
TSPAN15	1.11595	0.997269	1.248755	0.055839
SDSL	0.958179	0.846242	1.084923	0.500311
ANGPTL4	0.936829	0.85113	1.031157	0.182483
RAP1GAP	1.221776	1.09492	1.363329	0.000342
XDH	0.850512	0.762316	0.948912	0.003746
APCS	0.882405	0.831661	0.936245	3.47E-05
REEP6	0.918027	0.834533	1.009874	0.078747
IGFBP1	0.981645	0.917565	1.0502	0.590667
COL6A3	1.029301	0.914104	1.159015	0.633432
EHHADH	0.850585	0.771312	0.938006	0.001186
AKR1C4	0.992763	0.915527	1.076515	0.860478
HSD17B13	0.895362	0.844615	0.949159	0.000205

Supplementary Table S4 Continued

Gene	HR	lower_95	upper_95	*P*
NAT8	0.978661	0.890735	1.075267	0.653368
MFSD2A	0.912695	0.841253	0.990205	0.028044
PMEPA1	1.072468	0.963568	1.193675	0.200322
SERPINE2	1.126475	1.013335	1.252248	0.027436
GLTPD2	0.92544	0.832971	1.028174	0.149114
CD24	1.118133	1.042814	1.198891	0.0017
PLP2	1.163735	1.043086	1.298339	0.006621
MGST1	1.019984	0.902859	1.152303	0.750529
RHOBTB1	1.059705	0.946302	1.186698	0.315285
SERPINA1	0.980797	0.882935	1.089506	0.717695
APOM	0.98761	0.906655	1.075793	0.775096
SLC38A4	0.873807	0.810422	0.94215	0.000447
SGCE	1.141376	1.030732	1.263897	0.011029
NECAB2	0.896848	0.813908	0.988239	0.027884
SLC6A8	1.166001	1.06326	1.278671	0.001101
BHMT	0.911025	0.856863	0.968612	0.002885
CDHR5	0.919431	0.843069	1.002709	0.057589
HGFAC	0.895496	0.840732	0.953827	0.000608
FGGY	0.850775	0.763486	0.948043	0.003434
SERPIND1	0.933994	0.869443	1.003338	0.061657
C9	0.960176	0.912732	1.010085	0.115987
GPX3	1.013386	0.914078	1.123482	0.800506
KIF12	1.015073	0.912922	1.128654	0.782202
GLS2	0.889204	0.800527	0.987704	0.028467
ACSL1	0.876623	0.792586	0.969571	0.010438
ALDH1L1	0.974102	0.90727	1.045857	0.469331
GBP7	0.839995	0.76753	0.919302	0.000152
NR1I2	0.812304	0.73204	0.901368	9.00E-05
WBP5	1.150295	1.029535	1.28522	0.013349
DMGDH	0.82006	0.743339	0.904699	7.54E-05
IL32	0.896939	0.804622	0.999848	0.04968
ETNK2	0.920824	0.839798	1.009667	0.079217
LBP	0.931185	0.859677	1.008641	0.080305
SAA21	0.945331	0.896867	0.996415	0.036284
CP	0.989863	0.899005	1.089903	0.835681
FNDC5	0.89257	0.819869	0.971718	0.008746
AADAC	0.925557	0.852007	1.005457	0.067078
CTH	0.886951	0.813897	0.966562	0.00623
AHSG	0.948485	0.896207	1.003813	0.067491
TRNP1	1.247607	1.145515	1.358798	3.80E-07
FGL1	0.993813	0.924418	1.068418	0.866553
TF	0.959216	0.893736	1.029493	0.248404

Gene	HR	lower_95	upper_95	*P*
CYP2C18	0.940586	0.851763	1.038672	0.226182
NNMT	0.956511	0.898276	1.018522	0.165341
QSOX1	1.018265	0.92941	1.115615	0.69761
MT2A	0.917418	0.847271	0.993373	0.033687
CREB3L3	0.955649	0.872381	1.046865	0.329411
GPT2	0.847314	0.765147	0.938305	0.001455
CAPG	1.144185	1.040663	1.258004	0.005374
RGS2	1.122816	1.017747	1.238732	0.020839
CES1	0.984035	0.914999	1.058279	0.664529
SPP1	1.133569	1.08046	1.189288	3.04E-07
SLCO1B1	0.872062	0.809055	0.939976	0.000347
ETV4	1.201024	1.09108	1.322047	0.000184
LYZ	1.018655	0.953	1.088833	0.586612
TDO2	0.939595	0.87615	1.007636	0.080687
MT1X	0.910359	0.848701	0.976497	0.008674
IGFBP2	0.990586	0.914015	1.073571	0.81775
VIL1	1.096481	1.005977	1.195127	0.036123
MMP9	1.127497	1.035631	1.227511	0.005651
ST14	1.006148	0.913446	1.108257	0.901097
SPINT2	1.014773	0.934614	1.101806	0.726866
FMO3	0.853613	0.797749	0.913389	4.58E-06
SLC2A2	0.863044	0.803594	0.926892	5.24E-05
SERPINA7	0.90354	0.849764	0.960719	0.001195
RGS1	1.050077	0.950013	1.16068	0.338902
CDA	0.999394	0.904734	1.103957	0.99047
FST	1.013433	0.922145	1.113758	0.781732
S100A6	1.093066	1.014889	1.177265	0.018758
COL3A1	1.01546	0.93953	1.097527	0.698825
UGT2B4	0.973671	0.905038	1.047509	0.474348
COL1A2	1.024221	0.94302	1.112413	0.570122
PEG10	1.080863	1.023097	1.14189	0.005524
CYP3A4	0.909737	0.868194	0.953269	7.28E-05
IFI27	0.929199	0.866234	0.996741	0.040252
RAMP1	0.906177	0.833385	0.985326	0.021113
MT1A	0.965419	0.891473	1.045499	0.386712
TESC	1.065266	0.98793	1.148655	0.10014
COL1A1	1.026222	0.953568	1.104412	0.489626
MDK	1.104614	1.013578	1.203825	0.023371
HAL	0.96379	0.897625	1.034832	0.309443
CLDN4	1.124058	1.04711	1.206661	0.001228
ACSM1	0.958979	0.885455	1.038608	0.303392
AOX1	0.919647	0.859653	0.983829	0.014949

Supplementary Table S4 Continued

Gene	HR	lower_95	upper_95	*P*
CYP2E1	0.954401	0.914938	0.995565	0.030292
UGT1A4	0.960977	0.896623	1.029949	0.26036
MT1E	0.948207	0.891194	1.008866	0.092772
MT1G	0.958386	0.908905	1.01056	0.116048
GPC3	1.044524	0.991219	1.100695	0.103111
CRP	1.00116	0.960292	1.043767	0.956524
METTL3	1.753487	1.269469	2.422048	0.000655
METTL14	0.85615	0.615978	1.189966	0.355188
RBM15	1.69741	1.147642	2.51054	0.00806
RBM15B	2.090814	1.519908	2.876163	5.82E-06
WTAP	1.525273	1.13433	2.050954	0.005203
KIAA1429	1.69112	1.262828	2.264668	0.000422
CBLL1	1.524811	1.122377	2.071539	0.006967
ZC3H13	0.764748	0.644336	0.907662	0.002153
ALKBH5	1.115481	0.831707	1.496077	0.465606
FTO	1.085539	0.770465	1.52946	0.638909
YTHDC1	1.340223	0.938768	1.913356	0.106938
YTHDC2	0.858344	0.617614	1.192905	0.363038
YTHDF1	2.337959	1.659556	3.293685	1.19E-06
YTHDF2	2.533065	1.745011	3.677006	1.02E-06
YTHDF3	1.170491	0.886855	1.544841	0.266189
HNRNPA2B1	1.889195	1.343277	2.656977	0.000256
HNRNPC	1.873631	1.327247	2.644943	0.000358
FMR1	0.993255	0.787441	1.252864	0.954447
LRPPRC	2.240027	1.682217	2.9828	3.40E-08
ELAVL1	1.86179	1.28897	2.689172	0.000923
